# Synthesis and Antimicrobial Evaluation of New 1,2,4-Triazolo[1,5-*a*]pyrimidine-Based Derivatives as Dual Inhibitors of Bacterial DNA Gyrase and DHFR

**DOI:** 10.1021/acsomega.4c08365

**Published:** 2024-11-11

**Authors:** Lamya H. Al-Wahaibi, Safwat M. Rabea, Mohamed A. Mahmoud, Bahaa G.M. Youssif, Stefan Bräse, Salah A. Abdel-Aziz

**Affiliations:** 1 Department of Chemistry, College of Sciences, Princess Nourah bint Abdulrahman University, Riyadh 11671, Saudi Arabia; 2 Medicinal Chemistry Department, Faculty of Pharmacy, Minia University, Minia 61519, Egypt; 3 Department of Pharmaceutical Organic Chemistry, Faculty of Pharmacy, Assiut University, Assiut 71526, Egypt; 4 Institute of Biological and Chemical Systems, IBCS-FMS, Karlsruhe Institute of Technology, Karlsruhe 76131, Germany; 5 Department of Pharmaceutical Medicinal Chemistry and Drug Design, Faculty of Pharmacy (Boys), Al-Azhar University, Assiut 71524, Egypt; 6 Department of Pharmaceutical Chemistry, Faculty of Pharmacy, Deraya University, Minia 61519, Egypt

## Abstract

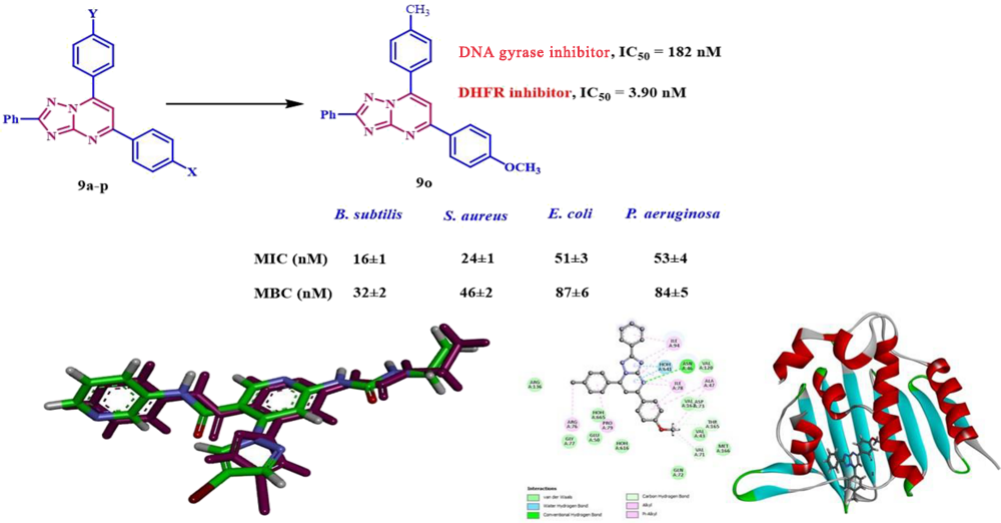

A series of 1,2,4-triazolo[1,5-*a*]pyrimidine-based derivatives were developed and prepared by reacting chalcones **8a**–**p** with 3-phenyl-1,2,4-triazole-5-amine (**5**). The novel compounds were analyzed using several spectroscopic techniques, and their antimicrobial efficacies against six pathogens (Gram-negative, Gram-positive, and fungi) were tested. Most of the tested compounds exhibited significant antimicrobial activity compared to ciprofloxacin and fluconazole. Four compounds (**9d**, **9n**, **9o**, and **9p**) showed promising results. Their minimal inhibitory concentration (MIC) values were between 16 and 102 μM, similar to ciprofloxacin’s 10–90 μM values. MIC values against the tested fungal species were between 15.50 and 26.30 μM, higher than fluconazole’s 11.50–17.50 μM values. Compounds **9n** and **9o**, in particular, showed excellent bactericidal activity. Compounds **9n** and **9o**, the most effective antibacterial agents, were further evaluated for their inhibitory effects on bacterial DNA gyrase and DHFR enzymes as possible molecular targets. The results indicated that **9n** and **9o** demonstrated a similar level of activity against DNA gyrase and DHFR when compared to the reference drugs ciprofloxacin and trimethoprim. We conducted molecular docking to investigate the binding mechanism and evaluate the reactivity of the intriguing compounds. Compounds **9n** and **9o** demonstrated favorable binding interactions with the essential amino acids necessary for the inhibition of *E. coli* DNA gyrase and DHFR enzymes.

## 1 Introduction

The global challenge of dealing with new bacterial diseases has gotten increasingly difficult due to the rising prevalence of multidrug resistance (MDR) microorganisms.^1,2^ This highlights the imperative necessity to develop novel, highly effective antibacterial agents. Multiple mechanisms contribute to mutations in microbial genomes, resulting in the development of resistance to established antibiotics. For example, abuse of antibiotics can lead to the emergence of resistant genotypes.^3,4^ Researchers are under pressure to find novel antimicrobial compounds as the incidence of infectious diseases and multidrug-resistant bacterial strains rises.^5^ The ultimate goal of medicinal chemistry is to develop new therapeutic compounds with superior pharmacological properties, drug tolerance, and reduced side effects.^6^

DNA gyrase has long been recognized as a promising target for antibacterial medicines.^7^ DNA gyrase belongs to the type II family of topoisomerases, which regulate the topological state of DNA in cells.^8^ DNA gyrase is a tetrameric enzyme comprising two subunits, GyrA and GyrB. The GyrB subunit breaks down ATP and causes DNA to supercoil negatively. This is necessary to maintain DNA structure during the replication process. DNA gyrase is a vital enzyme found in all types of bacteria. Blocking hinders the process of DNA production, ultimately leading to the death of the bacterial cells.^9^ In a therapeutic setting, two categories of antibiotics have proven gyrase to target DNA gyrase effectively. These categories include fluoroquinolones and aminocoumarins. Nevertheless, the increasing occurrence of antibiotic-resistant bacterial strains reduces the effectiveness of fluoroquinolone medications.^10^ Unfavorable pharmacokinetics and safety concerns have hindered the widespread use of the coumarin class of antibiotics.^11,12^

On the other hand, dihydrofolate reductase (DHFR) is a crucial component of the folate metabolic process. DHFR is essential for the biological production of RNA, DNA, and cellular proteins in different species.^13,14^ The enzyme is a promising focus for medicinal chemistry, and its inhibitors have been employed in treating severe ailments such as cancer, bacterial infections, malaria, tuberculosis, and others. Almost all cells contain DHFR. It plays a crucial role in biochemically sustaining the active state of the folate requirement.^15,16^ The dihydrofolate reductase enzyme converts 7,8-dihydrofolate to 5,6,7,8-tetrahydrofolate with the help of NADPH. This is the first step in making cofactors needed to make amino acids, thymidylate, and nucleotides like purines and pyrimidines.^17,18^ Thus, blocking the DHFR enzyme inhibits the DNA synthesis pathway. This inhibition is useful in various clinical settings because it causes rapidly growing cells to die.^19^

**Figure 1:**
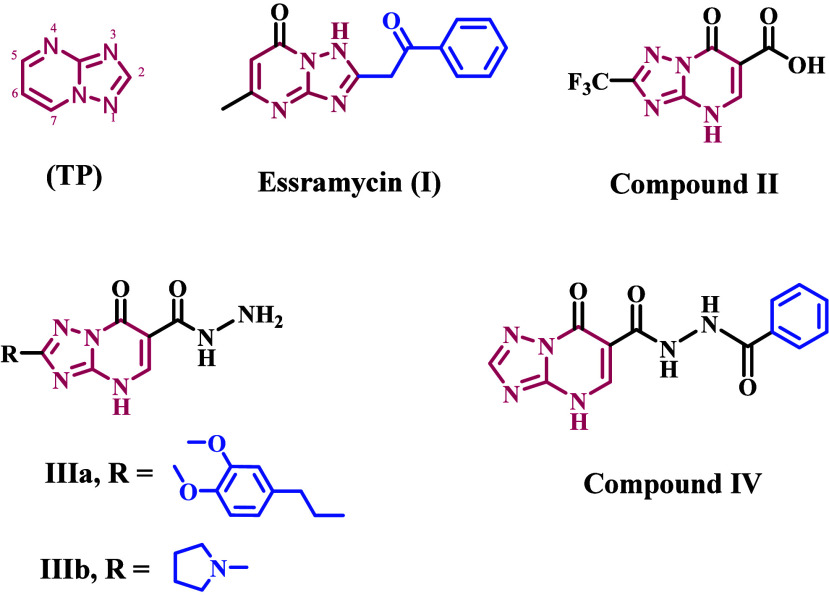
Structure of 1,2,4-triazolo[1,5-*a*]pyrimidine (**TP**) and compounds **I**–**IV**.

Bulow and Haas initially reported the 1,2,4-triazolo[1,5-*a*]pyrimidine heterocycle (TP, Figure 1).^20^ This scaffold can be found in natural products, like tobramycin (**I**, Figure 1), a TP derivative that was isolated from the broth of marine Streptomyces sp.^21^ However, most biologically active TP compounds are not in nature. Over time, the TP scaffold has found several applications in medicinal chemistry. Many studies have looked at TP derivatives as possible isosteric replacements for purines because they have structures similar to the purine ring. However, the TP heterocycle is a useful scaffold that can be used for more than just isosteric replacement techniques.^22^ It is often used to find biologically active molecules with good ADME-PK properties. Researchers have discovered numerous TP derivatives in recent years that show significant promise in a variety of therapeutic domains, including cancer treatment,^23,24^ neurological illnesses,^25^ and infectious diseases.^26,27^ Additionally, numerous TP derivatives have demonstrated potential as agrochemicals.^28^

Essramycin (**I**, Figure 1), the first 1,2,4-triazolo[1,5-*a*]pyrimidine antibiotic was found in the culture broth of marine Streptomyces species, shows significant activity against Gram-positive and Gram-negative bacteria, with MIC ranging from 2.0 to 8.0 μg/mL.^21^ At a 6.25 μg/mL dose, triazolopyrimidine-6-carboxylic acid (**II**, Figure 1) inhibited *M. tuberculosis* H37RV growth by 92%. It also had antibacterial action against *B. cereus* and was nontoxic to mammalian cells. Additionally, compounds with a hydrazide group in the sixth position, **IIIa** and **IIIb** (Figure 1), showed strong antibacterial activity against Gram-positive and Gram-negative bacterial strains, with MIC values as low as 2 μg/mL. Also, **IIIa** and **IIIb** had IC_50_ values of 61 μg/mL against Gram-positive strains and 40 μg/mL against Gram-negative strains in the DNA displacement experiment.^29^

A recent study^30^ explored the design, synthesis, and screening of a series of 1,2,4-triazolo[1,5-*a*]pyrimidine derivatives. The compounds’ efficiency against bacterial and fungal infections and their safety profile were evaluated. Many novel compounds have been developed that are highly effective at killing Gram-positive and Gram-negative bacteria, with minimum inhibitory concentrations (MICs) ranging from 0.25 to 2.0 μg/mL. Compound **IV** (Figure 1) outperformed ciprofloxacin in inhibiting DNA gyrase (IC_50_ = 0.68 μM vs 0.85 μM). Molecular docking at the active site of DNA gyrase revealed a binding mechanism and docking scores comparable to those of ciprofloxacin.

**Figure 2:**
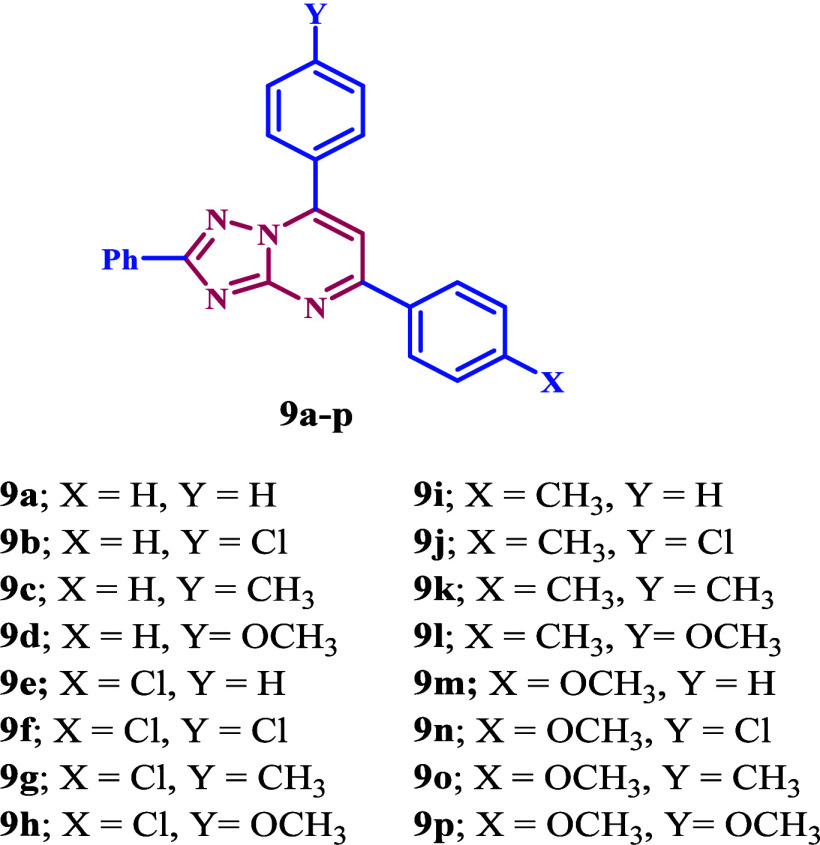
Structures of compounds **9a**–**p**.

Based on the information previously provided and our ongoing interest in developing new antimicrobials that target DNA gyrase and DHFR,^31−35^ we present the design, synthesis, and biological evaluation of a new group of 1,2,4-triazolo[1,5-*a*]pyrimidine-based derivatives **9a**–**p** (Figure 2) that inhibit bacterial DNA gyrase and DHFR enzymes. Compounds **9a**–**p** have been tested for antibacterial activity against two Gram-positive bacteria (*S. aureus* and *B. subtilis*), two Gram-negative bacteria (*E. coli* and *P. aeruginosa*), and two fungal strains (*A. flavus* and *C. albicans*), using ciprofloxacin and fluconazole as reference compounds. The most active compounds will have their minimum inhibitory concentration (MIC), and minimum bactericidal concentration (MBC) determined. We will additionally evaluate the most potent compounds as inhibitors of bacterial DNA gyrase and DHFR. Finally, molecular docking study will be conducted to assess their binding affinity to the target receptors.

## 2 Results and Discussion

### 2.1 Chemistry

**Scheme 1:**
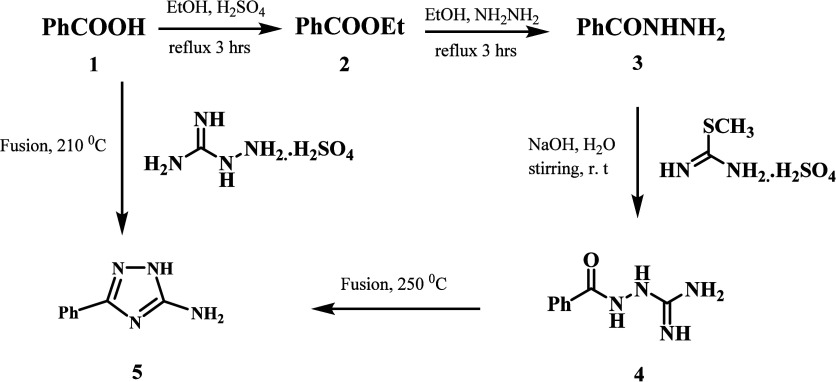
Synthesis of the Key Intermediate 3-Phenyl-1,2,4-triazole-5-amine **5**

Scheme 1 outlines the synthesis of target compounds (**9a**–**p**). The key intermediate 3-phenyl-1,2,4-triazole-5-amine (**5**) was synthesized by two different pathways. Ethanol and sulfuric acid first converted aromatic acid **1** into ethyl ester **2**. We then refluxed ethyl ester **2** with hydrazine hydrate to produce aromatic acid hydrazide **3**. Acid hydrazide **3** interacted with aqueous S-methylisourea sulfate in the presence of sodium hydroxide to form aroylaminoguanidine **4**, which underwent cyclization by fusion at 250 °C to yield 3-phenyl-1,2,4-triazole-5-amine **5**.^36^ We used an alternate approach of thermal condensation of aromatic acid **1** with aminoguanidine sulfate at 210 °C under solvent-free conditions to produce 3-phenyl-1,2,4-triazole-5-amine **5**.^37^

**Scheme 2:**
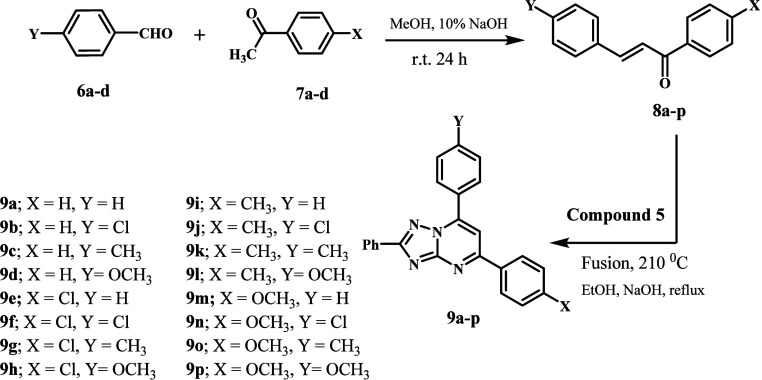
Synthesis of New Compounds **9a**–**p**

The synthesis of compounds **8a**–**p** and new compounds **9a**–**p** is outlined in Scheme 2. P*-*substituted acetophenones **6a**–**d** were reacted with aryl aldehydes **7a**–**d** in methanolic sodium hydroxide to afford chalcones **8a**–**p** in good yields.^38^ The new compounds **9a**–**p** were synthesized by cyclocondensation of amino triazole **5** with chalcone derivatives **8a**–**p**. The reaction unfolded in three stages. Initially, the aminotriazole’s nucleophilic amino group reacts with the chalcone carbonyl. Second, when triazole NH_2_ is introduced into the chalcone double bond, a cyclization reaction takes place. The procedure ends with the elimination of two hydrogens atoms, followed by aromatization.

The structures of the newly synthesized compounds **9a**–**p** were verified by ^1^H NMR, ^13^C NMR, LC-MS, and elemental analysis. Compound **9k** is a good example of this group. Its ^1^H NMR spectrum showed two singlet signals of three protons at δ 2.44 and 2.38 ppm, which are two methyl groups. The ^13^C NMR spectrum confirmed this, revealing two singlet signals at approximately δ 21.70 and 21.53 ppm. Furthermore, the spectrum revealed the distinctive signals of aromatic protons. Additionally, the LC-MS spectra revealed a signal at *m*/*z* 377.00, corresponding to [M + H]^+^.

### 2.2 Biology

#### 2.2.1 *In Vitro* Antimicrobial Assay

The antimicrobial properties of the target compounds (**9a**–**p**) were evaluated against two Gram-positive bacteria (*S. aureus* and *B. subtilis*), two Gram-negative bacteria (*E. coli* and *P. aeruginosa*), and two fungal strains (*A. flavus* and *C. albicans*). The modified disk diffusion method^39,40^ was used to determine the inhibition zones (IZ, mm/ml) and the minimal inhibitory concentration (MIC, nM). Ciprofloxacin and fluconazole were the positive controls used in the experiment.

The target compounds **9a**–**p** demonstrated significant antibacterial activity against both Gram-positive and Gram-negative bacteria. The inhibition zones (IZ) of all tested pathogens ranged from 9 to 45 mm, comparable to ciprofloxacin’s IZ of 40 mm. Notably, compounds **9d**, **9n**, **9o**, and **9p** exhibited larger or similar inhibition zones to those of the reference ciprofloxacin against the examined bacterial strains, as shown in Table 1. These findings not only shed light on the potential of these compounds as effective antibacterial agents but also inspire hope for the future of antibacterial research.

**Table 1:** Inhibition Zone Diameter of Compounds **9a**–**p**

	**Inhibition Zone (IZ) Diameter** (mm/mL)					
	**Bacterial species**					
	**(G**^**+**^**)**		**(G**^**–**^**)**		**Fungi**	
**Sample**	B. subtilis	S. aureus	E. coli	P. aeruginosa	A. flavus	C. albicans
**9a**	12	14	14	12	7	10
**9b**	21	23	23	24	11	15
**9c**	11	15	13	13	7	10
**9d**	35	35	33	34	27	30
**9e**	10	12	12	11	5	8
**9f**	14	15	15	18	8	12
**9g**	19	20	20	18	9.0	11
**9h**	9	11	11	12	5	7
**9i**	28	29	30	29	17	20
**9j**	20	21	20	20	10	13
**9k**	32	32	30	30	22	25
**9l**	23	26	23	22	15	17
**9m**	31	32	30	30	20	23
**9n**	40	41	40	40	32	36
**9o**	43	45	43	42	35	38
**9p**	38	39	37	38	30	31
**Ciprofloxacin**	40	40	40	40	NA	NA
**Fluconazole**	NA	NA	NA	NA	40	40

The compound **9o** (X = OMe, Y = Me) was identified as the most effective antibacterial agent in our study. It exhibited IZ values of 43 and 45 mm against Gram-positive pathogens *B. subtilis* and *S. aureus* and 43 and 42 mm against Gram-negative strains *E. coli* and *P. aeruginosa*, surpassing the reference ciprofloxacin (IZ = 40 mm/ml) against all species tested. Our findings not only suggest that the substitution pattern of the phenyl groups at positions 5 and 7 may indeed play a crucial role in the antibacterial action but also provide a deeper understanding of the role of the phenyl group in the antibacterial action. Compounds **9c** (X = H, Y = Me), **9g** (X = Cl, Y = Me), and **9k** (X = Me, Y = Me) are structurally similar to **9o**, but they have H, Cl, and Me groups at the para-position of the phenyl group at position 5 of the 1,2,4-triazolopyrimidine scaffold. These compounds exhibited weaker antibacterial activity than **9o**, with IZ values ranging from 11 to 15 mm for **9c**, 18–20 mm for **9g**, and 30–32 mm for **9k** (**9o**; IZ = 42–45 mm). This reiterates the importance of the phenyl group at the fifth position’s para-position in tolerating the methoxy group for antibacterial action and the order of reactivity as X = OMe > Me > Cl > H.

Also, the substitution pattern of the *para*-position of the phenyl group at position 7 may influence the antibacterial effect of **9a**–**p**. Compound **9n** (X = OMe, Y = Cl), which substitutes a chlorine atom for the methyl group in **9o**, demonstrated the second-highest activity against all tested species, with an IZ diameter of approximately 40 mm. Its activity was comparable to that of ciprofloxacin. Another example is compound **9p** (X = OMe, Y = OCH_3_), which is the methoxy derivative of **9o**. With IZ diameters of approximately 38 mm against the four species under examination, it exhibited nearly identical activity as **9n**. These results demonstrate that the antibacterial action tolerated methyl and methoxy groups, along with a chlorine atom at the *para*-position of the phenyl group at position 7, in the activity order Y = Me > Cl > OMe. The unsubstituted derivative at the para-position of the phenyl group at position 7, compound **9m** (X = OMe, Y = H), was the least active of the methoxy derivatives (**9m**–**p**). The IZ diameter of **9m**, of approximately 30 mm, versus the four studied species indicates that the H atom is not favored for antibacterial activity.

In addition, the newly synthesized compounds **9a**–**p** showed IZ diameter values ranging from 5 to 38 mm against *A. flavus* and *C. albicans*, whereas the reference fluconazole had a value of 40 mm. Once again, compounds **9d**, **9n**, **9o**, and **9p** exhibited the highest activity level against the fungal species tested. The diameter of the inhibition zones ranged from 27 to 38 mm. These findings from Table 1 indicate that the identical principles governing the antibacterial effect can also be applied to the antifungal effect. We experimented to determine the minimal inhibitory concentrations (MIC, nM) of the most potent derivatives **9d**, **9n**, **9o**, and **9p**. Tables 2 and 3 present the results.

**Table 2:** MIC and MBC (nM) Values of Compounds **9d**, **9n**, **9o**, and **9p**

	Bacterial species							
	**(G**^**+**^**)**				(G^–^)			
	B. subtilis		S. aureus		E. coli		P. aeruginosa	
Compound	***MIC***	***MBC***	***MIC***	***MBC***	***MIC***	***MBC***	***MIC***	*MBC*
**9d**	38 ± 2	64 ± 3	39 ± 2	71 ± 4	73 ± 5	102 ± 7	75 ± 5	109 ± 7
**9n**	29 ± 2	49 ± 2	33 ± 2	65 ± 2	65 ± 4	93 ± 6	64 ± 5	94 ± 6
**9o**	16 ± 1	32 ± 2	24 ± 1	46 ± 2	51 ± 3	87 ± 6	53 ± 4	84 ± 5
**9p**	35 ± 2	52 ± 3	37 ± 2	69 ± 3	69 ± 4	97 ± 6	68 ± 5	98 ± 6
**Ciprofloxacin**	10 ± 1	19 ± 1	30 ± 2	45 ± 2	60 ± 4	90 ± 6	60 ± 5	90 ± 6

**Table 3:** MIC Values of Compounds **9d**, **9n**, **9o**, and **9p** against *A. flavus* and *C. albicans*

	Fungi (μM)	
	A. flavus	C. albicans
Compound	***MIC***	***MIC***
**9d**	21.50 ± 1	26.30 ± 1
**9n**	18.50 ± 1	21.90 ± 1
**9o**	15.50 ± 1	19.50 ± 1
**9p**	19.90 ± 1	23.70 ± 1
**Fluconazole**	11.50 ± 1	17.50 ± 1

#### 2.2.2 Minimum Inhibitory Concentration (MIC) Assay

We assessed the antibacterial activity of the most potent components, **9d**, **9n**, **9o**, and **9p**, using a 2-fold serial dilution approach on a 96-well microtiter plate.^40^Table 2 presented these compounds’ MICs against the examined organisms, with ciprofloxacin as the reference medication. The outcomes of this *in vitro* assay test align with the results of the antimicrobial sensitivity test.

The compound **9o** (X = OMe, Y = CH_3_) was the most effective against *S. aureus*, *E. coli*, and *P. aeruginosa*, with MIC values of 24, 51, and 53 nM. It was more potent than ciprofloxacin against the tested species, although its MIC value against *B. subtilis* was 16 nM, 1.6 times less effective than ciprofloxacin (MIC = 10 nM). Compound **9n** (X = OMe, Y = Cl) had the second-highest activity level. As shown in Table 2, the tested compound had higher MIC values than ciprofloxacin, which means it was less effective against *S. aureus*, *E. coli*, and *P. aeruginosa*. Nevertheless, It exhibited 3 times lower efficacy than ciprofloxacin against *B. subtilis*. Among the tested species, compound **9p** (X = OMe, Y = OMe) was most effective against *S. aureus* and *P. aeruginosa*, with MICs of 69 and 68 nM, respectively. The data were similar to ciprofloxacin’s, with a MIC of 60 nM. Finally, compound **9d** (X = H, Y = OCH_3_) demonstrated the lowest efficacy. It has a lower antibacterial efficacy than ciprofloxacin against all pathogens examined.

#### 2.2.3 Minimum Bactericidal Concentration (MBC Assay)

The MBC differs from the MIC. The MIC test determines the minimum inhibitory concentration of an antimicrobial agent that effectively inhibits microorganism growth. In contrast, the MBC test reveals the minimum bactericidal concentration that leads to the death of these organisms. Unlike the MBC, which leads to mortality, the MIC hinders bacterial growth without causing death.^41^ The common expression for MBC is MBC_50_, signifying the antibiotic concentration that eliminates 50% of the initial bacterial population.^42^

Components **9d**, **9n**, **9o**, and **9p** exhibited potent bactericidal action overall. For Gram-positive bacteria, the MBC ranged from 32 to 71 nM. For ciprofloxacin, the MBC ranged from 19 to 45 nM, as shown in Table 2. Compounds **9o** (X = OMe, Y = CH_3_) and **9n** (X = OMe, Y = Cl) are the most effective antibacterial agents, demonstrating bactericidal activity of 32 and 49 nM, respectively, against *B. subtilis*, compared to ciprofloxacin’s MBC value of 19 nM, being 1.7- and 2.6-folds less potent than ciprofloxacin as a bactericidal agent. Compound **9o** exhibited an MBC_50_ value of 46 nM against *S. aureus*, indicating that it was almost as efficient as ciprofloxacin (MBC_50_ = 46 nM) in killing the organism tested.

In the case of Gram-negative bacteria, compound **9o** had the lowest MBC values among the compounds tested against *E. coli* and *P. aeruginosa*. This compound has MBC values of 87 and 84 nM, respectively, which makes it more efficient than ciprofloxacin (MBC = 90 nM) against *E. coli* and *P. aeruginosa* species. Compounds **9n** (X = OMe, Y = Cl) and **9p** (X = OMe, Y = OMe) demonstrated significant bactericidal action. Their MBC values were 93 and 97 nM for *E. coli* and 94 and 98 nM for *P. aeruginosa*, respectively, similar to ciprofloxacin’s value of 90 nM. This means that **9n** and **9p** are also effective at killing bacteria. Compound **9d** (X = H, Y = OCH_3_) was the least effective bactericidal agent. It has poorer efficacy than ciprofloxacin against all tested pathogens.

#### 2.2.4 Antifungal Assay

We evaluated the antifungal activity of compounds **9d**, **9n**, **9o**, and **9p** using a 2-fold serial dilution approach.^43^Table 3 shows these compounds’ MICs (μM) against *A. flavus* and *C. albicans* fungi, with fluconazole as the reference drug.

Compared to fluconazole, the studied compounds demonstrated moderate to good antifungal efficacy against the selected fungus species. Compounds **9d**, **9n**, **9o**, and **9p** reported MIC values of 15.50 to 21.50 μM against *A. flavus*, while fluconazole had a MIC of 11.50 μM. The studied compounds have MIC values ranging from 19.50 to 26.50 μM against *C. albicans*, with the reference fluconazole having a MIC of 17.50 μM. Compound **9o** (R = OMe, X = CH_3_) exhibited high potency as an antibacterial and antifungal agent. It possessed MIC values of 15.50 μM against *A. flavus* and 19.50 μM against *C. albicans*, similar to the MIC values of the reference fluconazole (11.50 and 17.50 μM, respectively). The relatively low antifungal efficacy of the investigated compounds did not motivate us to evaluate their MBC values.

#### 2.2.5 DNA Gyrase and DHFR Inhibitory Assay

The inhibitory efficacy of the most potent antibacterial agents, derivatives **9d**, **9n**, **9o**, and **9p**, was assessed against *E. coli* DNA gyrase and DHFR.^44^Table 4 presents the results as IC_50_ values for the tested compounds and the reference medicines (Novobiocin and Trimethoprim).

**Table 4:** IC_50_ Values of Compounds **9d**, **9n**, **9o**, and **9p** against DNA Gyrase and DHFR Enzymes

	IC_50_ (μM)	
Compound	E. coli **DNA gyrase**	**DHFR** E. coli
**9d**	217 ± 13	5.90 ± 0.20
**9n**	190 ± 11	4.60 ± 0.20
**9o**	182 ± 10	3.90 ± 0.10
**9p**	205 ± 13	5.40 ± 0.20
**Novobiocin**	170 ± 10	--
**Trimethoprim**	--	5.20 ± 0.20

The results of this assay were consistent with the antibacterial activity findings. Compound **9n** (X = OMe, Y = Cl) and **9o** (X = OMe, Y = Me), the most potent antibacterial agents, demonstrated inhibitory effects on *E. coli* DNA gyrase, with IC_50_ values of 197 and 185 nM, respectively. These values were compared to the reference compound novobiocin, which had an IC_50_ value of 170 nM. However, compounds **9d** (X = H, Y = OMe) and **9p** (X = OMe, Y = OMe) had a noteworthy inhibitory impact on DNA gyrase, with IC_50_ values of 217 and 205, respectively. These values indicate that they are less effective than novobiocin.

Using Trimethoprim as a reference medication, we further tested Compounds **9d**, **9n**, **9o**, and **9p** for their inhibitory efficacy against the DHFR enzyme. The DHFR enzyme was strongly blocked by compounds **9n** and **9o**, with IC_50_ values of 4.60 and 3.90 μM, respectively, lower than the IC_50_ value of 5.20 for trimethoprim. Compounds **9n** and **9o** were more powerful than the reference trimethoprim and had significant antibacterial action. Compounds **9d** and **9p** showed IC_50_ values of 5.90 and 5.40 μM, respectively. These values were comparable to the reference trimethoprim (IC_50_ = 5.20 μM). These findings demonstrate the potential antibacterial activity of compounds **9n** and **9o** as dual inhibitors of DNA gyrase and DHFR enzymes, which may necessitate structural modifications to produce more potent derivatives with improved pharmacologic and pharmacokinetic properties.

### 2.3 Molecular Docking Study

#### 2.3.1 *E. coli* DNA Gyrase B

**Figure 3:**
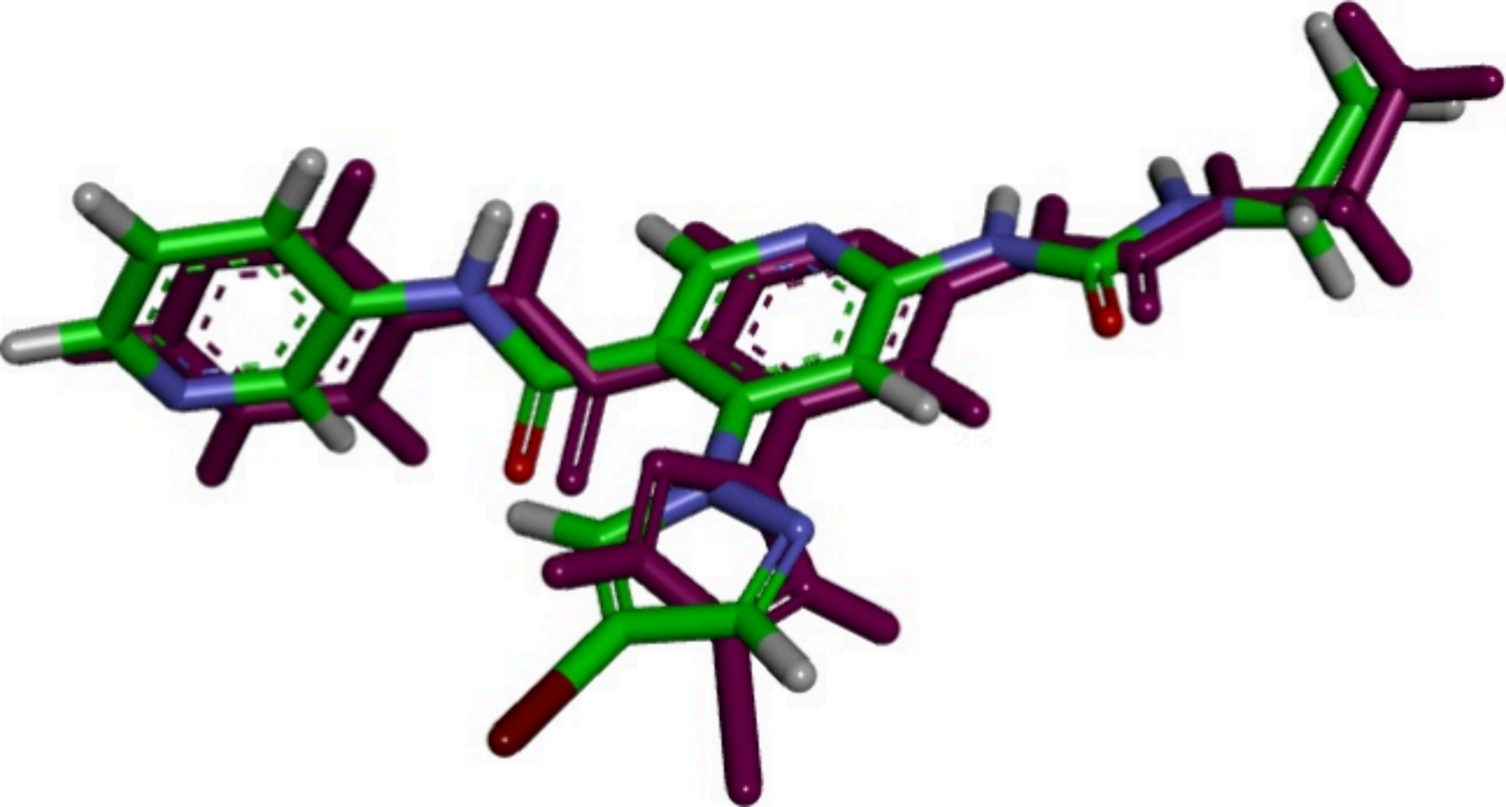
Validation of the accuracy and performance of docking process for *E. coli* DNA gyrase. The docked ligand inhibitor (purple) and native ligand inhibitor (green) demonstrated an RMSD of 1.38 Å.

Docking analysis was used to evaluate the potential binding interactions of the newly synthesized compounds within the *E. coli* DNA gyrase B active site, predicting their binding pattern and determining their capacity to satisfy the necessary structural properties for binding interactions. Autodock 4.2^45^ was used to dock compounds **9b** and **9o** into the crystal structure of the active site of *E. coli* DNA gyrase B complexed with the pyridine-3-carboxamide ligand inhibitor (PDB ID: 6F86).^46^ We first verified the docking setup by self-docking the cocrystallized thiazole inhibitor in the active site of *E. coli* DNA gyrase B. The self-docking validation recreated the cocrystallized ligand inhibitor, proving that the docking method was right for the planned research, Figure 3. The minimal RMSD of 1.38 between the experimental cocrystallized inhibitor pose and the docked pose, along with the docking pose’s ability to replicate all essential interactions made by the cocrystallized ligands at the active site, demonstrate this.

**Figure 4:**
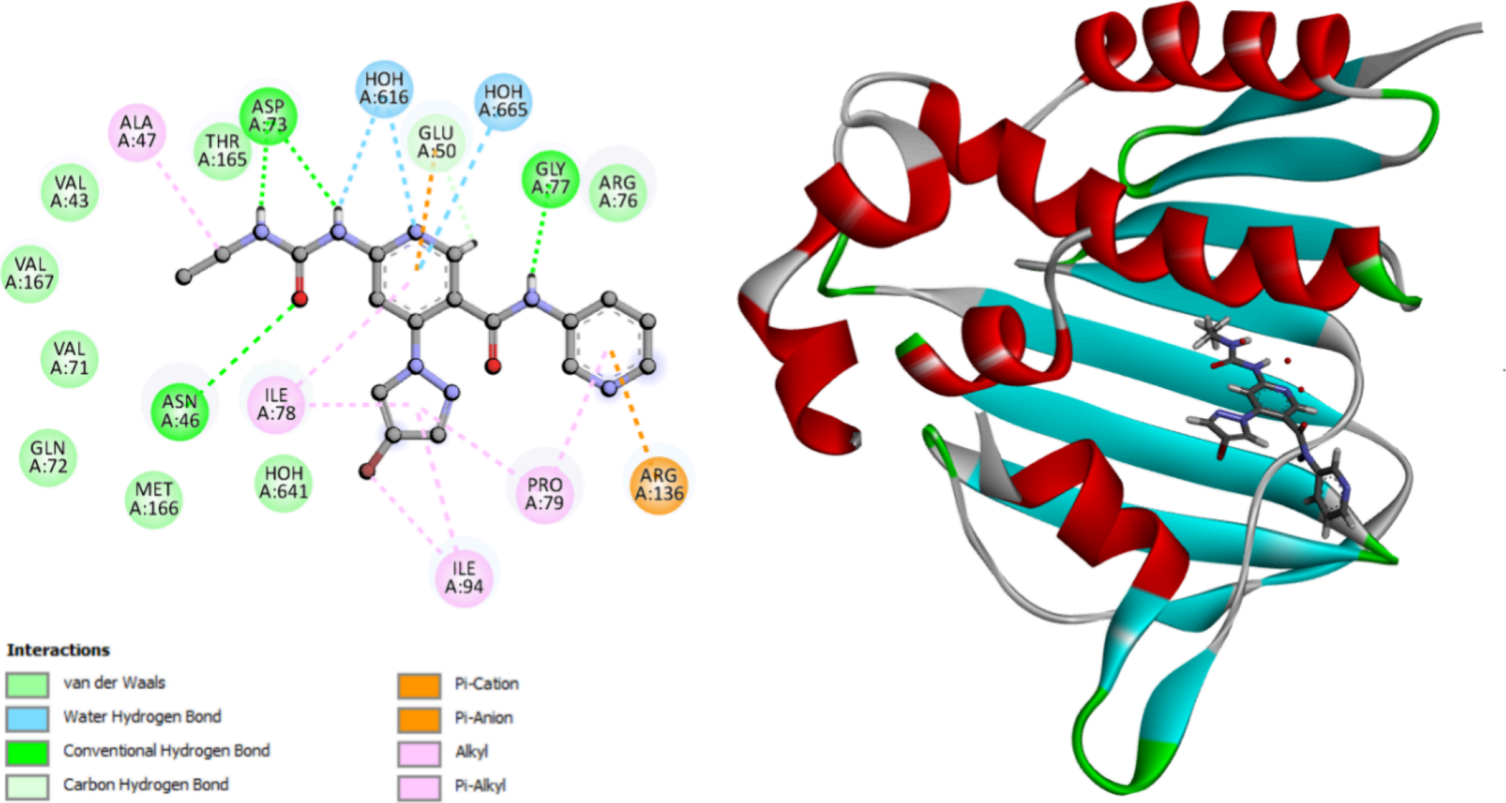
2D diagram representation of (left side) and 3D diagram representation (right side) of thiazole inhibitor docked into *E. coli* DNA gyrase B active site showing his binding interactions with the amino acids binding site.

The calculated docking energy score was S = −11.76 kcal/mol. Asn46 forms hydrogen bonds with the oxygen function of urea, while Asp73 interacts with the two nitrogen atoms of urea via hydrogen bonding. The pyridine ring of *N*-pyridine carboxamide interacts with Arg136 as a π-cation, and the pyridine ring of pyridine carboxamide interacts with Glu50 as a π-anion. The remaining amino acids in the pocket, including Arg76, Pro79, Ile94, Ile78, Ala47, Val71, Asp73, Gly77, Gln72, Met166, Val43, Thr165, Val120, and Val167, interact with the methoxy and substituted triazolopyrimidine functional groups through π-alkyl, alkyl, or van der Waals interactions, see Figure 4 and Table 5.

**Table 5:** Binding Scores, Amino Acid Interactions, and Bond Lengths of the Selected Compounds within the Active Site of *E. coli* DNA Gyrase B

		Interacting residues		
			Hydrophobic interaction	
Compound	Binding score (S value) kcal/mol	H-bonding	π-cation, π-anion, π-alkyl interactions	van der Waals interactions
**9n**	–11.42	Asn46	Arg76, Pro79, Ile94, Ile78, Ala47, Val71, Asp73	Gly77, Glu50, Gln72, Met166, Val43, Thr165, Val120, Val167
**9o**	–11.28	Asn46	Arg76, Pro79, Ile94, Ile78, Ala47, Val71, Asp73	Arg136, Gly77, Glu50, Gln72, Met166, Val43, Thr165, Val120, Val167,
**Ligand inhibitor**	–11.76	GLY77, Asn46, ASP73	Glu50, Arg136, Ala47, Pro79, Ile94, Ile78	Met166, Gln72, Val71, Val167, Val43, Thr165, Arg76

**Figure 5:**
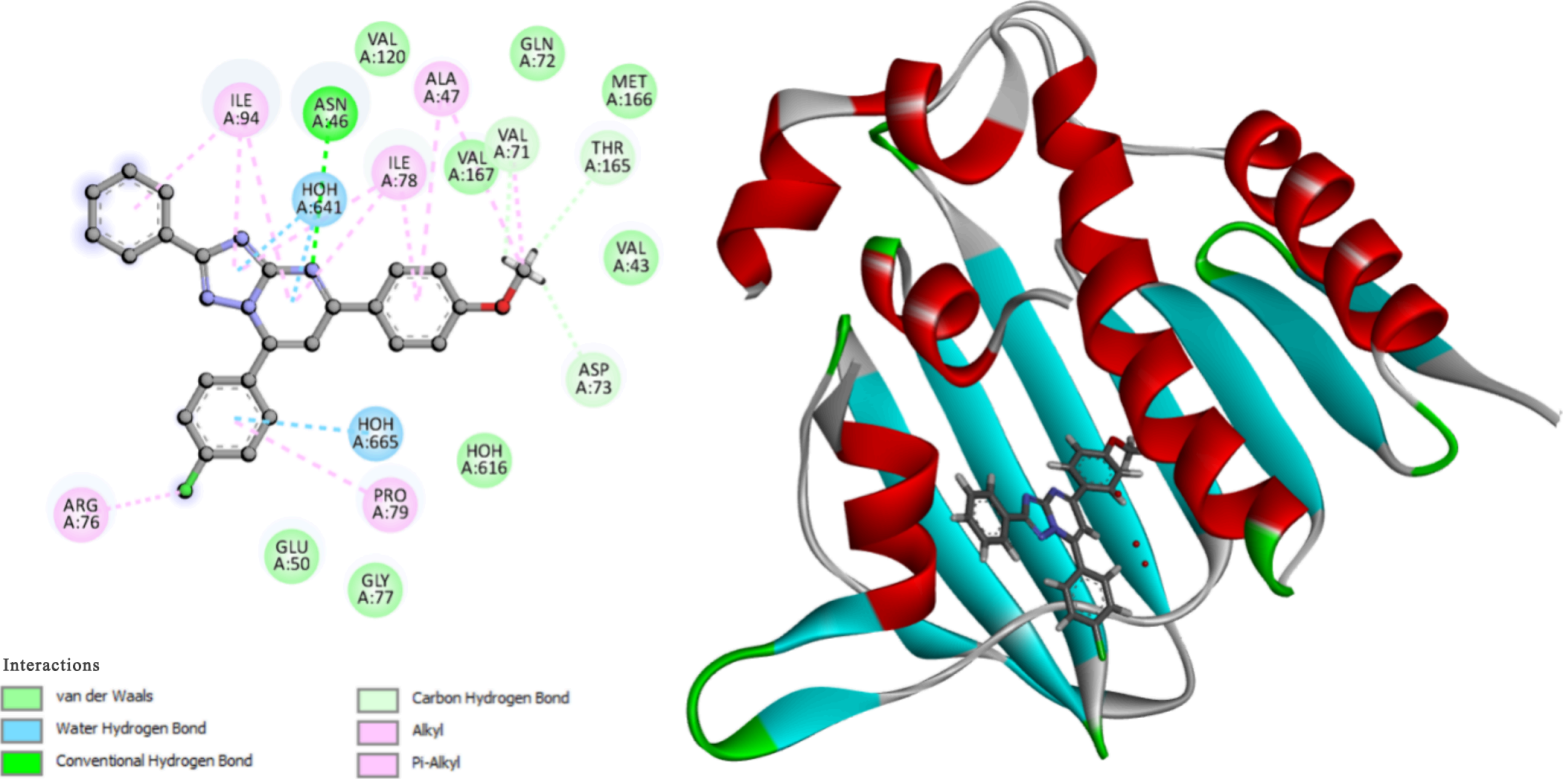
2D diagram representation (left side) and 3D diagram representation (right side) of compound **9n** docked into *E. coli* DNA gyrase B active site showing his binding interactions with the amino acids binding site.

**Figure 6:**
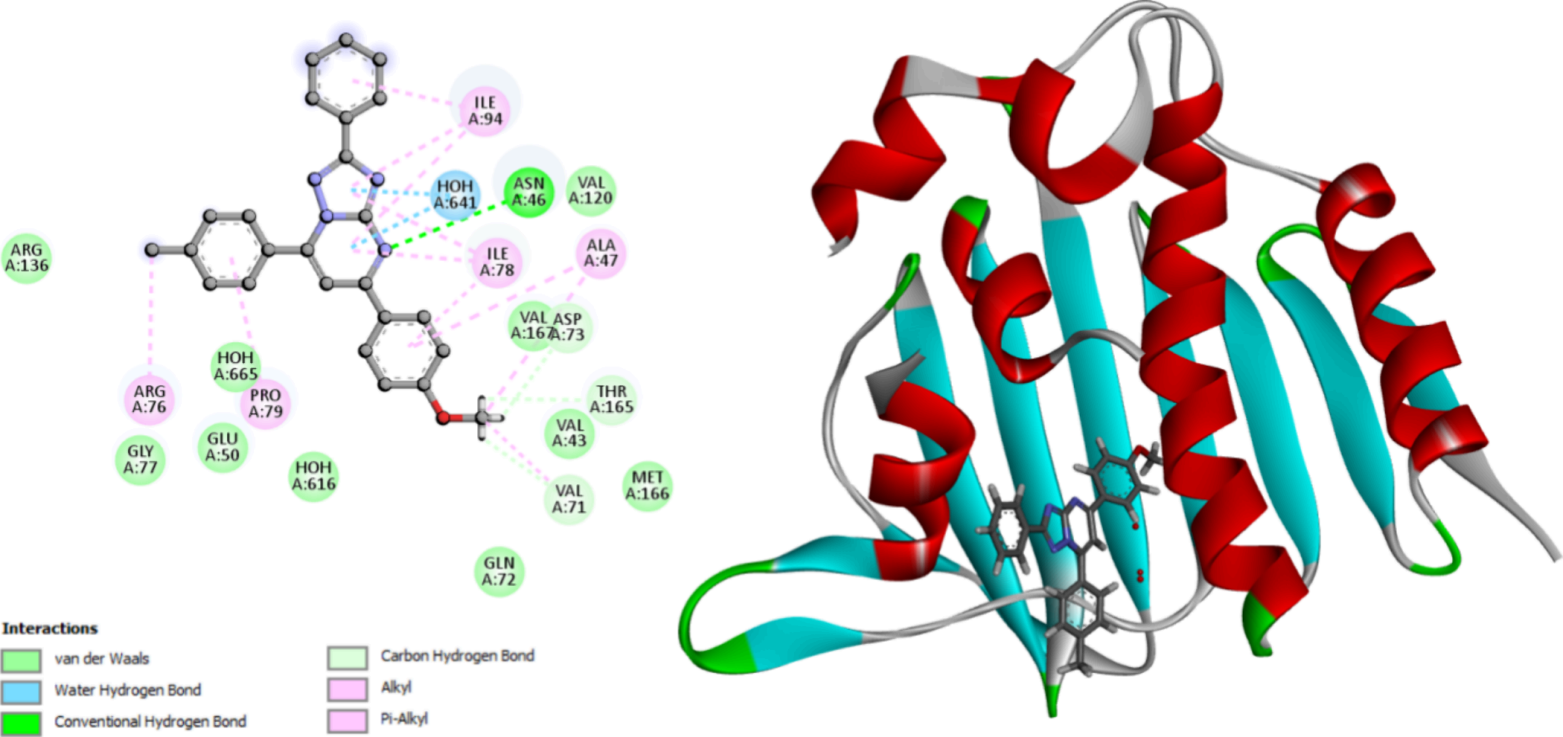
2D diagram representation (left side) and 3D diagram representation (right side) of compound **9o** docked into *E. coli* DNA gyrase B active site showing his binding interactions with the amino acids binding site.

The molecular docking analysis revealed that compounds **9n** and **9o** exhibited a favorable fit within the binding site of *E. coli* DNA gyrase B. The docking energy scores for these compounds were −11.42 and −11.28 kcal/mol, respectively. The two most active molecules, **9n** and **9o**, engage with Asn46 through hydrogen bonding via triazole-pyrimidine N4. The substituted triazolopyrimidine functionals form interactions with amino acids lining the pocket through π-alkyl, alkyl, or van der Waals interactions. These interactions are illustrated in Figures 5 and 6 and summarized in Table 5.

#### 2.3.2 *E. coli* DNA Dihydrofolate Reductase (ecDHFR)

**Figure 7:**
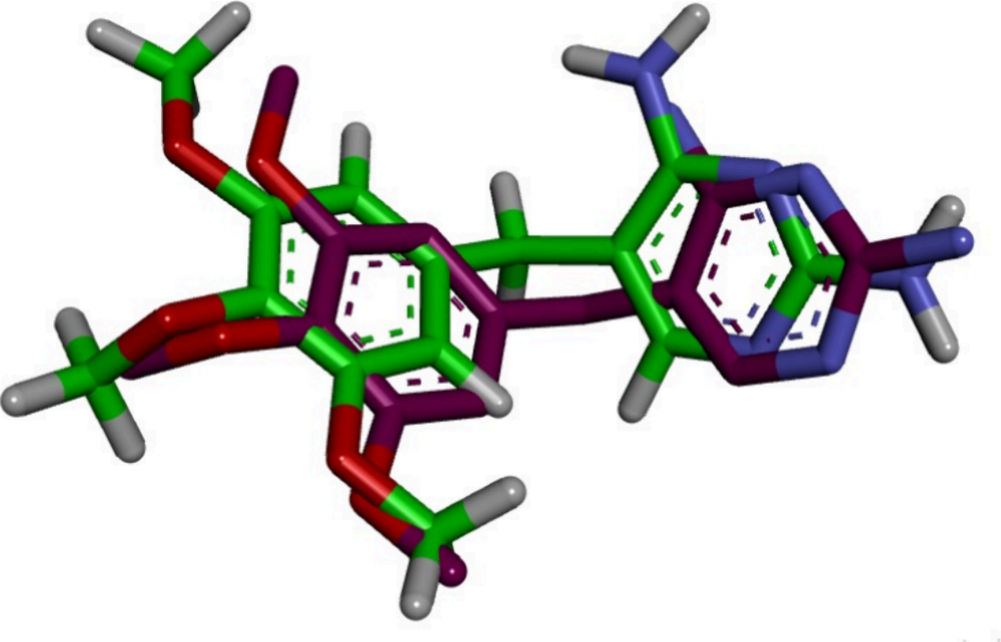
Validation of the accuracy of docking process for ecDHFR. The docked ligand inhibitor (purple) and native ligand inhibitor (green) demonstrated an RMSD of 0.91 Å.

Docking of ecDHFR setup was first validated by performing self-docking of the cocrystallized ligand, TMP, in the active site of ecDHFR (PDB ID: 6R7G).^47^ The self-docking validation replicated the cocrystallized thiazole, demonstrating that the docking methodology is appropriate for the targeted docking investigation. The small RMSD of 0.91 Å demonstrates this between the experimental cocrystallized inhibitor pose and the docked pose and the docking pose’s ability to mimic all essential interactions achieved by the cocrystallized ligands in the active site, Figure 7.

**Figure 8:**
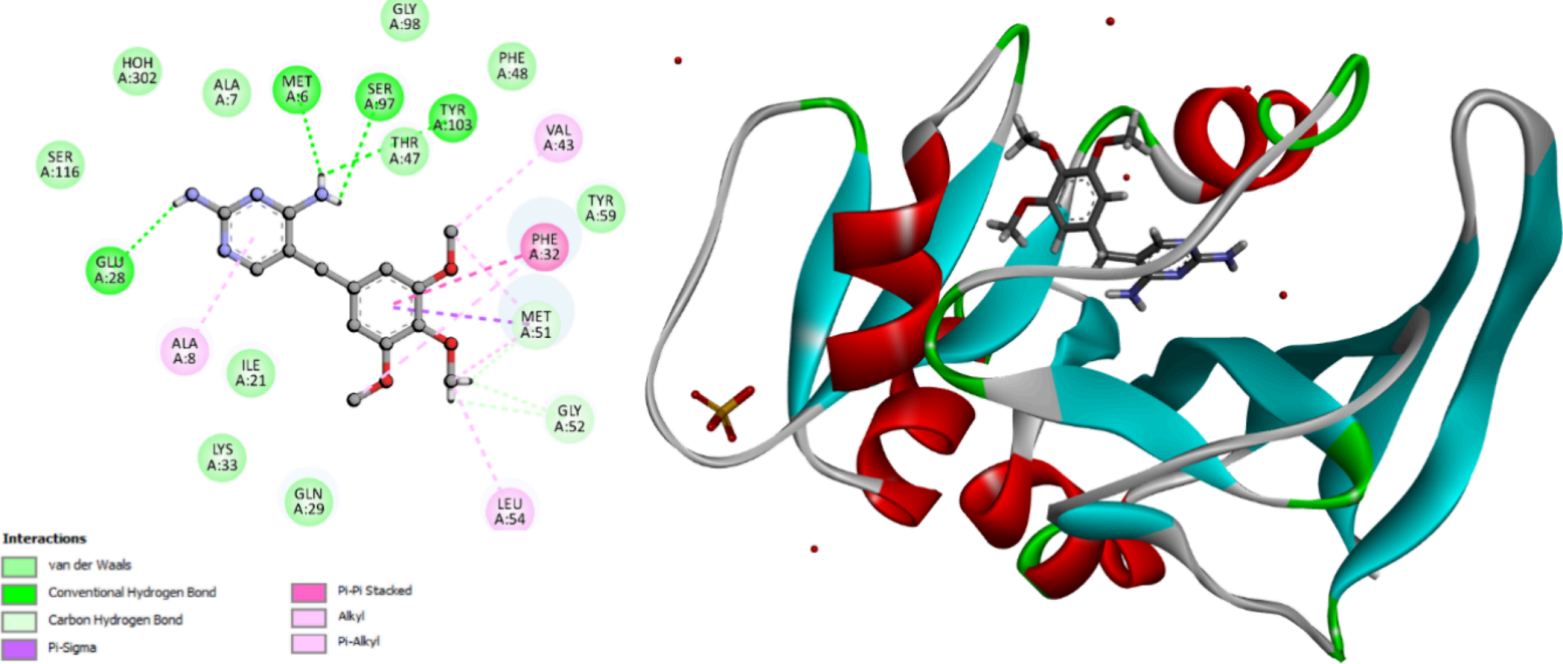
2D diagram representation (left side) and 3D diagram representation (right side) of Trimethoprim docked into ecDHFR active site showing his binding interactions with the amino acids binding site.

The docking energy score (S) was −12.07 kcal/mol. Glu28, Met6, Ser97, and Tyr103 form hydrogen bonds with the amino groups of the 2,4-diaminopyrimidine function. Glu28 interacts with the pyrimidine ring via the μ-anion interaction. The remaining amino acids (Ala7, Ala8, Phe32, Leu64, Gly52, Met51, Val43, Met6) interact with the methoxyphenyl or pyrimidine rings through μ–μ or van der Waals interactions, Figure 8 and Table 6.

**Table 6:** Binding Scores, Amino Acid Interactions, and Bond Lengths of the Selected Compounds within the Active Site of ecDHFR

		Interacting residues		
			Hydrophobic interaction	
Compound	Binding score (S value) kcal/mol	H-bonding	π–π, π–σ, π–alkyl interactions	van der Waals interactions
**9n**	–12.37	Tyr59,	Ala7, Ala8, Leu54, Phe32, Met51, Phe48, Ile15, Tyr103,	Ala53, Gly52, Glyn29, Glu28, Ly26, Ala25, Ser24, Trp23, Ile21, Ser97, Val43, Thr47, Gly99, Gly98,
**9o**	–12.37	Tyr59	Ala7, Ala8, Leu54, Phe32, Met51, Phe48, Ile15, Tyr103,	Ala53, Gly52, Glyn29, Glu28, Ly26, Ala25, Ser24, Trp23, Ile21, Met6, Ser97, Val43, Thr47
**TMP**	–12.07	Glu28, Met6, Ser97, Tyr103	Ala8, Leu54, Val43, Phe32, Met51	Ala7, Leu64, Gly52, Met51, Met6, Glu28, Tyr59, Phe48, Thr47, Thr116, Ile21, Lys33, Gln29,

The molecular docking studies revealed that compounds **9n** and **9o** orient and fit into the ecDHFR binding site similarly, with docking energy scores of −12.37 kcal/mol for both compounds. Compounds **9n** and **9o** form hydrogen bonds with Tyr59 via methoxy oxygen. The substituted triazolopyrimidine functionals interact with the key amino acids lining the pocket, including Ala7, Ala8, Leu54, Phe32, Met51, Phe48, Ile15, Tyr103, Ala53, Gly52, Glyn29, Glu28, Ly26, Ala25, Ser24, Trp23, Ile21, Met6, Ser97, Val43, Thr47. Interact using π–π, π–σ, and π-alkyl interactions. Phe48, Phe32, Lys9, Ile15, Tyr103, Ala7, Al8, Ala25, Lys26, Met51, Gly52, Ala53, Leu54, Tyr59 interact with the different phenyl or triazolopyrimidine rings by μ–μ, π–σ, π-alkyl, or van der Waals interactions, Figures 9 and 10.

**Figure 9:**
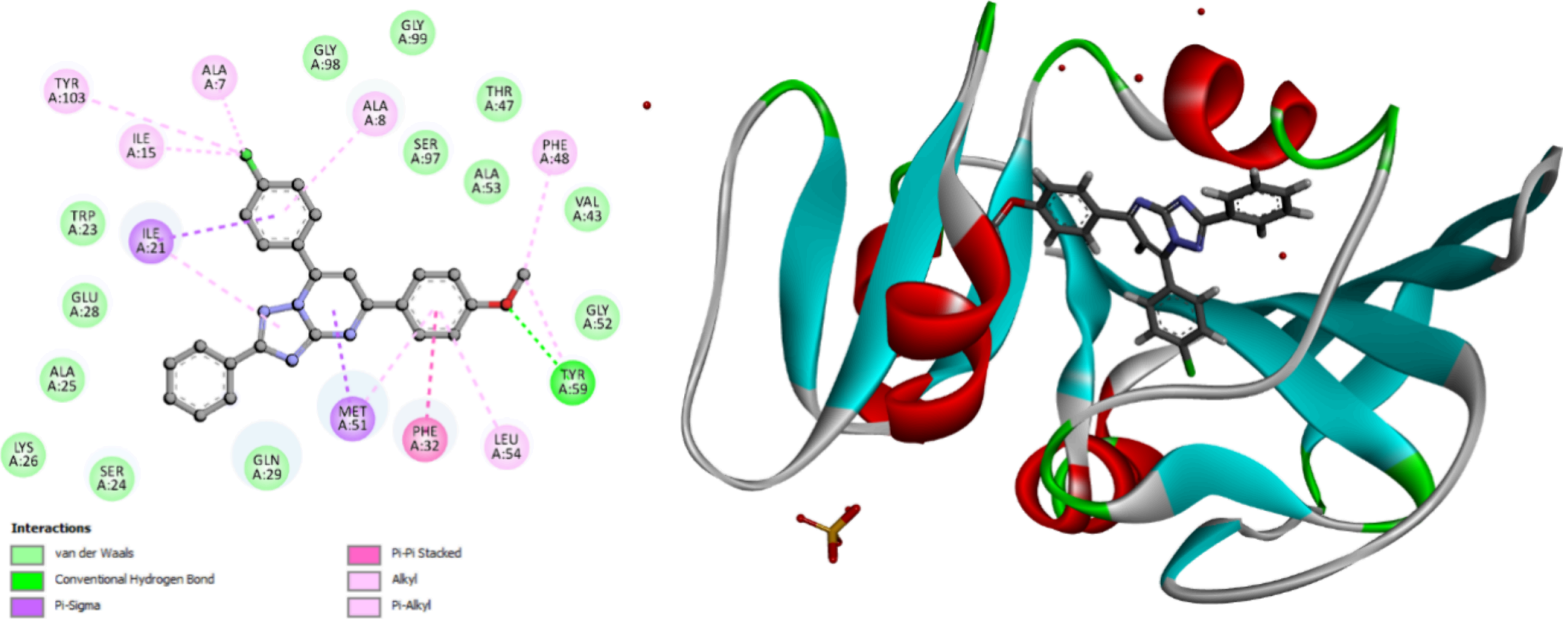
2D diagram representation (left side) and 3D diagram representation (right side) of **9n** docked into ecDHFR active site showing his binding interactions with the amino acids binding site.

**Figure 10:**
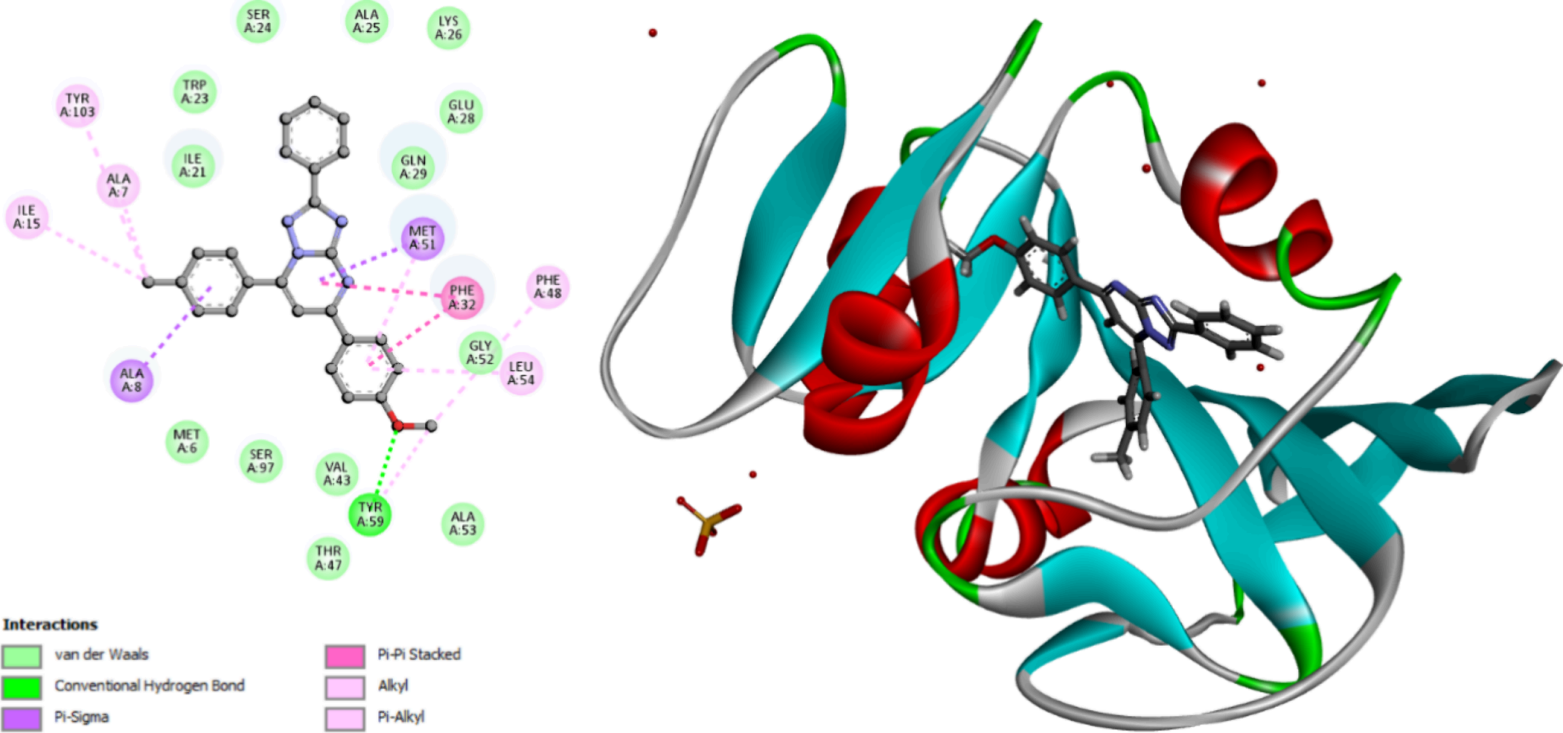
2D diagram representation (left side) and 3D diagram representation (right side) of **9o** docked into ecDHFR active site showing his binding interactions with the amino acids binding site.

### 2.4 Structure–Activity Relationship (SAR) Analysis


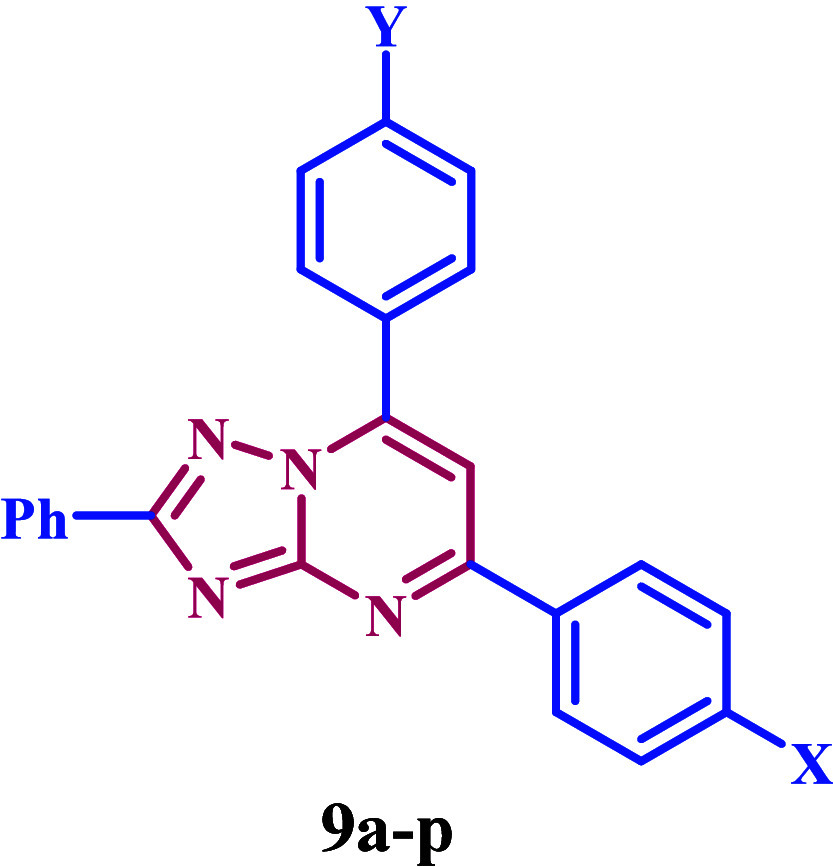

1. The 1,2,4-triazolo[1,5-*a*]pyrimidine framework is crucial for efficacy. The N4 of the triazolo-pyrimidine moiety forms a hydrogen bond with the Asn46 amino acid residue, enhancing the binding to the receptor site of bacterial DNA gyrase. The triazolopyrimidine ring engages with several amino acids lining the pocket site of the DHFR enzyme by μ–μ, π–σ, π-alkyl, or van der Waals interactions.
2. The *para*-position of the phenyl group at position 5 of the 1,2,4-triazolopyrimidine scaffold (X) is crucial for antibacterial efficacy and activity against bacterial DNA gyrase and DHFR, with the reactivity rank as X = OMe > Me > Cl > H. The oxygen atom in the methoxy group participates in hydrogen bonding with Tyr59 in the DHFR receptor site. The methoxy group can engage in interactions with the amino acids that delineate the pocket via π-alkyl, alkyl, or van der Waals forces in bacterial DNA gyrase.
3. The substitution pattern of the para-position of the phenyl group at position 7 (Y) may influence the activity of **9a**–**p**, where the order of reactivity as Y = Me > Cl > OMe. This substitution may improve binding to receptor sites by interactions with amino acids that lining the pocket through π-alkyl, alkyl, or van der Waals forces.

## 3 Conclusions

We evaluated the antimicrobial efficacy of 16 novel compounds based on the 1,2,4-triazolo[1,5-*a*]pyrimidine scaffold against four bacterial strains and two fungus species. Compounds **9d**, **9n**, **9o**, and **9p** show notable antibacterial efficacy. Compounds **9n** and **9o** demonstrated significant bactericidal activity. Furthermore, compounds **9n** and **9d** have shown significant inhibitory action against bacterial DNA gyrase and DHFR as potential molecular targets. Moreover, the molecular docking analysis revealed that compounds **9n** and **9o** exhibited a favorable fit within the binding site of *E. coli* DNA gyrase and DHFR. Structure modifications are under process by adding more substitutions to the phenyl groups at positions 5 and 7 or replacing the phenyl group at position 2 with electron-donating or electron-withdrawing groups. This is undertaken to develop more potent lead compounds for *in vitro*, *in vivo*, and stability research.

## 4 Experimental Section

### 4.1 Chemistry

**General Details**: See Appendix A.

Synthesis and analytical data of compounds **5**(36) and **8a**–**p**^35^ were reported.

#### 4.1.1 Synthesis of 2-(5-Cyano-6-oxo-4-phenyl-1,6-dihydro-pyrimidin-2-ylsulfanyl)-N-(4-phenyl-thiazol-2-yl)acetamide Derivatives (9a-p)

A mixture of 3-phenyl-1,2,4-triazole-5-amine (**5)** (0.30 g, 1.2 mmol) and benzylidene-acetophenone derivatives **(8a-p)** (1.2 mmol) was heated at 210 °C in a solvent-free condition for 1 h. The reaction mixture was cooled and crystallized from ethanol 95%.

##### 4.1.1.1 2,5,7-Triphenyl-1,2,4-triazolo[1,5-*a*]pyrimidine (9a)

Yield, 66%; m.p., 210–212 °C; ^1^H NMR (400 MHz, CDCl_3_) δ 8.33–8.23 (m, 2H, Ar–H), 8.18–8.08 (m, 4H, Ar–H), 7.58–7.50 (m, 2H, Ar–H), 7.46 (s, 1H, Ar–H), 7.44–7.32 (m, 5H, Ar–H), 6.95 (d, J = 8.5 Hz, 2H, Ar–H).13C NMR (101 MHz, CDCl3) δ 165.78, 162.44, 160.68, 157.25 (C = N carbons), 146.05, 137.94, 130.78, 130.59, 130.48, 129.37, 129.22, 128.77, 128.69, 128.66, 127.52, 114.44 (Ar carbons), 105.44 (triazolopyrimidine C_6_). LC-MS (*m*/*z*) calcd for C_23_H_16_N_4_; 348.14, Found: 349.20 [M + H]+. Anal. Calcd for C_23_H_16_N_4_: C, 79.29; H, 4.63; N, 16.08. Found: C, 79.13; H, 4.78; N, 16.11

##### 4.1.1.2 7-(4-Chlorophenyl)-2,5-diphenyl-1,2,4-triazolo[1,5-*a*]pyrimidine (9b)

Yield, 69%; m.p., 214–216 °C; ^1^H NMR (400 MHz, CDCl_3_) δ 8.35–8.28 (m, 2H, Ar–H), 8.16 (td, *J* = 7.9, 7.1, 2.8 Hz, 4H, Ar–H), 7.59–7.54 (m, 2H, Ar–H), 7.50 (s, 1H, Ar–H), 7.41 (dd, *J* = 5.2, 2.0 Hz, 4H, Ar–H), 6.98–6.93 (m, 2H, Ar–H). LC-MS (*m*/*z*) calcd for C_23_H_15_ClN_4_; 382.10, Found: 382.87 [M + H]^+^. Anal. Calcd for C_23_H_15_ClN_4_: C, 72.16; H, 3.95; N, 14.63. Found: C, 72.25; H, 3.82; N, 14.70

##### 4.1.1.3 2,5-Diphenyl-7-*p*-tolyl-1,2,4-triazolo[1,5-*a*]pyrimidine (9c)

Yield, 68%; m.p., 218–220 °C; ^1^H NMR (400 MHz, CDCl_3_) δ 8.48–8.37 (m, 2H, Ar–H), 8.31–8.16 (m, 2H, Ar–H), 8.11 (d, *J* = 7.9 Hz, 2H, Ar–H), 7.64–7.43 (m, 7H, Ar–H), 7.37 (d, *J* = 7.9 Hz, 2H, Ar–H), 2.46 (s, 3H, CH_3_). LC-MS (*m*/*z*) calcd for C_24_H_18_N_4_; 362.15, Found: 363.10 [M + H]^+^. Anal. Calcd for C_24_H_18_N_4_: C, 79.54; H, 5.01; N, 15.46. Found: C, 79.65; H, 4.89; N, 15.35.

##### 4.1.1.4 7-(4-Methoxyphenyl)-2,5-diphenyl-1,2,4-triazolo[1,5-*a*]pyrimidine (9d)

Yield, 72%; m.p., 228–230 °C; ^1^H NMR (400 MHz, CDCl_3_) δ 8.24 (dd, *J* = 6.7, 3.0 Hz, 2H), 8.12 (d, *J* = 8.6 Hz, 2H), 8.02 (d, *J* = 8.3 Hz, 2H), 7.38–7.29 (m, 7H), 6.97 (d, *J* = 8.6 Hz, 2H), 3.80 (s, 3H). LC-MS (*m*/*z*) calcd for C_24_H_18_N_4_O; 378.15, Found: 379.00 [M + H]^+^. Anal. Calcd for C_24_H_18_N_4_O: C, 76.17; H, 4.79; N, 14.81. Found: C, 76.07; H, 4.86; N, 14.95.

##### 4.1.1.5 5-(4-Chlorophenyl)-2,7-diphenyl-1,2,4-triazolo[1,5-*a*]pyrimidine (9e)

Yield, 76%; m.p., 260–262 °C; ^1^H NMR (400 MHz, CDCl_3_) δ 8.30–8.24 (m, 2H), 8.16–8.06 (m, 4H), 7.55 (d, *J* = 5.7 Hz, 2H), 7.47 (s, 1H), 7.39 (d, *J* = 6.8 Hz, 6H). LC-MS (*m*/*z*) calcd for C_23_H_15_ClN_4_; 382.10, Found: 383.00 [M + H]^+^. Anal. Calcd for C_23_H_15_ClN_4_: C, 72.16; H, 3.95; N, 14.63. Found: C, 72.25; H, 4.08; N, 14.70.

##### 4.1.1.6 5,7-Bis(4-Chlorophenyl)-2-phenyl-1,2,4-triazolo[1,5-*a*]pyrimidine (9f)

Yield, 70%; m.p., 202–204 °C; ^1^H NMR (400 MHz, CDCl_3_) δ 8.30 (dd, J = 6.7, 3.0 Hz, 2H, Ar–H), 8.14 (d, J = 8.2 Hz, 4H, Ar–H), 7.56 (d, J = 8.1 Hz, 2H, Ar–H), 7.50 (s, 1H, Ar–H), 7.47–7.37 (m, 5H, Ar–H). LC-MS (*m*/*z*) calcd for C_23_H_14_Cl_2_N_4_; 416.06, Found: 417.20 [M + H]^+^. Anal. Calcd for C_23_H_14_Cl_2_N_4_: C, 66.20; H, 3.38; N, 13.43. Found: C, 66.32; H, 3.45; N, 13.35.

##### 4.1.1.7 5-(4-Chlorophenyl)-2-phenyl-7-*p*-tolyl-1,2,4-triazolo[1,5-*a*]pyrimidine (9g)

Yield, 74%; m.p., 248–250 °C; ^1^H NMR (400 MHz, CDCl_3_) δ 8.26 (dd, *J* = 6.7, 3.0 Hz, 2H, Ar–H), 8.04 (dd, *J* = 15.4, 8.1 Hz, 4H, Ar–H), 7.35 (td, *J* = 20.0, 18.8, 10.2 Hz, 8H, Ar–H), 2.39 (s, 3H). LC-MS (*m*/*z*) calcd for C_24_H_17_ClN_4_; 396.11, Found: 397.10 [M + H]^+^. Anal. Calcd for C_24_H_17_ClN_4_: C, 72.63; H, 4.32; N, 14.12. Found: C, 72.75; H, 4.25; N, 14.20.

##### 4.1.1.8 5-(4-Chlorophenyl)-7-(4-methoxyphenyl)-2-phenyl-1,2,4-triazolo[1,5-*a*]pyrimidine (9h)

Yield, 73%; m.p., 236–238 °C; ^1^H NMR (400 MHz, DMSO) δ 8.50–8.40 (m, 4H, Ar–H), 8.29–8.22 (m, 2H, Ar–H), 8.11 (s, 2H, Ar–H), 7.67–7.64 (m, 2H, Ar–H), 7.57 (dq, *J* = 4.8, 3.4, 2.4 Hz, 2H, Ar–H), 7.24 (d, *J* = 9.0 Hz, 2H, Ar–H), 3.92 (s, 3H, OCH_3_). LC-MS (*m*/*z*) calcd for C_24_H_17_ClN_4_O; 412.11, Found: 413.20 [M + H]^+^. Anal. Calcd for C_24_H_17_ClN_4_O: C, 69.82; H, 4.15; N, 13.57. Found: C, 69.95; H, 4.06; N, 13.65.

##### 4.1.1.9 2,7-Diphenyl-5-*p*-tolyl-1,2,4-triazolo[1,5-*a*]pyrimidine (9i)

Yield, 70%; m.p., 238–240 °C^1^H NMR (400 MHz, DMSO) δ 8.36–8.30 (m, 2H, Ar–H), 8.22 (t, *J* = 7.4 Hz, 4H, Ar–H), 7.99 (s, 1H, Ar–H), 7.67 (d, *J* = 5.4 Hz, 3H, Ar–H), 7.53 (d, *J* = 5.9 Hz, 3H, Ar–H), 7.32 (d, *J* = 7.9 Hz, 2H, Ar–H), 2.35 (s, 3H, CH_3_). LC-MS (*m*/*z*) calcd for C_24_H_18_N_4_; 362.15, Found: 363.10 [M + H]^+^. Anal. Calcd for C_24_H_18_N_4_: C,79.54; H, 5.01; N, 15.46. Found: C,79.48; H, 5.11; N, 15.35.

##### 4.1.1.10 7-(4-Chlorophenyl)-2-phenyl-5-*p*-tolyl-1,2,4-triazolo[1,5-*a*]-pyrimidine (9j)

Yield, 72%; m.p., 240–242 °C. ^1^H NMR (400 MHz, CDCl_3_) δ 8.36–8.30 (m, 2H, Ar–H), 8.15 (d, *J* = 8.2 Hz, 2H, Ar–H), 8.06 (d, *J* = 7.8 Hz, 2H, Ar–H), 7.57 (d, *J* = 8.1 Hz, 2H, Ar–H), 7.47 (s, 4H, Ar–H), 7.23 (d, *J* = 7.8 Hz, 2H, Ar–H), 2.37 (s, 3H, CH_3_). ^13^C NMR (101 MHz, CDCl_3_) δ 165.74, 160.77, 157.13 (C = N carbons), 145.86, 141.88, 137.86, 133.27, 132.12, 130.79, 130.56, 130.45, 129.67, 129.47, 129.08, 128.81, 128.62, 128.57, 128.31, 127.47, 127.44, 127.21(Ar carbons), 105.55 (triazolopyrimidine C_6_), 21.47 (CH_3_ carbons). LC-MS (*m*/*z*) calcd for C_24_H_17_ClN_4_; 396.11, Found: 397.10 [M + H]^+^. Anal. Calcd for C_24_H_17_ClN_4_: C,72.63; H, 4.32; N, 14.12. Found: C, 72.74; H, 4.25; N, 14.20.

##### 4.1.1.11 2-Phenyl-5,7-di-*p*-tolyl-1,2,4-triazolo[1,5-*a*]pyrimidine (9k)

Yield, 68%; m.p., 224–226 °C. ^1^H NMR (400 MHz, CDCl_3_) δ 8.39–8.30 (m, 2H, Ar–H), 8.18–8.06 (m, 4H, Ar–H), 7.56 (s, 1H, Ar–H), 7.48–7.37 (m, 4H, Ar–H), 7.37 (s, 1H, Ar–H), 7.28 (d, *J* = 8.0 Hz, 2H, Ar–H), 2.44 (s, 3H, CH_3_), 2.38 (s, 3H, CH_3_). ^13^C NMR (101 MHz, CDCl_3_) δ 147.56, 142.44, 141.88 (C = N carbons), 133.72, 130.58, 129.80, 129.63, 129.43, 128.65, 127.70, 127.63, 127.44 (Ar carbons), 105.70 (triazolopyrimidine C_6_), 21.70, 21.53 (CH_3_ carbons). LC-MS (*m*/*z*) calcd for C_25_H_20_N_4_; 376.17, Found: 377.00 [M + H]^+^. Anal. Calcd for C_25_H_20_N_4_: C,79.76; H, 5.35; N, 14.88. Found: C, 79.68; H, 5.43; N, 14.75.

##### 4.1.1.12 7-(4-Methoxyphenyl)-2-phenyl-5-*p*-tolyl-1,2,4-triazolo[1,5-*a*]pyrimidine (9l)

Yield, 69%; m.p., 222–224 °C. ^1^H NMR (400 MHz, DMSO) δ 8.50–8.42 (m, 2H, Ar–H), 8.35–8.28 (m, 2H, Ar–H), 8.28–8.20 (m, 2H, Ar–H), 8.05 (s, 1H, Ar–H), 7.58 (s, 1H, Ar–H), 7.59–7.50 (m, 2H, Ar–H), 7.40 (d, *J* = 8.2 Hz, 2H, Ar–H), 7.31–7.20 (m, 2H, Ar–H), 3.92 (s, 3H, OCH_3_), 2.42 (s, 3H, CH_3_). LC-MS (*m*/*z*) calcd for C_25_H_20_N_4_O; 392.16, Found: 393.100 [M + H]^+^. Anal. Calcd for C_25_H_20_N_4_O: C,76.51; H, 5.14; N, 14.28. Found: C, 76.63; H, 5.20; N, 14.17.

##### 4.1.1.13 5-(4-Methoxyphenyl)-2,7-diphenyl-1,2,4-triazolo[1,5-*a*]pyrimidine (9m)

Yield, 70%; m.p., 222–224 °C. ^1^H NMR (400 MHz, DMSO) δ 8.45–8.35 (m, 4H, Ar–H), 8.27–8.20 (m, 3H, Ar–H), 8.11 (s, 2H, Ar–H), 7.81–7.76 (m, 2H, Ar–H), 7.60–7.55 (m, 2H, Ar–H), 7.19–7.12 (m, 2H, Ar–H), 3.87 (d, *J* = 6.0 Hz, 3H, OCH_3_). LC-MS (*m*/*z*) calcd for C_24_H_18_N_4_O; 378.15, Found: 379.130 [M + H]^+^. Anal. Calcd for C_24_H_18_N_4_O: C,76.17; H, 4.79; N, 14.81. Found: C, 76.30; H, 4.86; N, 14.75.

##### 4.1.1.14 7-(4-Chlorophenyl)-5-(4-methoxyphenyl)-2-phenyl-1,2,4-triazolo[1,5-*a*]pyrimidine (9n)

Yield, 70%; m.p., 226–228 °C. ^1^H NMR (400 MHz, DMSO) δ 8.41–8.35 (m, 2H, Ar–H), 8.35–8.29 (m, 2H, Ar–H), 8.23–8.11 (m, 2H, Ar–H), 8.01 (s, 1H, Ar–H), 7.77–7.67 (m, 2H, Ar–H), 7.60–7.44 (m, 3H, Ar–H), 7.12–7.04 (m, 2H, Ar–H), 3.84 (s, 3H, OCH_3_). LC-MS (*m*/*z*) calcd for C_24_H_17_ClN_4_O; 412.11, Found: 413.00 [M + H]^+^. Anal. Calcd for C_24_H_17_ClN_4_O: C, 69.82; H, 4.15; N, 13.57. Found: C, 69.94; H, 4.23; N, 13.68.

##### 4.1.1.15 5-(4-Methoxyphenyl)-2-phenyl-7-*p*-tolyl-1,2,4-triazolo[1,5-*a*]pyrimidine (9o)

Yield, 64%; m.p., 176–168 °C. ^1^H NMR (400 MHz, CDCl_3_) δ 8.33–8.23 (m, 2H, Ar–H), 8.09 (d, *J* = 8.4 Hz, 2H, Ar–H), 8.01 (d, *J* = 7.8 Hz, 2H, Ar–H), 7.43–7.33 (m, 4H, Ar–H), 7.30 (d, *J* = 7.9 Hz, 2H, Ar–H), 6.87 (d, *J* = 8.3 Hz, 2H, Ar–H), 3.74 (s, 3H, OCH_3_), 2.38 (s, 3H, CH_3_). LC-MS (*m*/*z*) calcd for C_25_H_20_N_4_O; 392.16, Found: 393.20 [M + H]^+^. Anal. Calcd for C_25_H_20_N_4_O: C,76.51; H, 5.14; N, 14.28. Found: C, 76.38; H, 5.21; N, 14.20.

##### 4.1.1.16 5,7-Bis(4-methoxyphenyl)-2-phenyl-1,2,4-triazolo[1,5-*a*]pyrimidine (9p)

Yield, 68%; m.p., 202–204 °C. ^1^H NMR (400 MHz, CDCl_3_) δ 8.35–8.26 (m, 2H, Ar–H), 8.24–8.14 (m, 2H, Ar–H), 8.18–8.05 (m, 2H, Ar–H), 7.48–7.33 (m, 4H, Ar–H), 7.11–7.01 (m, 2H, Ar–H), 6.98–6.87 (m, 2H, Ar–H), 3.86 (s, 3H, OCH_3_), 3.80 (s, 3H, OCH_3_). LC-MS (*m*/*z*) calcd for C_25_H_20_N_4_O_2_; 408.16, Found: 409.30 [M + H]^+^. Anal. Calcd for C_25_H_20_N_4_O_2_: C,73.51; H, 4.94; N, 13.72. Found: C, 73.63; H, 5.05; N, 13.57.

### 4.2 Biology

#### 4.2.1 Antimicrobial Assay

The antimicrobial activity of the target compounds **(9a-p)** was tested against two Gram-positive bacteria (*S. aureus* and *B. subtilis*), two Gram-negative bacteria (*E. coli* and *P. aeruginosa*), and two fungal strains (*A*. *flavus* and C. *albicans*). The inhibition zones (IZ, mm/ml) and minimal inhibitory concentrations (MIC, nM) were determined using the modified disk diffusion method.^39,40^ The MIC values for selected compounds were determined using dose–response tests. The given values result from at least two studies, each with three replicates per concentration. Appendix A (Supplementary File) provides experimental details.

#### 4.2.2 DNA Gyrase and DHFR Inhibitory Assays

The inhibitory efficacy of derivatives **9d**, **9n**, **9o**, and **9p**, the most potent antibacterial agents, was assessed against *E. coli* DNA gyrase and DHFR.^44^ Inspiralis assay kits measured inhibitory activity against DNA gyrase and DHFR in *E. coli*. IC_50_ values were determined using seven different inhibitor concentrations and then calculated using the GraphPad Prism 6.0 software. We established IC_50_ values for the most important inhibitors using three independent measurements and provided the final results as mean values. Refer to Appendix A for more details.

### 4.3 Docking Experiment

Two enzymes examined were *E. coli* DNA gyrase (PDB ID 6F86) and *E. coli* dihydrofolate reductase B (PDB ID: 7r6g). The cocrystallized structures of the two enzymes were downloaded from the Protein Data Bank,^43,44^ Chem Office tools (ChemDraw ultra 8.0 2D and 3D) were used to prepare the energy-minimized chemical structures of the ligands; Open Babel GUI was used to convert the images of the ligands into their different file formats. The two enzymes were prepared by removing unnecessary cocrystallized ligands and water and adding polar hydrogen and charges using Autodock 4.2 and Discovery Studio 2024. The active sites of the enzymes were taken from the attributes of SPD Sphere of the corresponding enzyme, which were x,y,z = 61.680259, 28.330852, 64.290148, radius= 10.105432 for 6F86; x,y,z = 10.093469, −53.275716, −7.239296, radius = 10.093469, – 53.275716, – 7.239296 for 7RG6.

## Supporting Information Available

^1^H NMR, ^13^C NMR, LCMS spectra, and elemental analysis of compounds **9a**–**p** (DOCX)
