# Supplementary material for: Synthesis and Antimicrobial Evaluation of New 1,2,4-Triazolo[1,5-a]pyrimidine-Based Derivatives as Dual Inhibitors of Bacterial DNA Gyrase and DHFR
Source: ACS Omega. 2024 Nov 11;9(47):47261–73. doi: 10.1021/acsomega.4c08365 (PMC11603275; doi:10.1021/acsomega.4c08365)
Supplement: Supplementary file 1 — ao4c08365_si_001.docx [file ao4c08365_si_001.docx]

**Supplementary Data**

**Synthesis and antimicrobial evaluation of new 1,2,4-triazolo[1,5-a]pyrimidine-based derivatives as dual inhibitors of bacterial DNA gyrase and DHFR**

Lamya H. Al-Wahaibi^1^, Safwat M. Rabea^2^, Mohamed A. Mahmoud^3^, Bahaa G.M. Youssif^3^*, Stefan Bräse^4^*, Salah A. Abdel-Aziz^5,6^

^1^Department of Chemistry, College of Sciences, Princess Nourah bint Abdulrahman University, Riyadh 11671, Saudi Arabia; ^2^Medicinal Chemistry Department, Faculty of Pharmacy, Minia University, Minia 61519, Egypt; ^3^Department of Pharmaceutical Organic Chemistry, Faculty of Pharmacy, Assiut University, Assiut 71526, Egypt; ^4^Institute of Biological and Chemical Systems, IBCS-FMS, Karlsruhe Institute of Technology, 76131 Karlsruhe, Germany; ^5^Department of Pharmaceutical Medicinal Chemistry and Drug Design, Faculty of Pharmacy (Boys) Al‐Azhar University Assiut 71524, Egypt; ^6^Department of Pharmaceutical Chemistry, Faculty of Pharmacy, Deraya University, Minia 61519, Egypt.

**To whom correspondence should be addressed:*

**Bahaa G. M. Youssif**, Ph.D. Pharmaceutical Organic Chemistry Department, Faculty of Pharmacy, Assiut University, Assiut 71526, Egypt.

**Tel**.: (002)-01098294419

**E-mail address**: [bgyoussif2@gmail.com](mailto:bgyoussif2@gmail.com)

**S. Bräse**

Institute of Biological and Chemical Systems, IBCS-FMS, Karlsruhe Institute of Technology, 76131 Karlsruhe, Germany. E-mail: [braese@kit.edu](mailto:braese@kit.edu)

**Figure S1**: ^1^H NMR spectrum (400 MHz, DMSO-*d*_6_) of compound **9a**

**
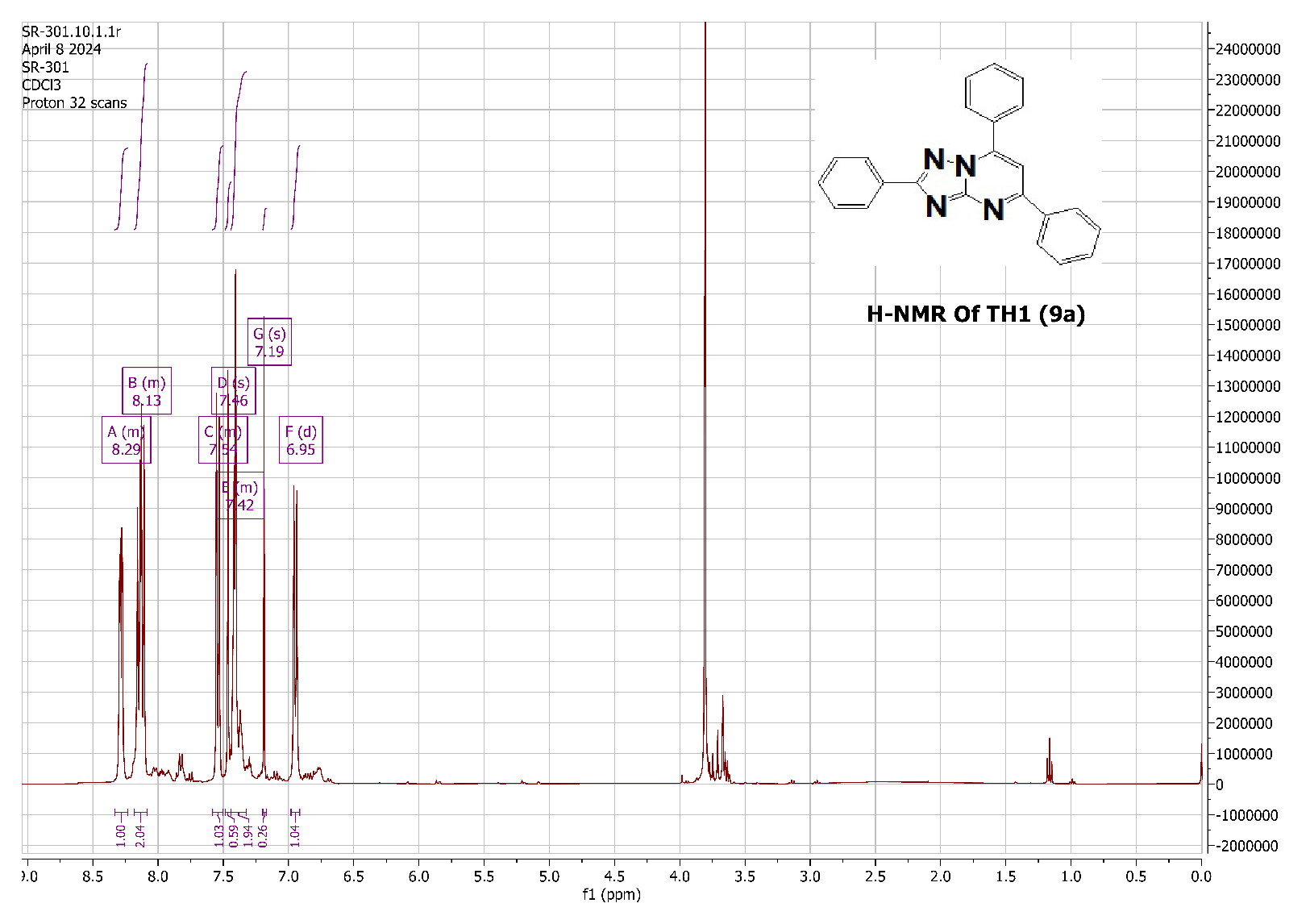
**

**Figure S2**: Expanded ^1^H NMR spectrum (400 MHz, DMSO-*d*_6_) of compound **9a**

**
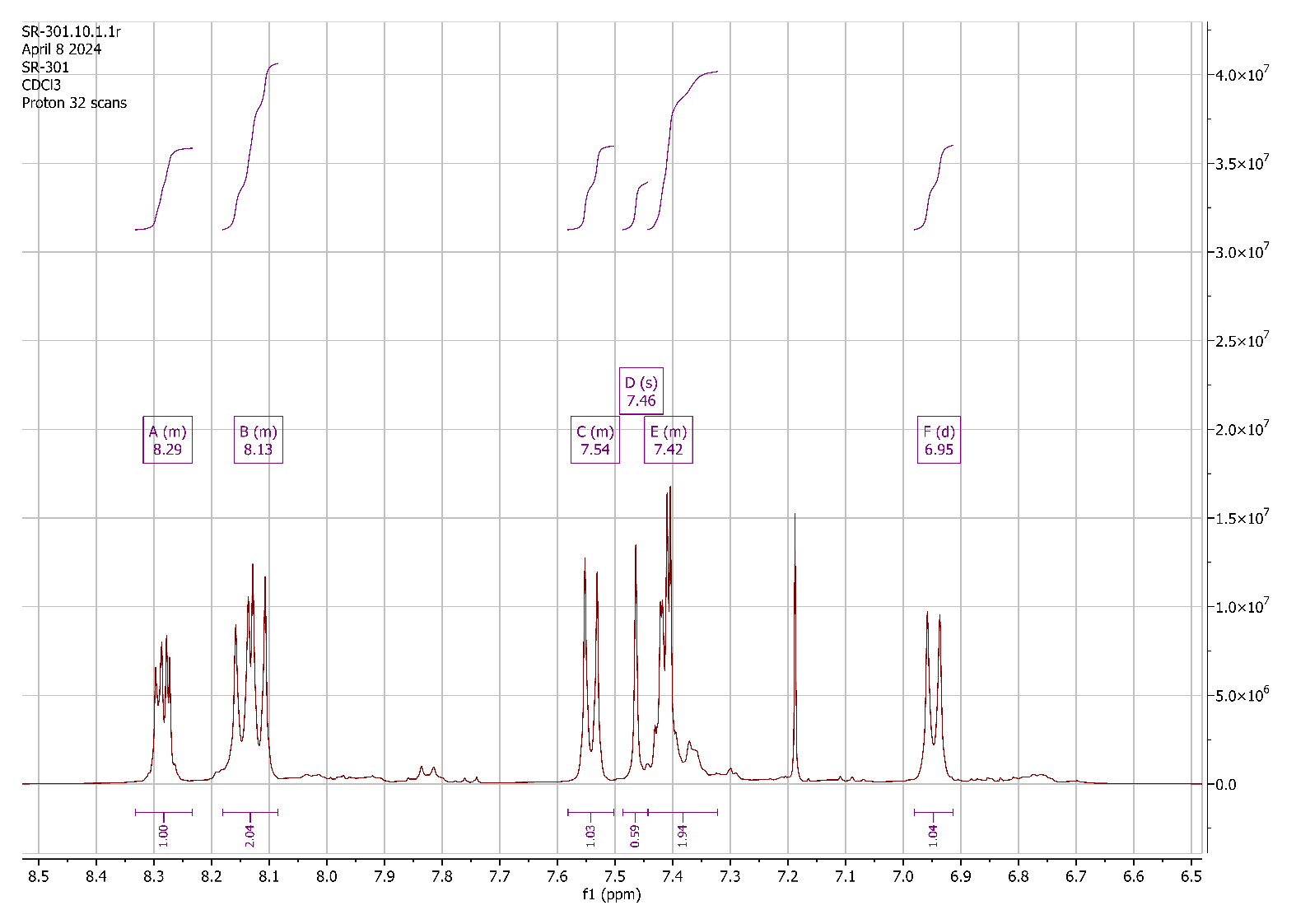
**

**
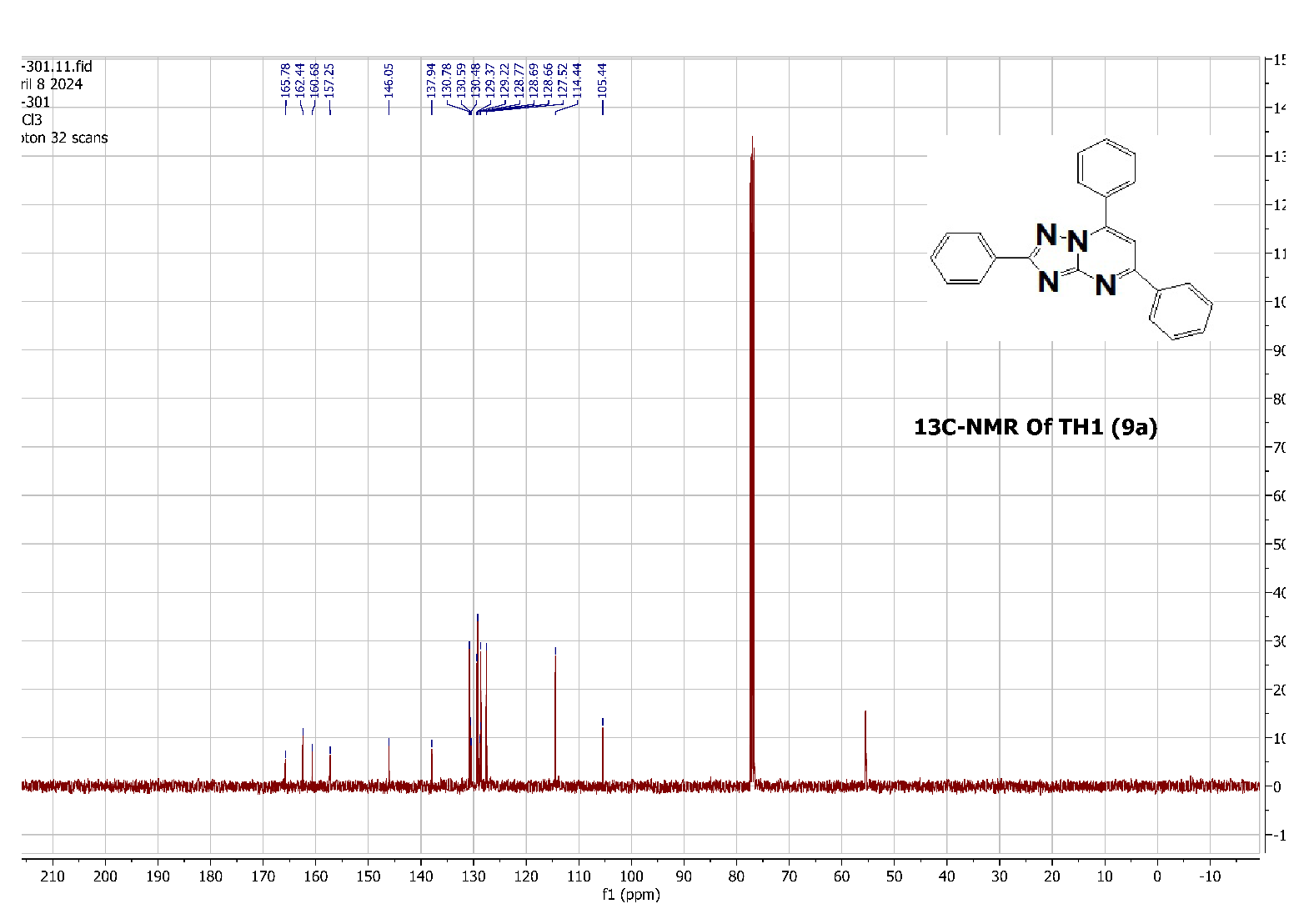
Figure S3**: ^13^CNMR spectrum (100 MHz, DMSO-*d*_6_) of compound **9a**

**
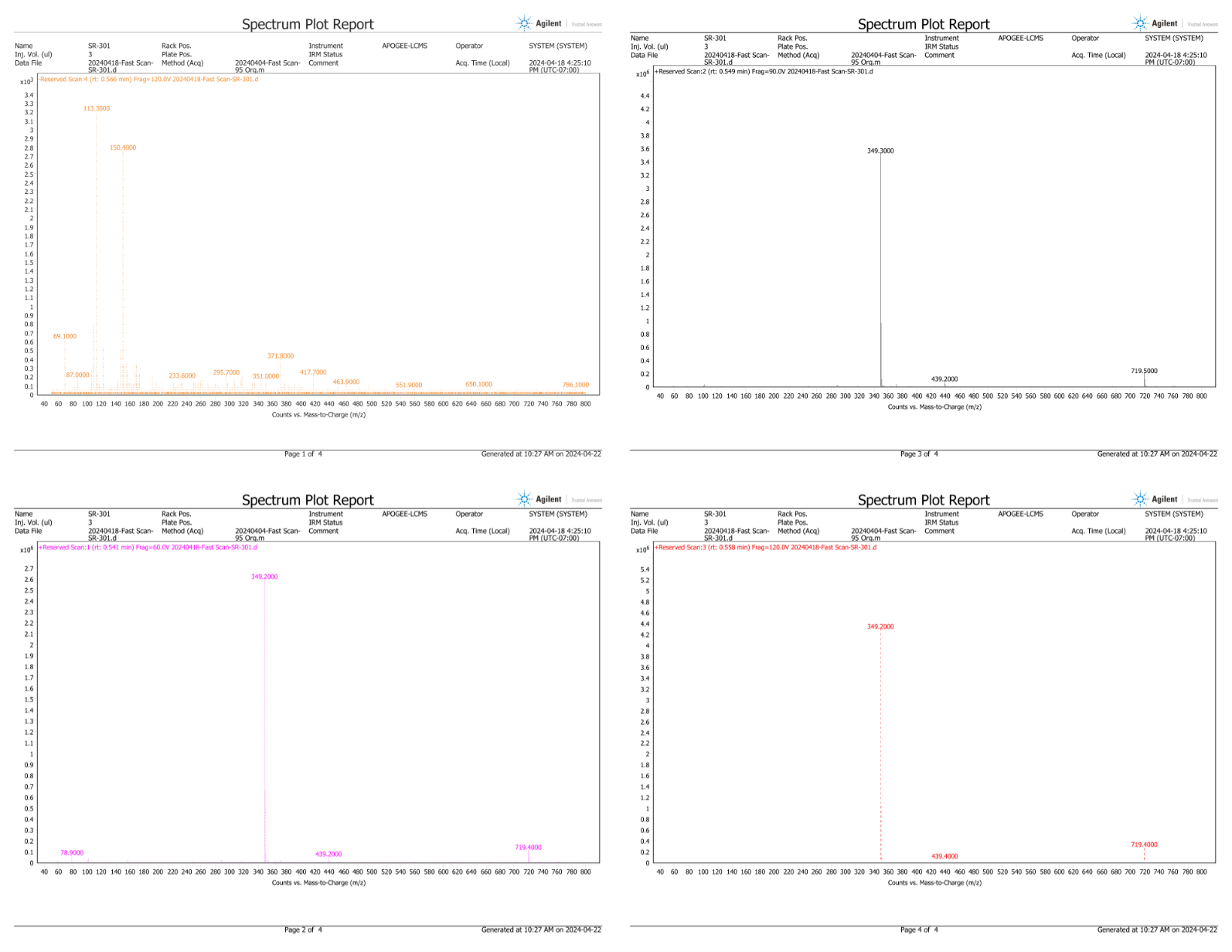
Figure S4**: LC-MS collated spectrum of compound **9a**

**
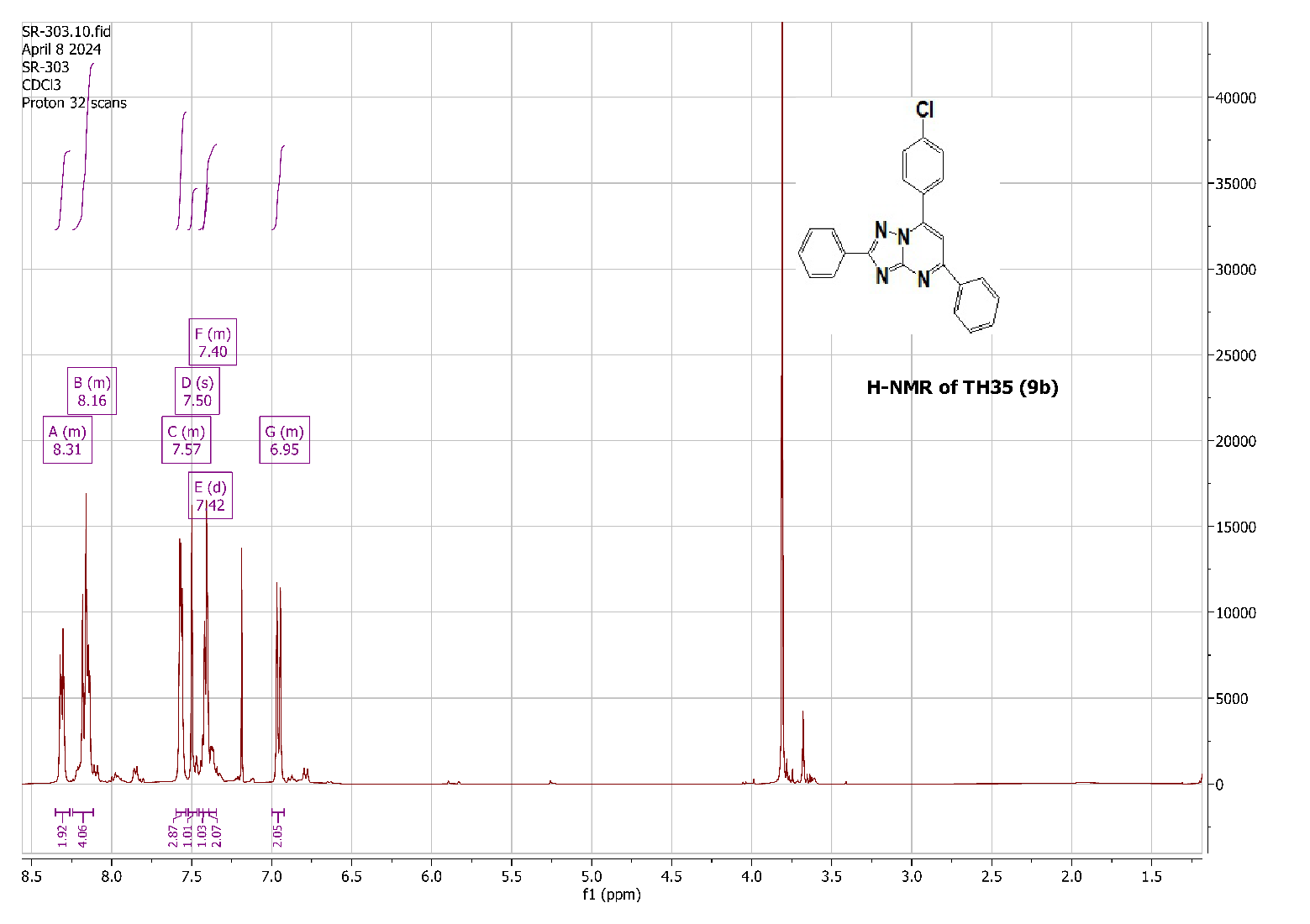
Figure S5**: ^1^H NMR spectrum (400 MHz, DMSO-*d*_6_) of compound **9b**

**Figure S6**: Expanded ^1^H NMR spectrum (400 MHz, DMSO-*d*_6_) of compound **9b**

**
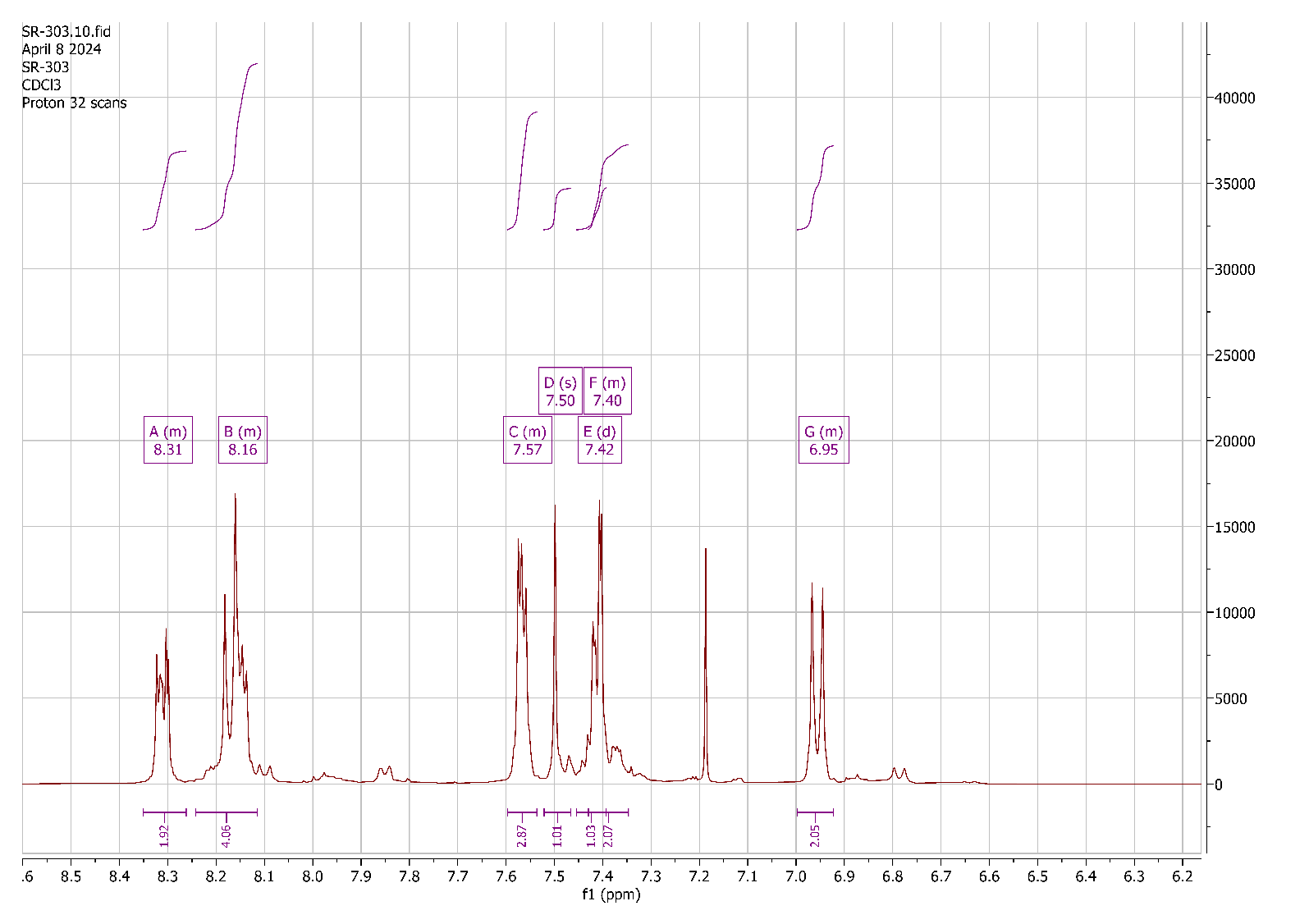
**

**
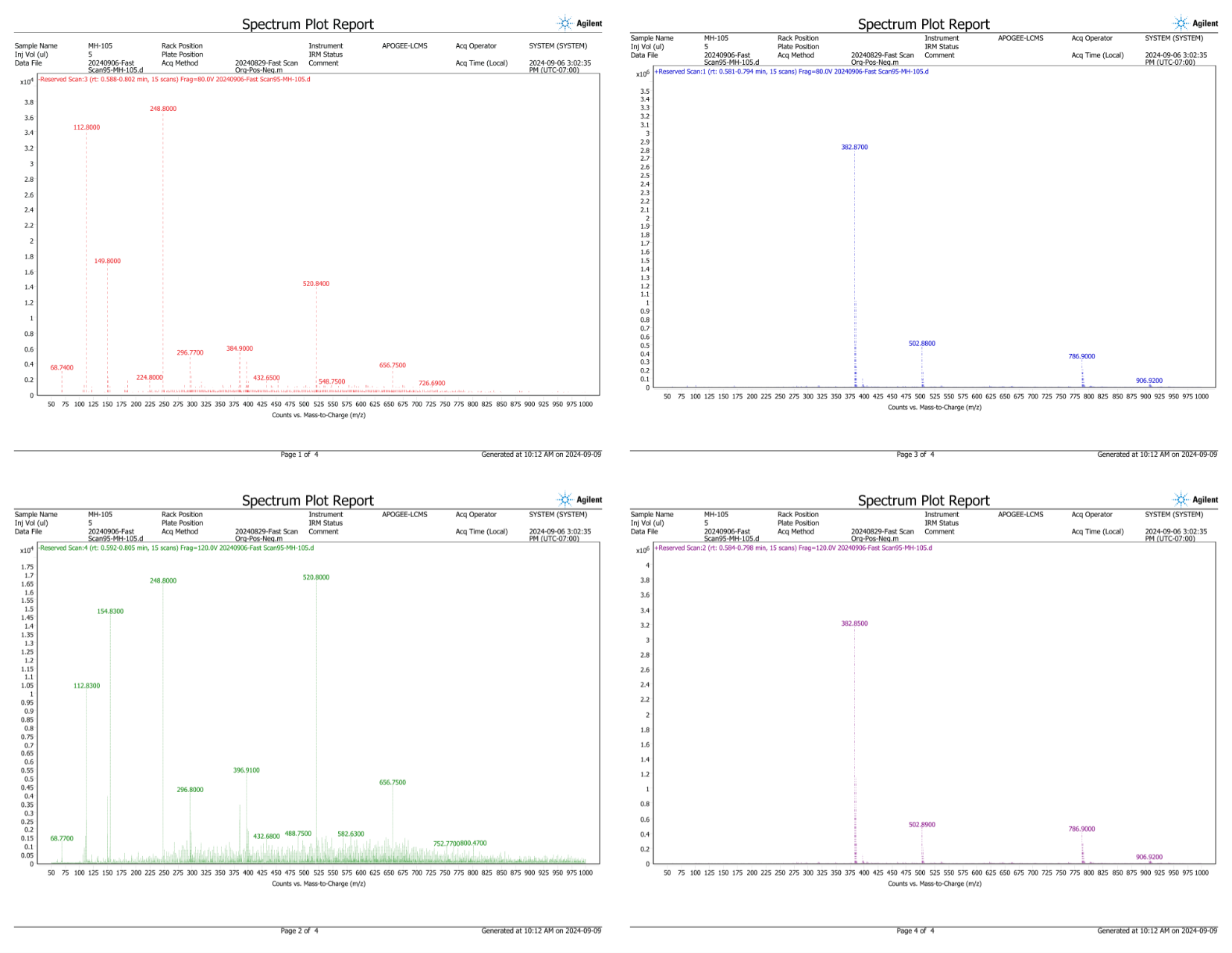
Figure S7**: LC-MS collated spectrum of compound **9b**

**Figure S8**: ^1^H NMR spectrum (400 MHz, DMSO-*d*_6_) of compound **9c**

**
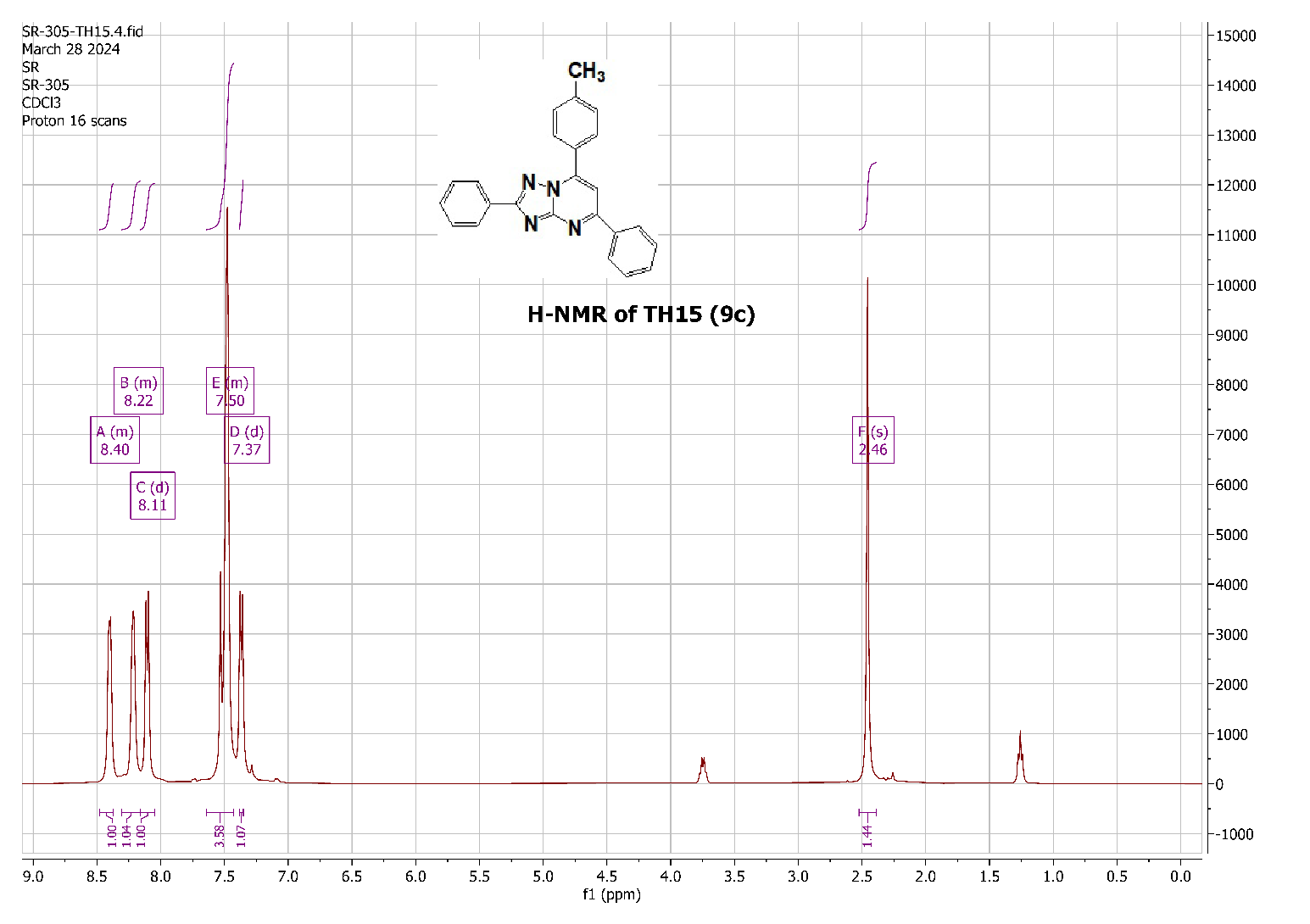
**

**Figure S9**: Expanded ^1^H NMR spectrum (400 MHz, DMSO-*d*_6_) of compound **9c**

**
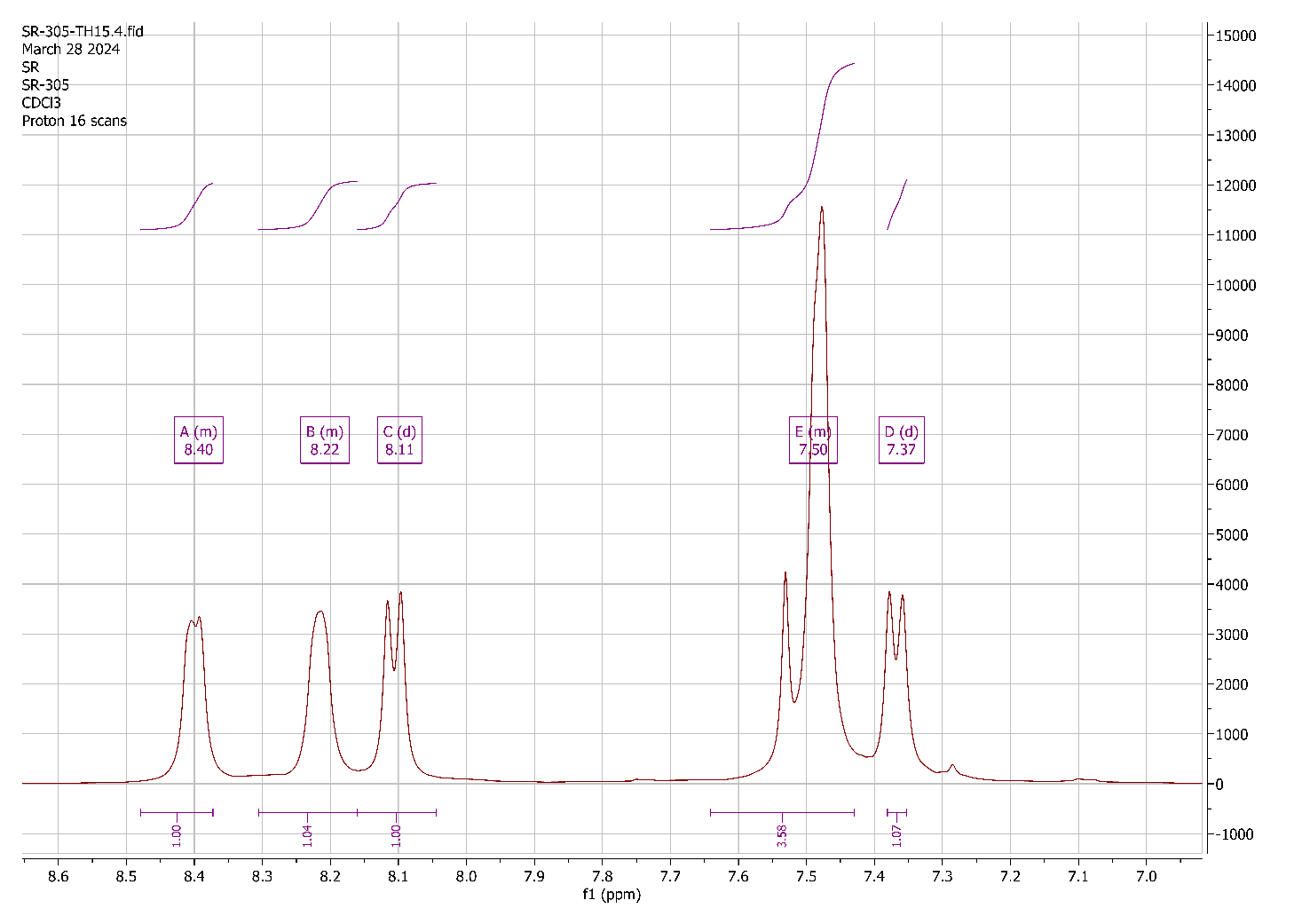
**

**Figure S10**: LC-MS collated spectrum of compound **9c**

**
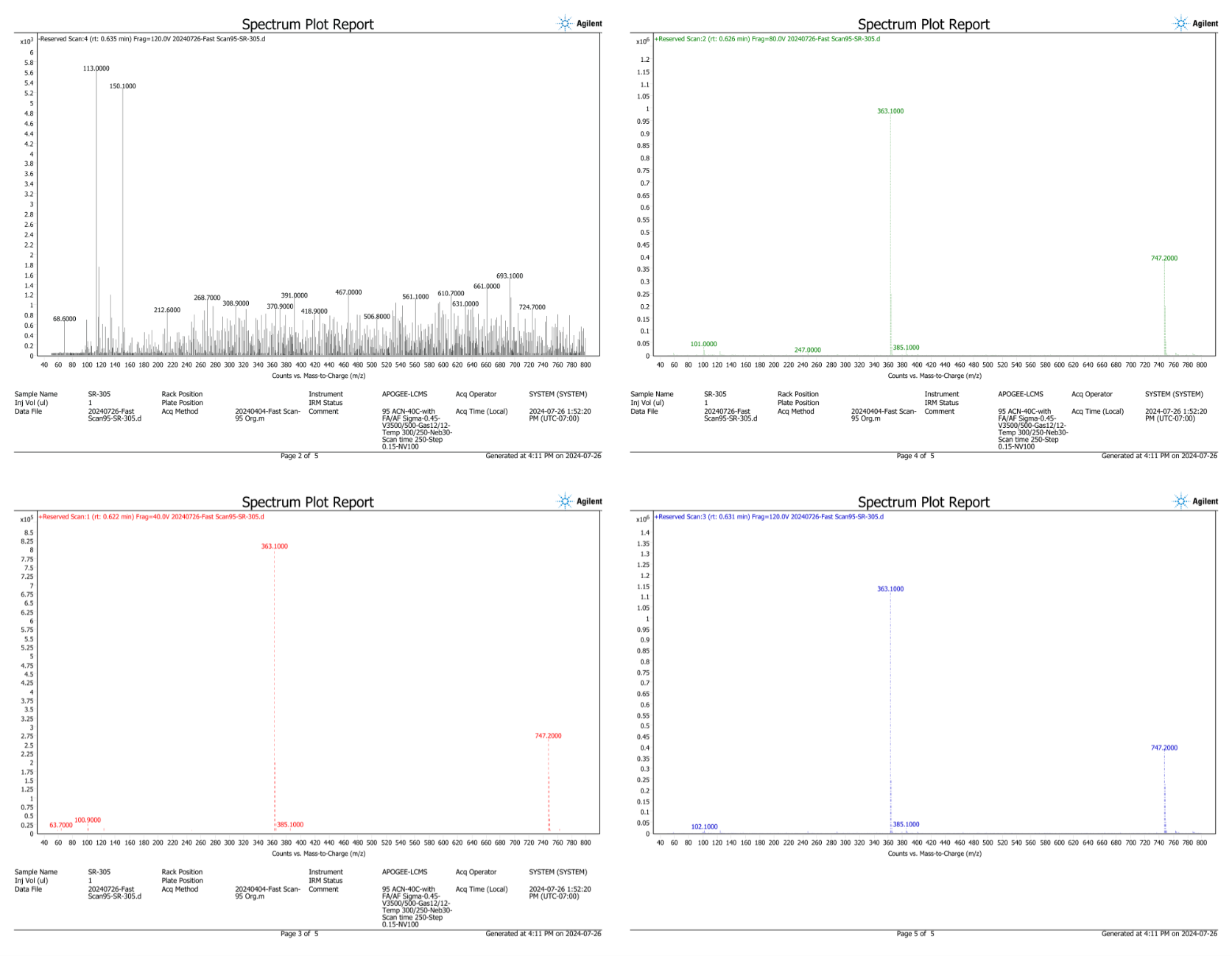
**

**Figure S11**: ^1^H NMR spectrum (400 MHz, DMSO-*d*_6_) of compound **9d**

**
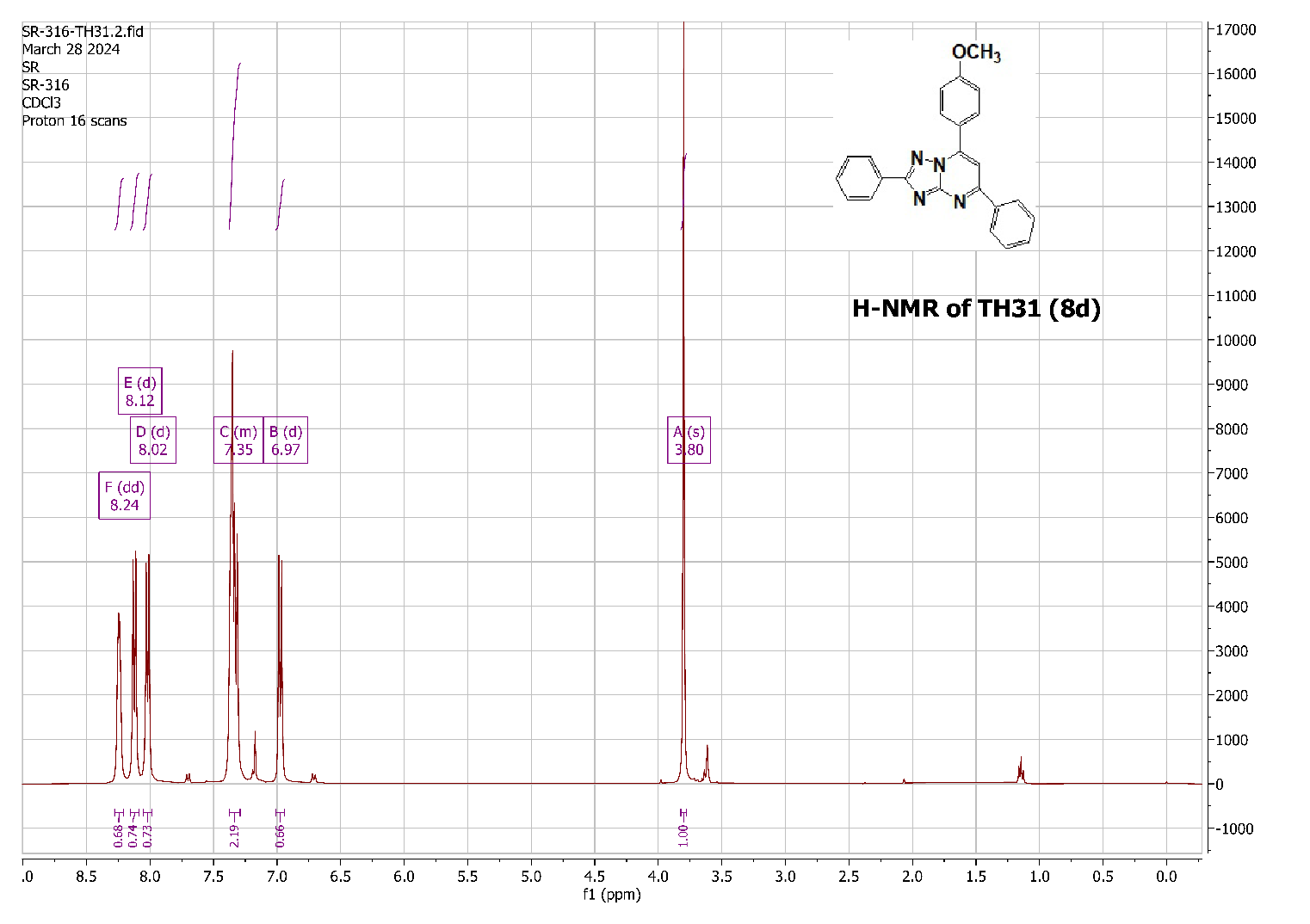
**

**Figure S12**: Expanded ^1^H NMR spectrum (400 MHz, DMSO-*d*_6_) of compound **9d**

**
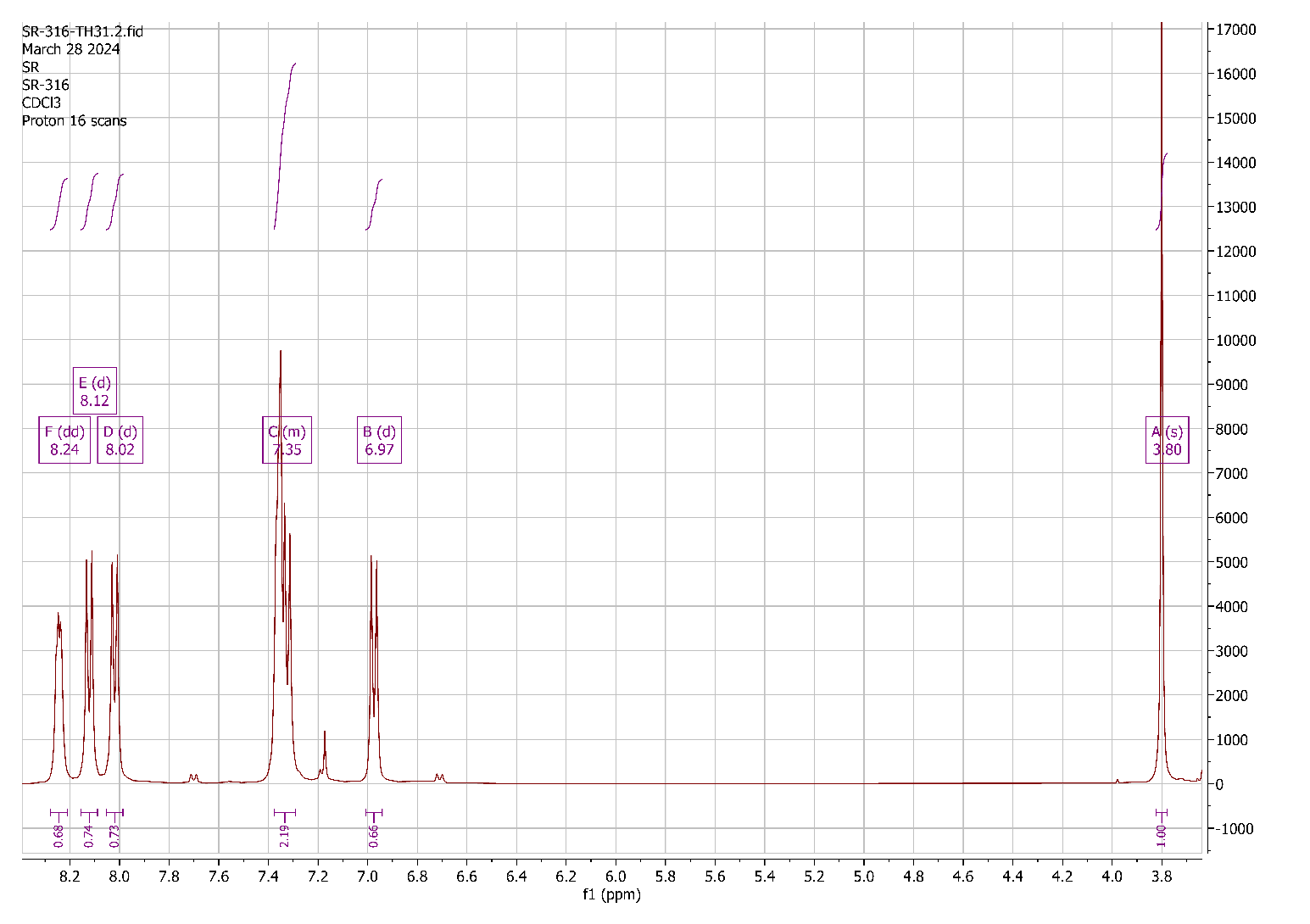
**

**
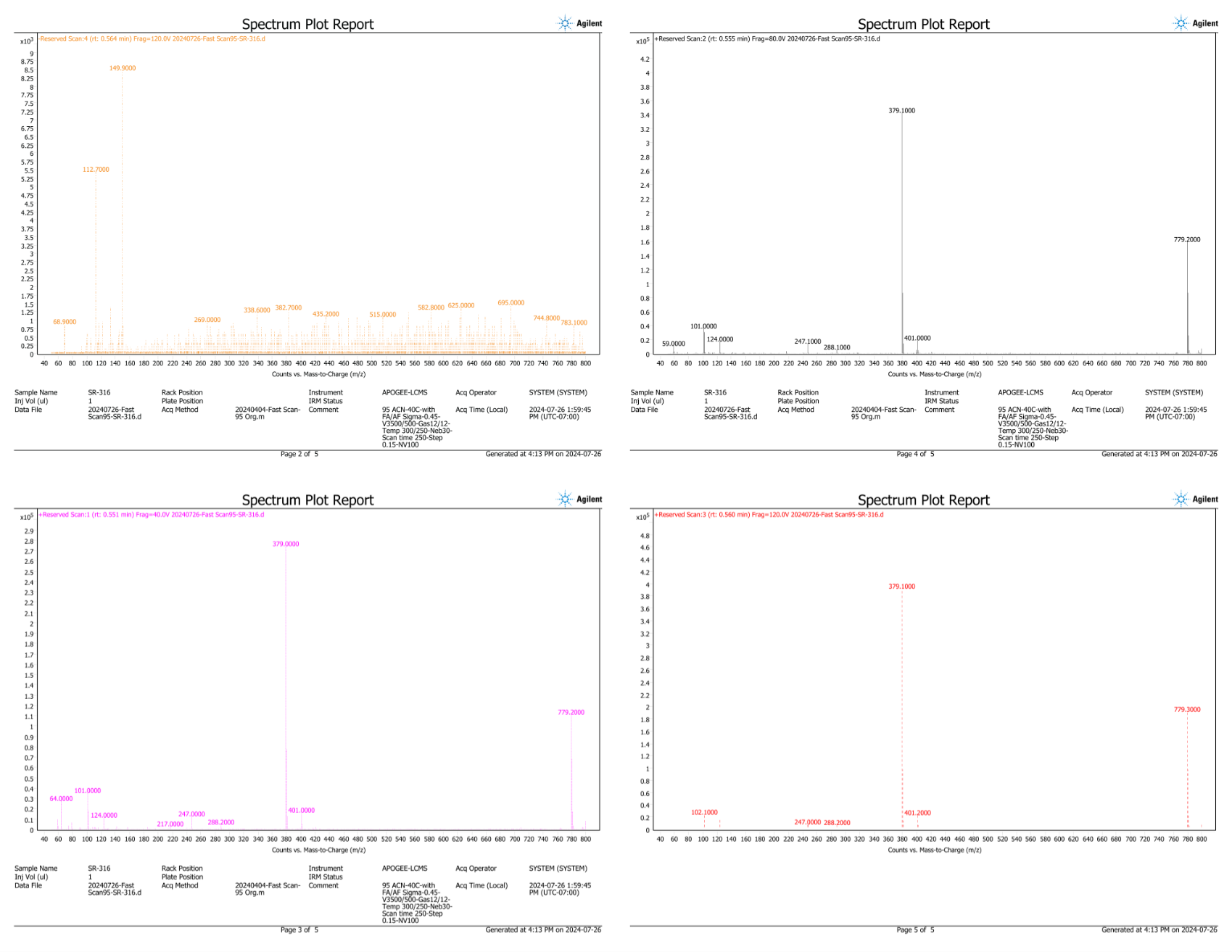
Figure S13**: LC-MS collated spectrum of compound **9d**

**Figure S14**: ^1^H NMR spectrum (400 MHz, DMSO-*d*_6_) of compound **9e**

**
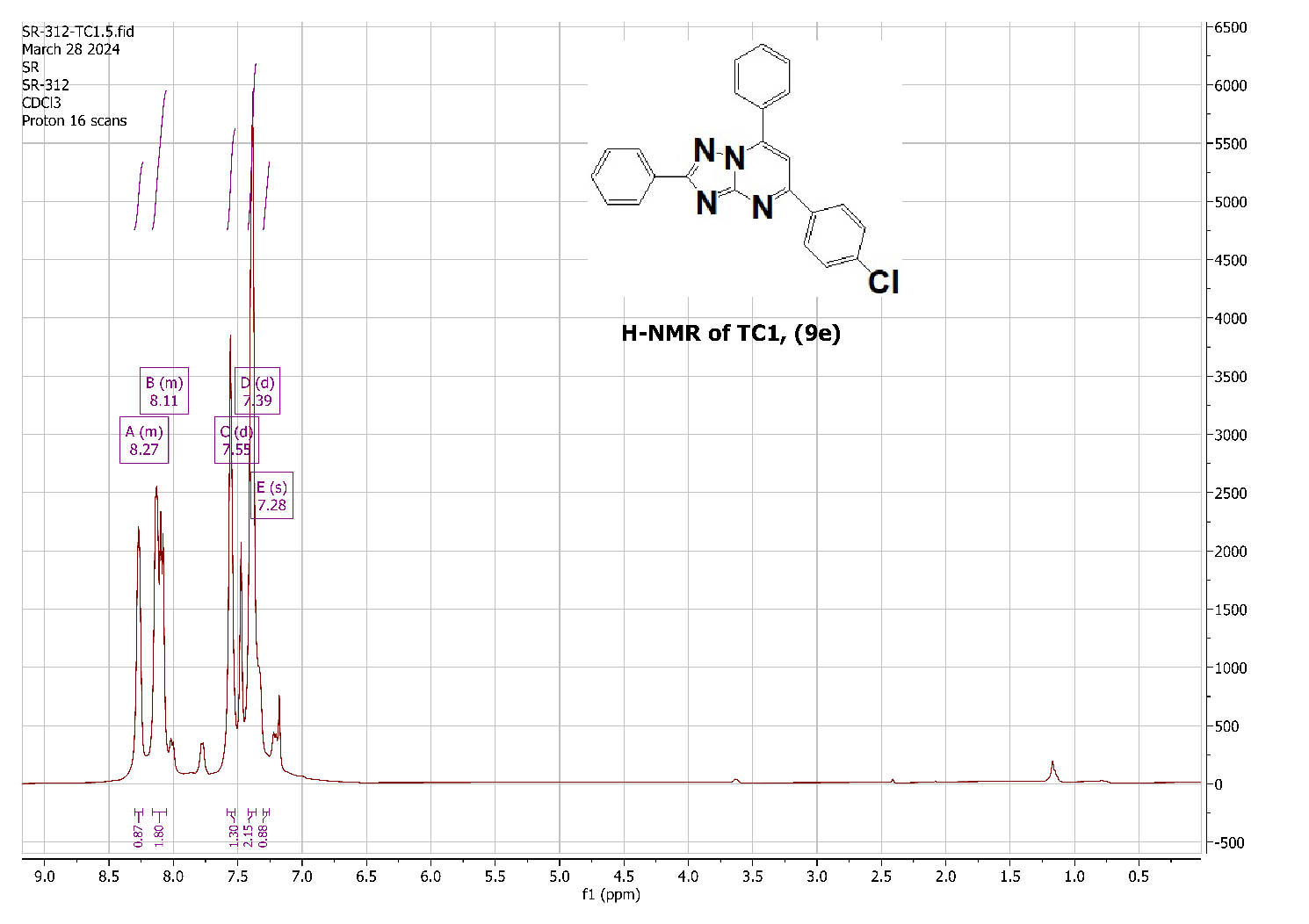
**

**Figure S15**:Expanded ^1^H NMR spectrum (400 MHz, DMSO-*d*_6_) of compound **9e**

**
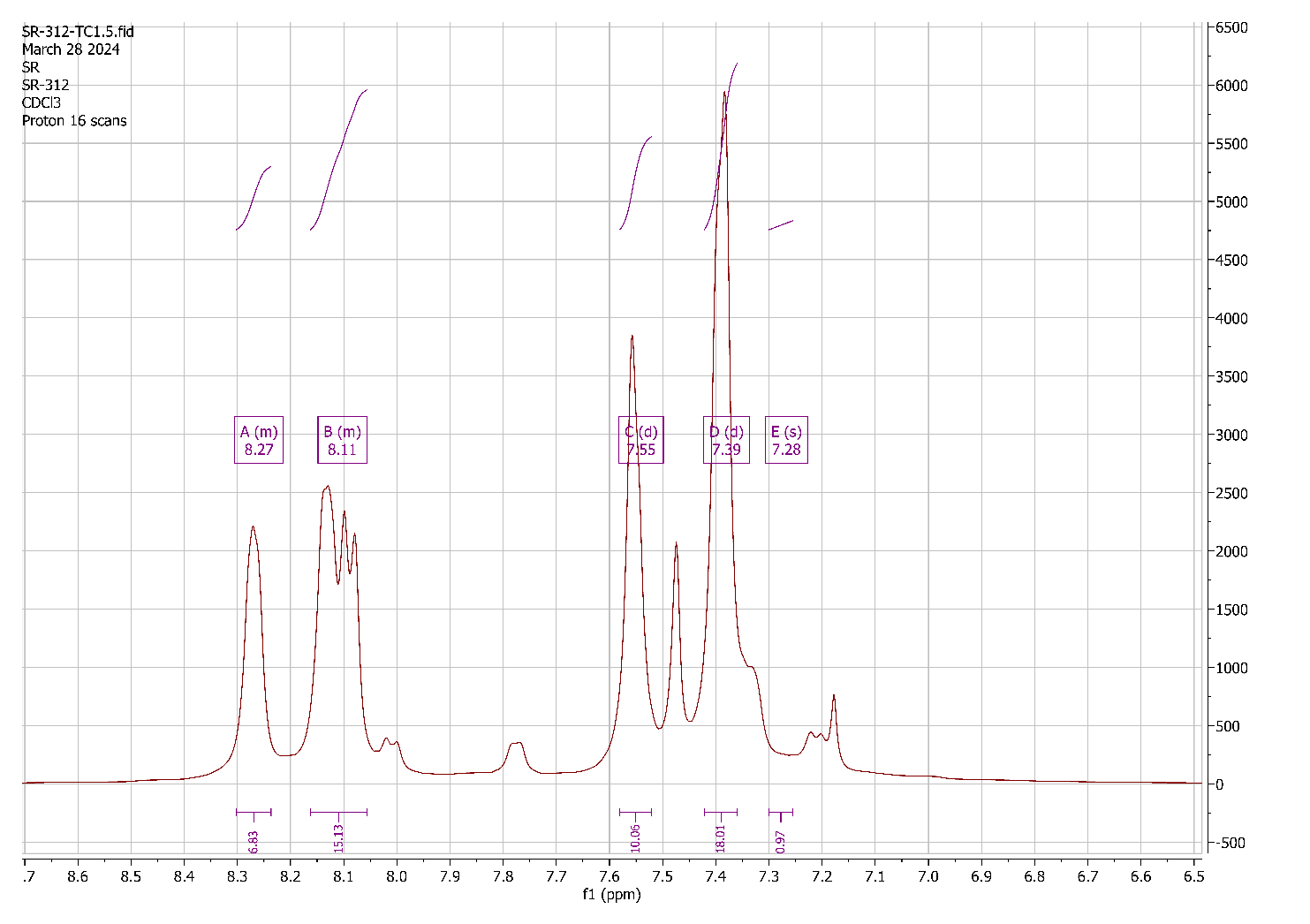
**

**
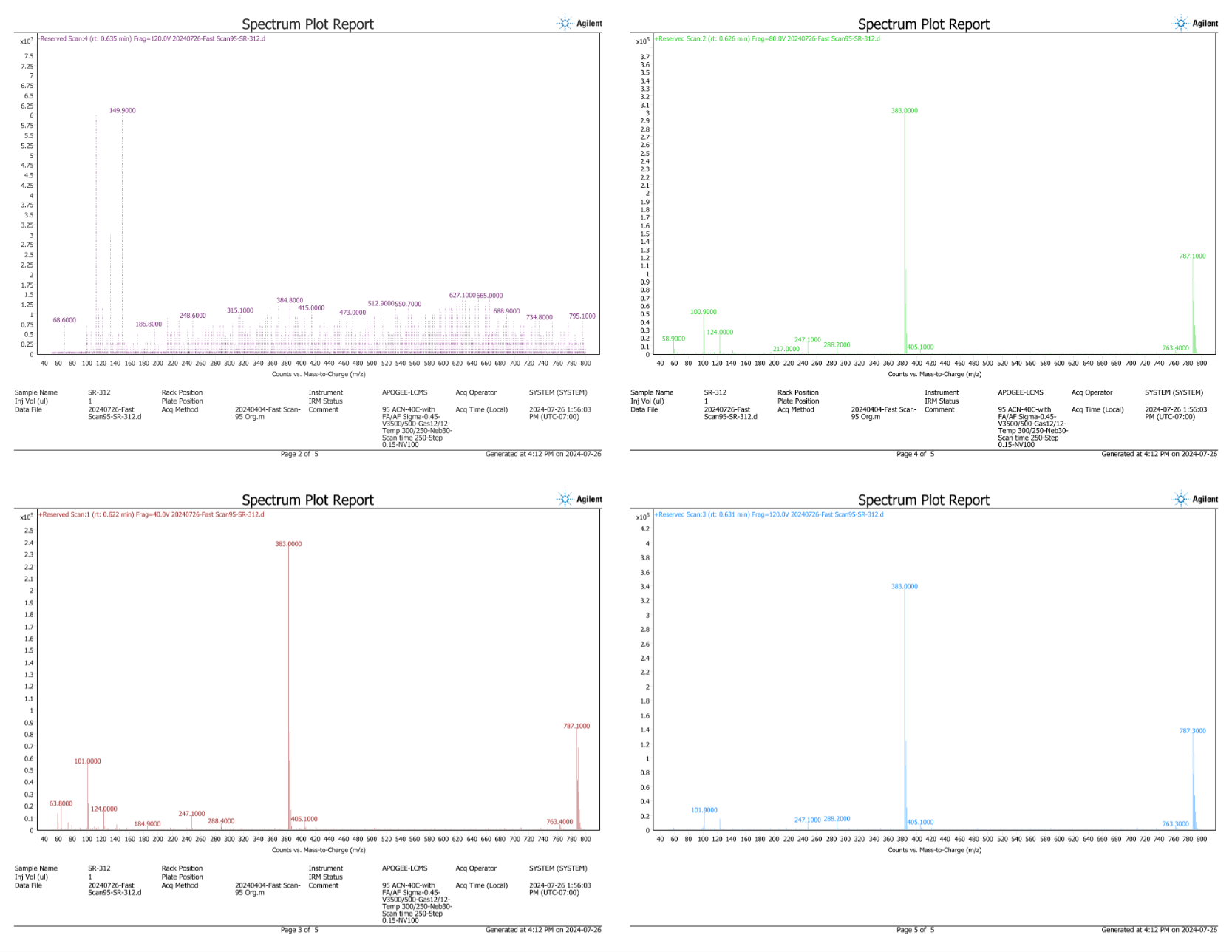
Figure S16**: LC-MS collated spectrum of compound **9e**

**Figure S17**: ^1^H NMR spectrum (400 MHz, DMSO-*d*_6_) of compound **9f**

**
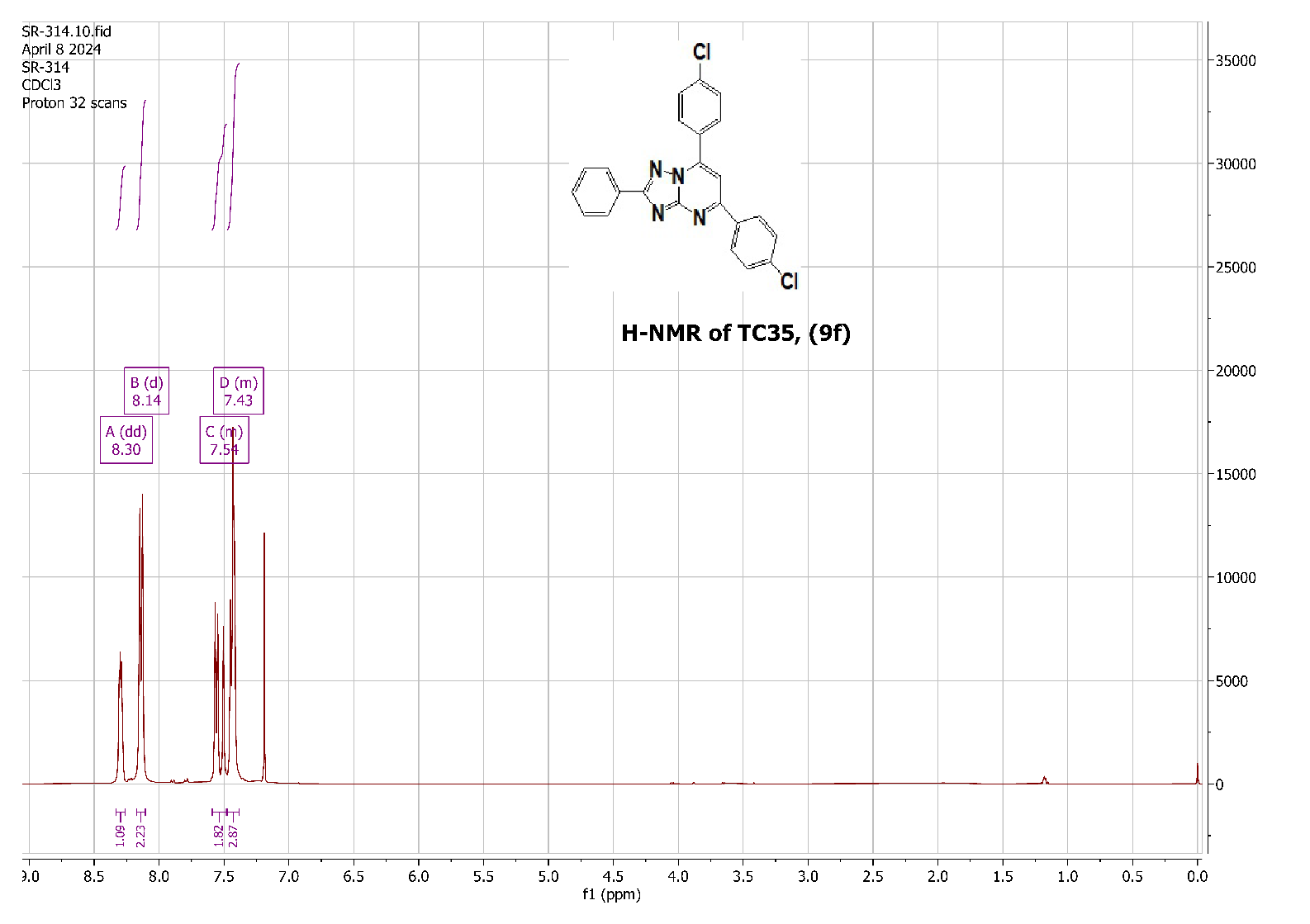
**

**Figure S18**: Expanded ^1^H NMR spectrum (400 MHz, DMSO-*d*_6_) of compound **9f**

**
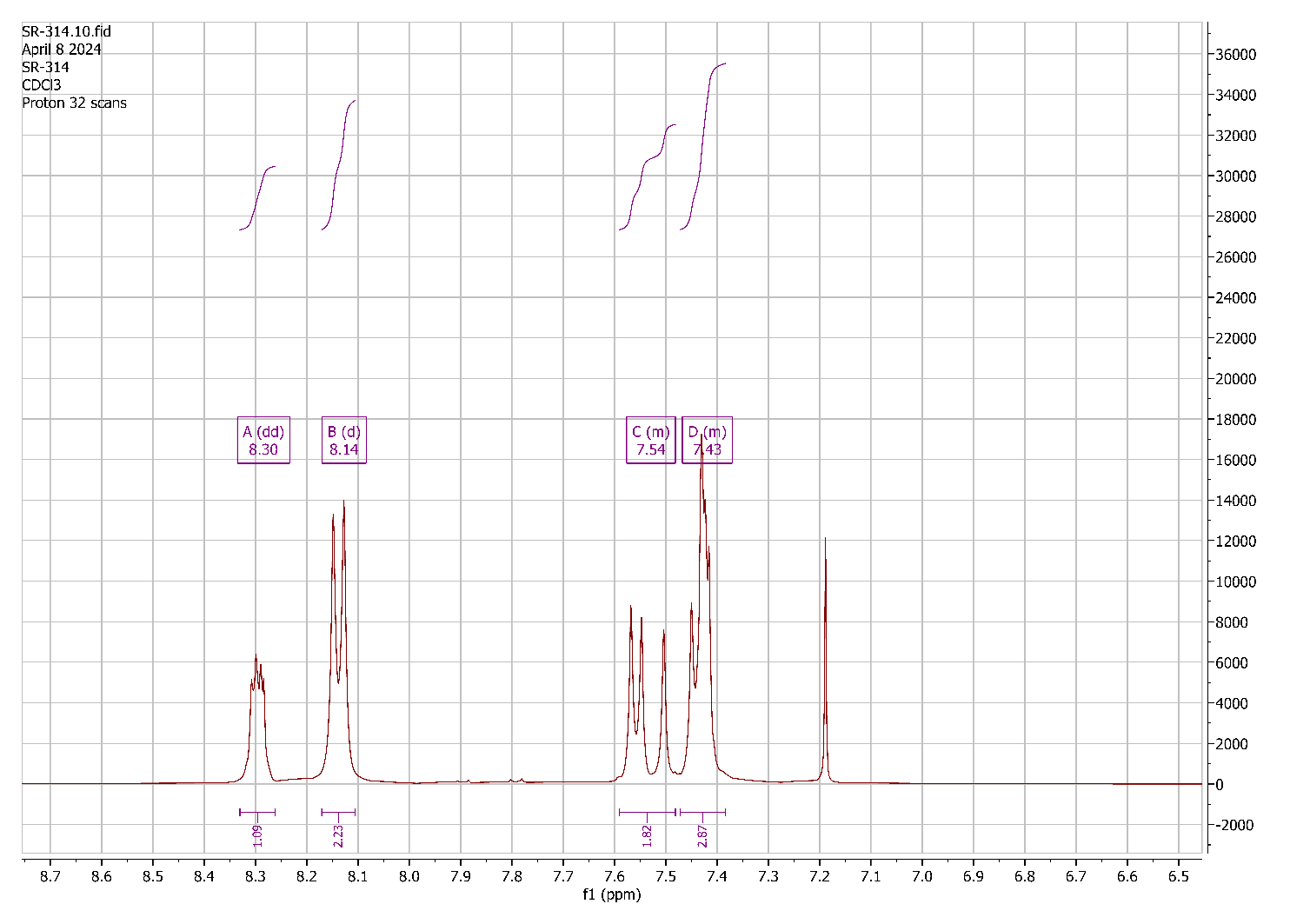
**

**Figure S19**: LC-MS collated spectrum of compound **9f**

**
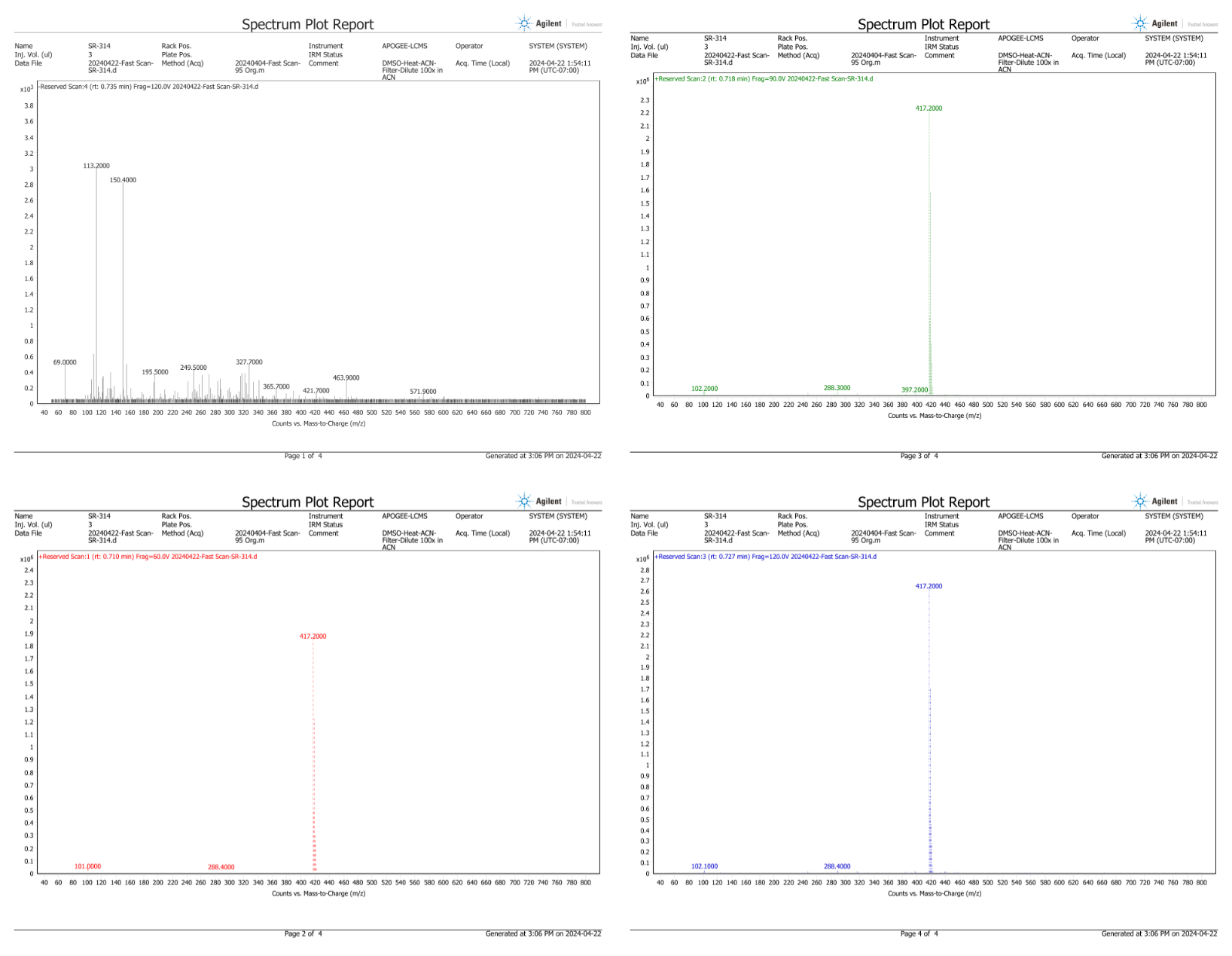
**

**
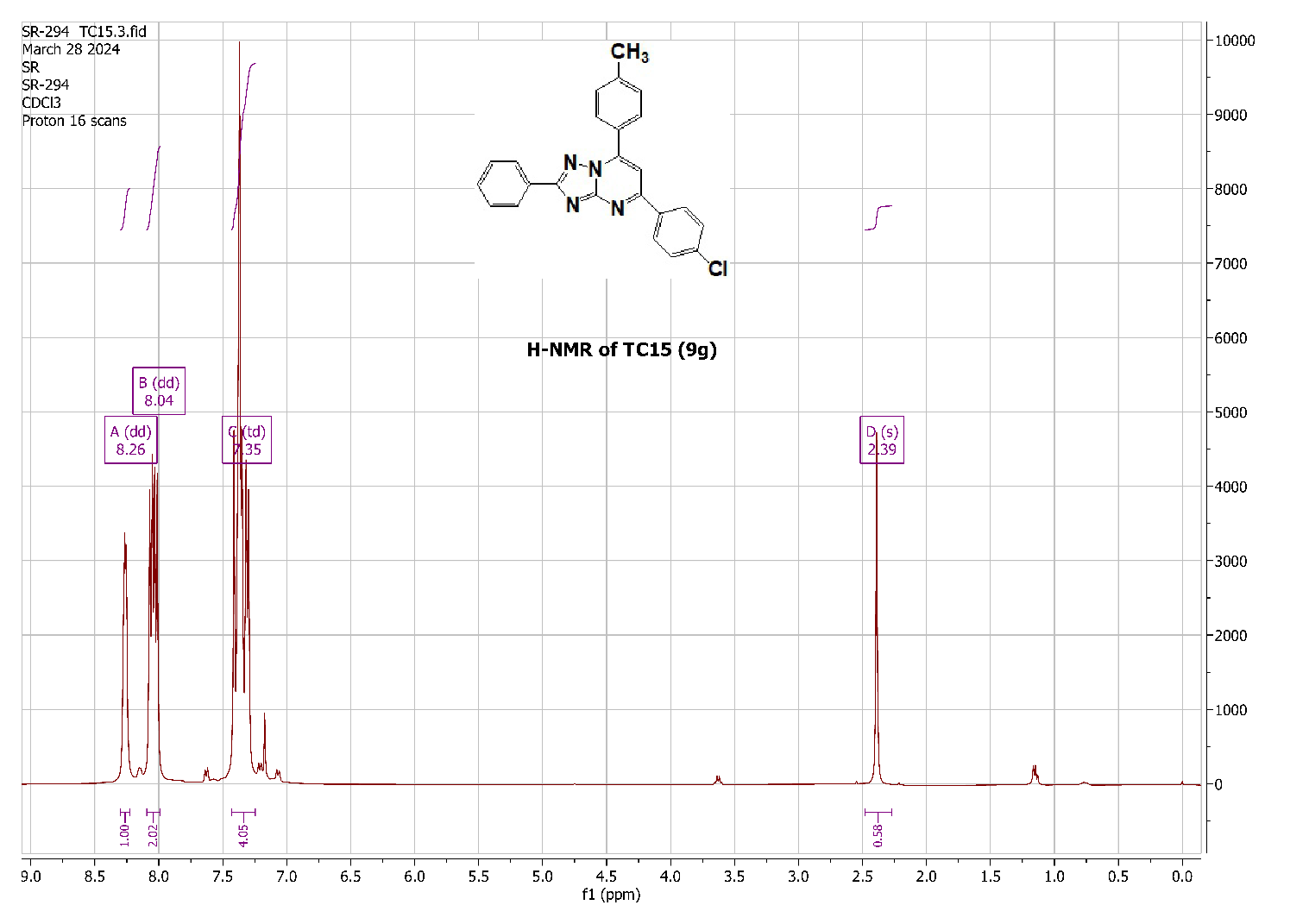
Figure S20**: ^1^H NMR spectrum (400 MHz, DMSO-*d*_6_) of compound **9g**

**Figure S21**: Expanded ^1^H NMR spectrum (400 MHz, DMSO-*d*_6_) of compound **9g**

**
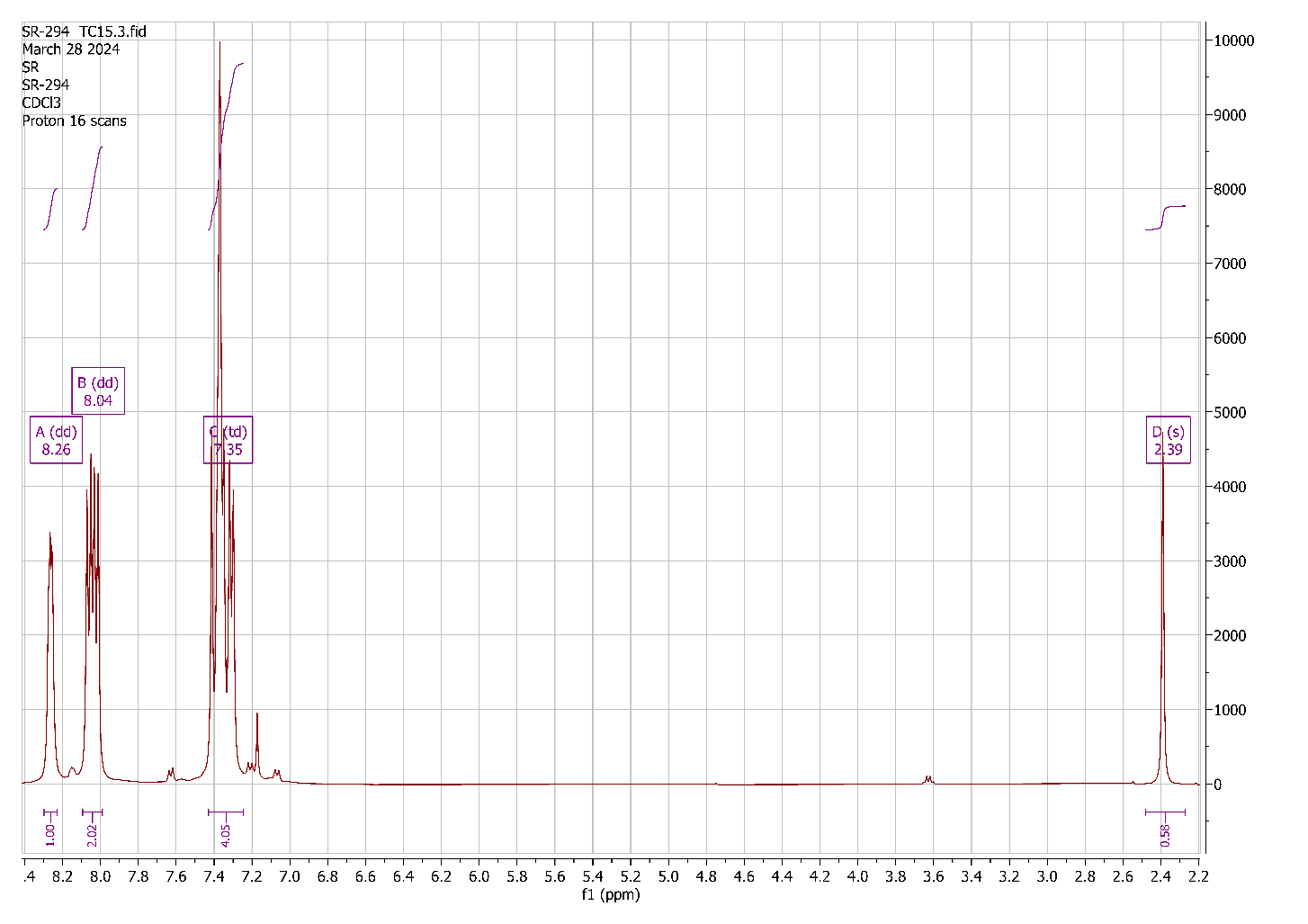
**

**
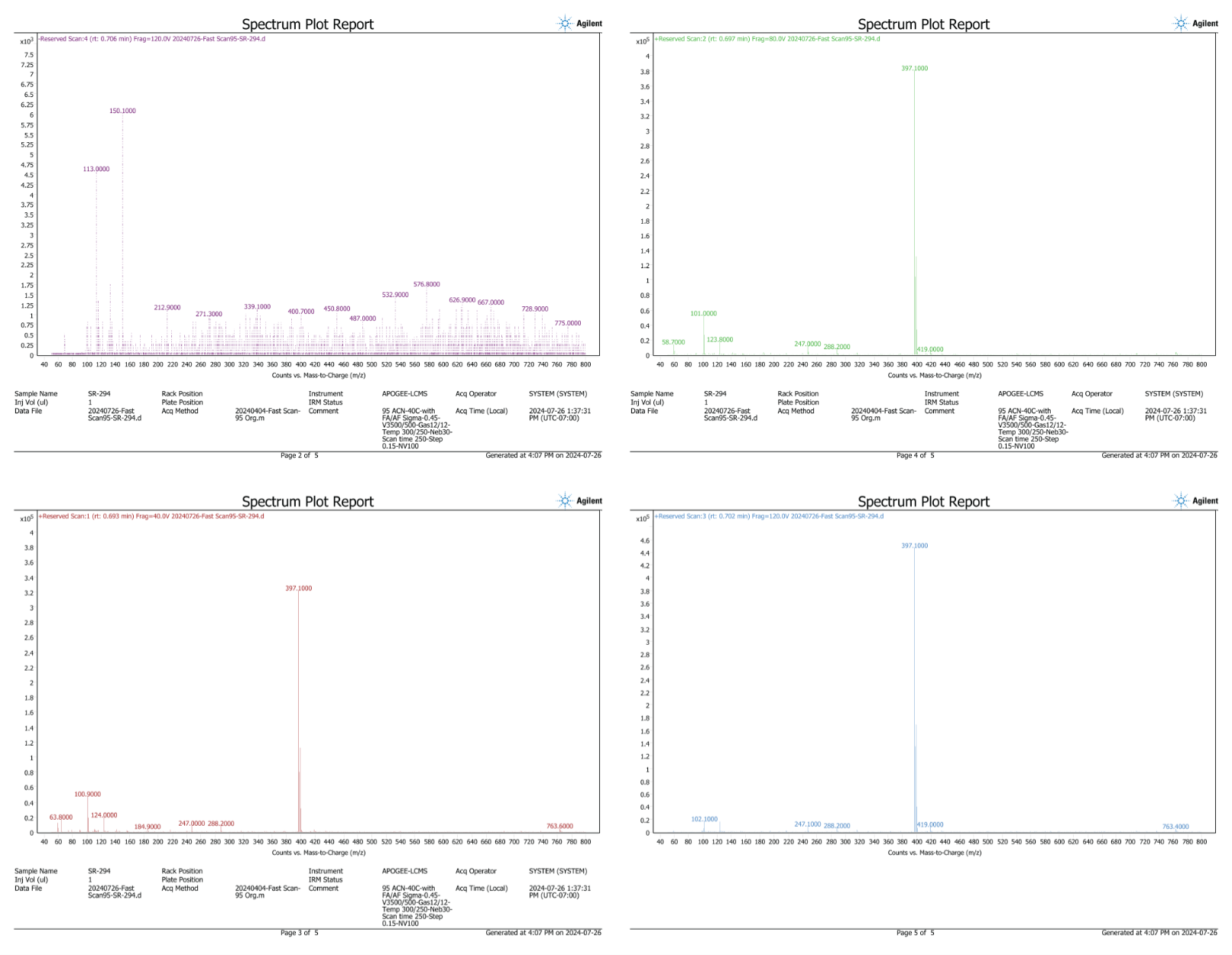
Figure S22**: LC-MS collated spectrum of compound **9g**

**
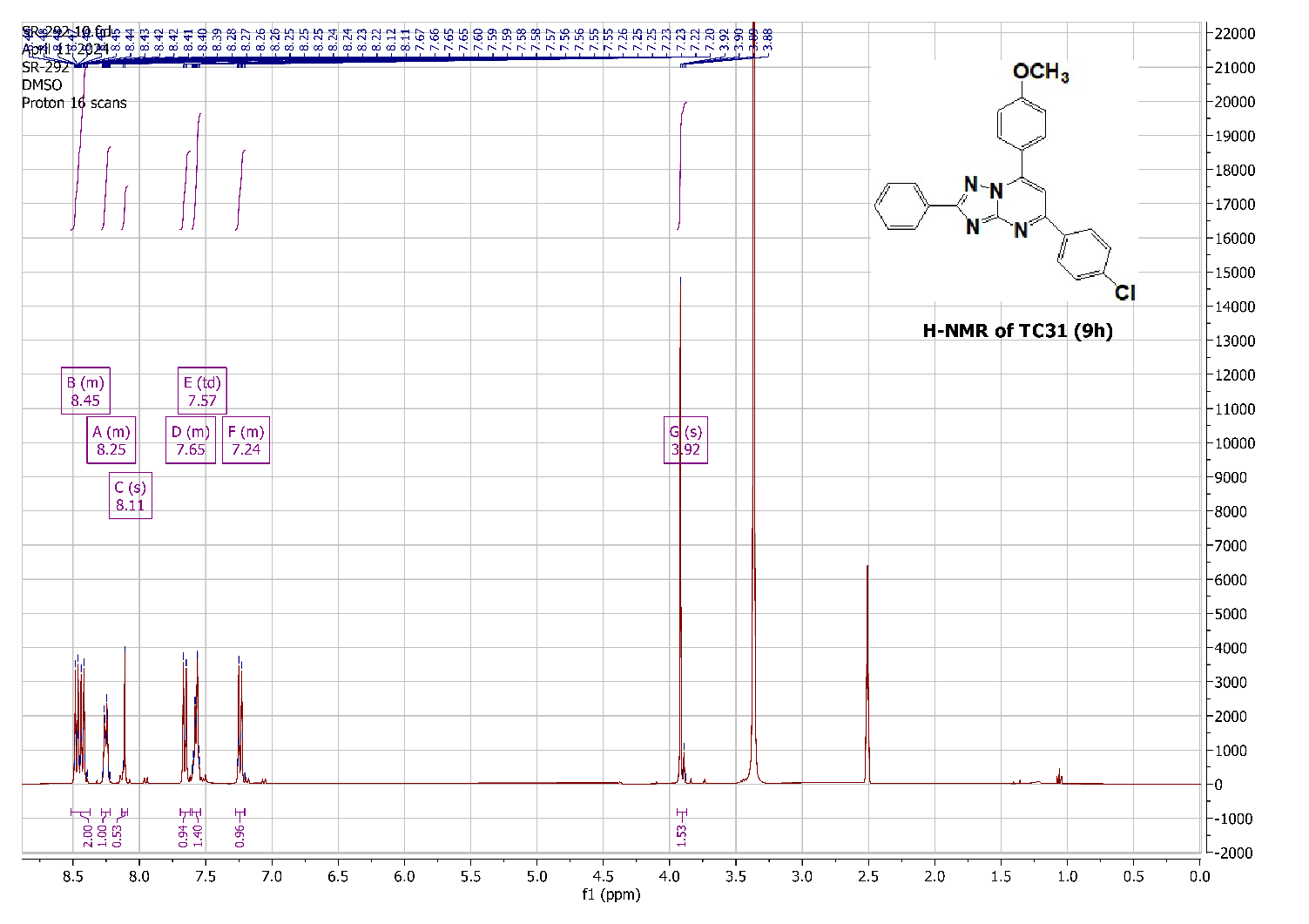
Figure S23**: ^1^H NMR spectrum (400 MHz, DMSO-*d*_6_) of compound **9h**

**Figure S24**: Expanded ^1^H NMR spectrum (400 MHz, DMSO-*d*_6_) of compound **9h**

**
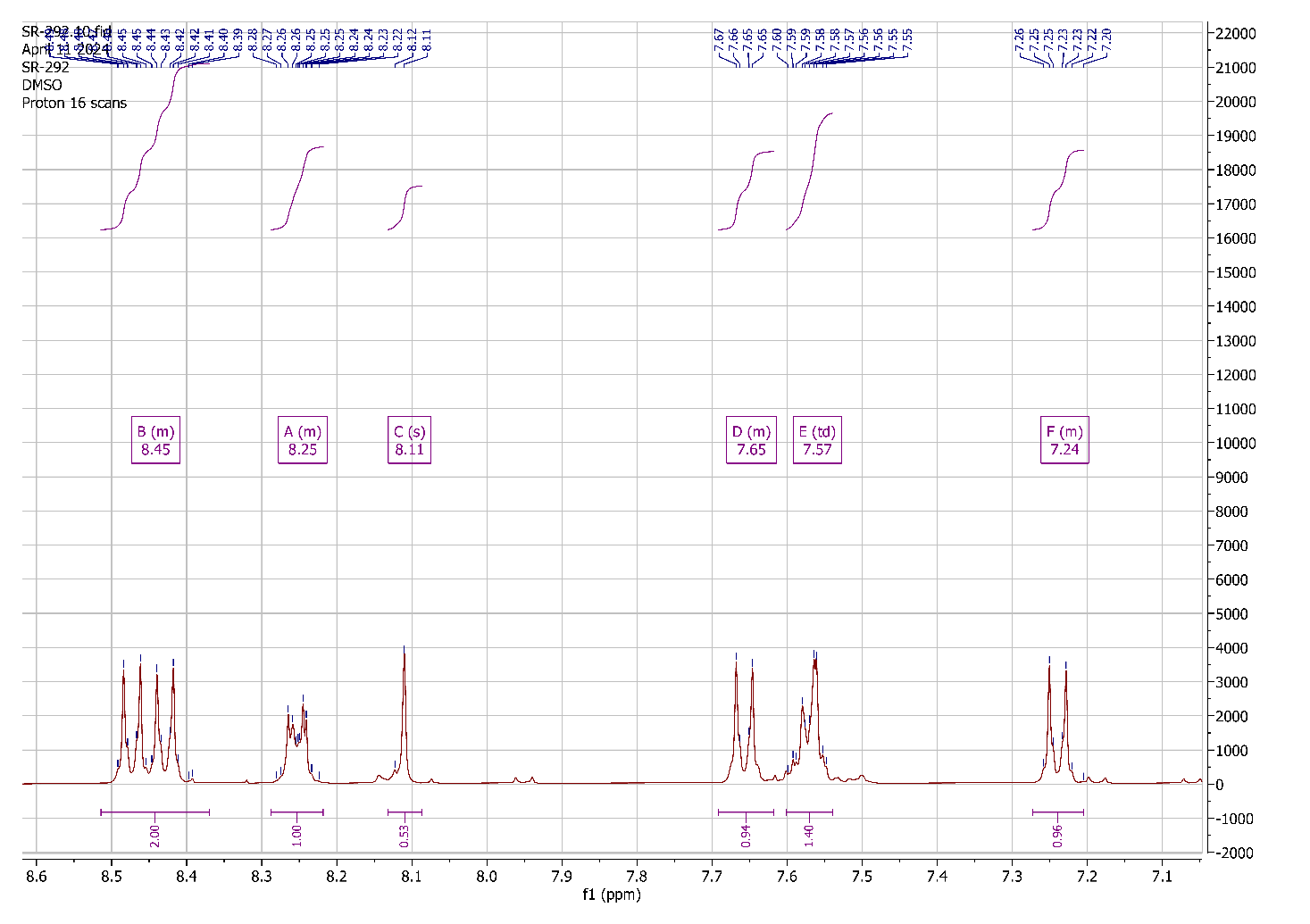
**

**Figure S25**: LC-MS collated spectrum of compound **9h**

**
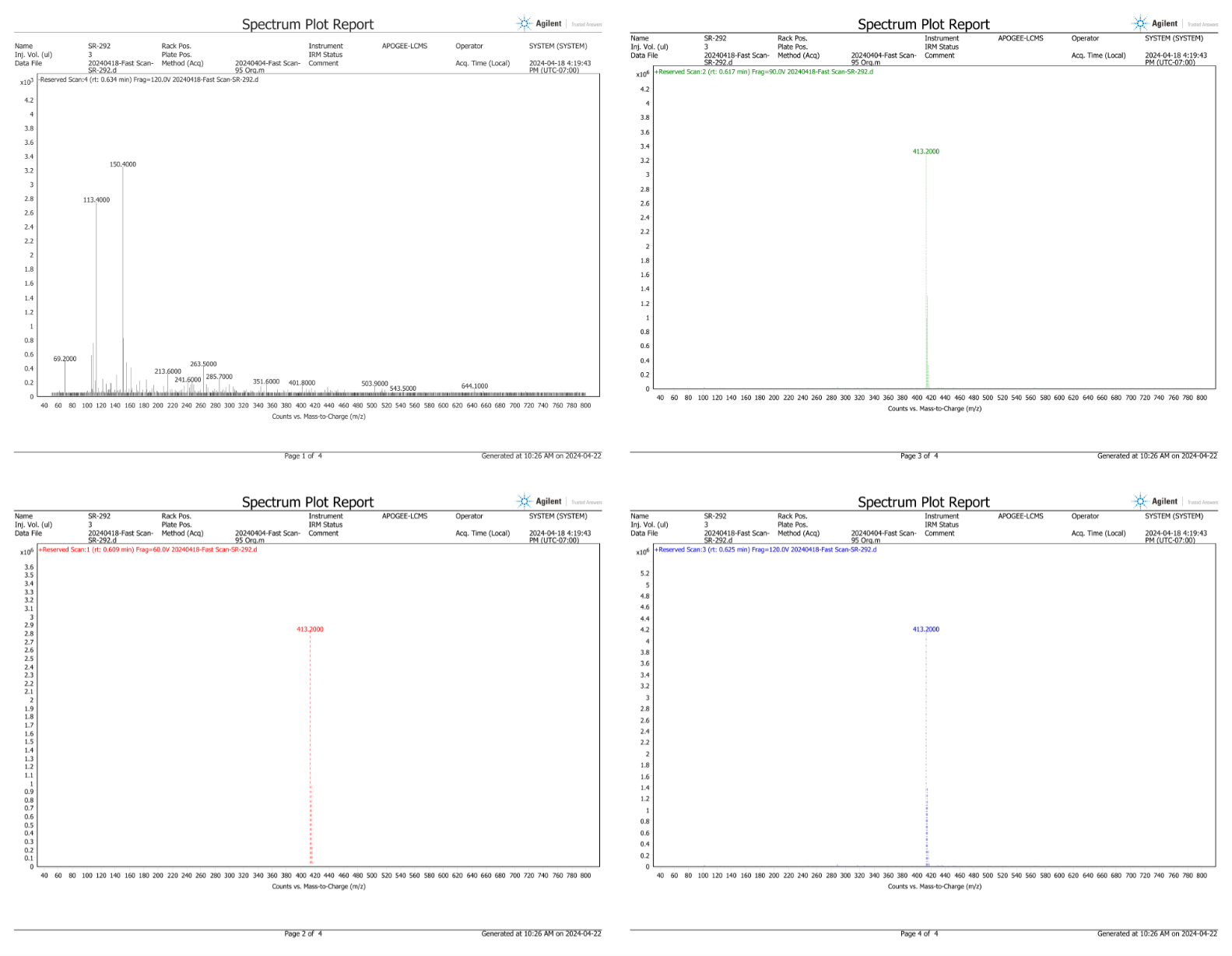
**

**
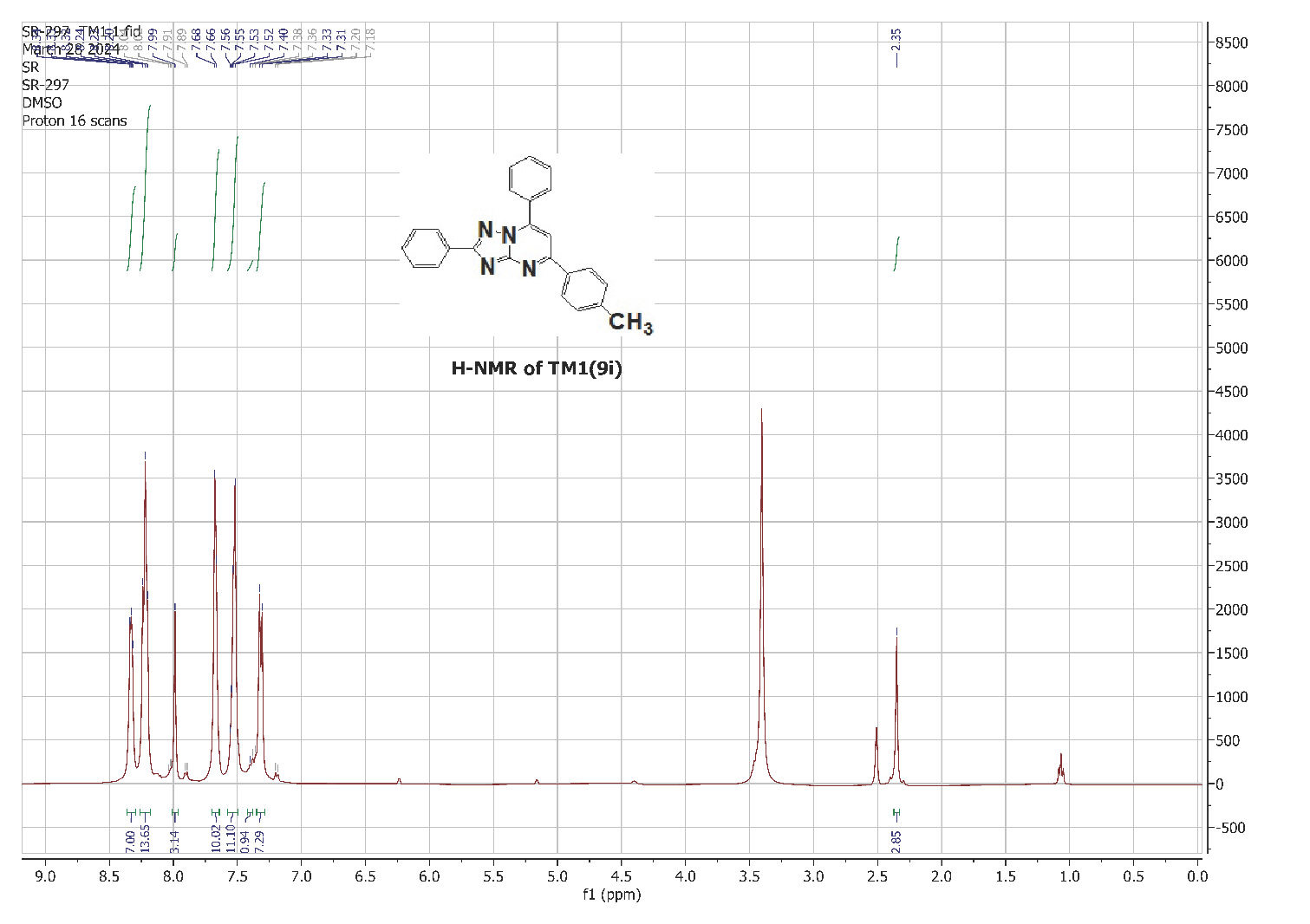
Figure S26**: ^1^H NMR spectrum (400 MHz, DMSO-*d*_6_) of compound **9i**

**Figure S27**: Expanded ^1^H NMR spectrum (400 MHz, DMSO-*d*_6_) of compound **9i**

**
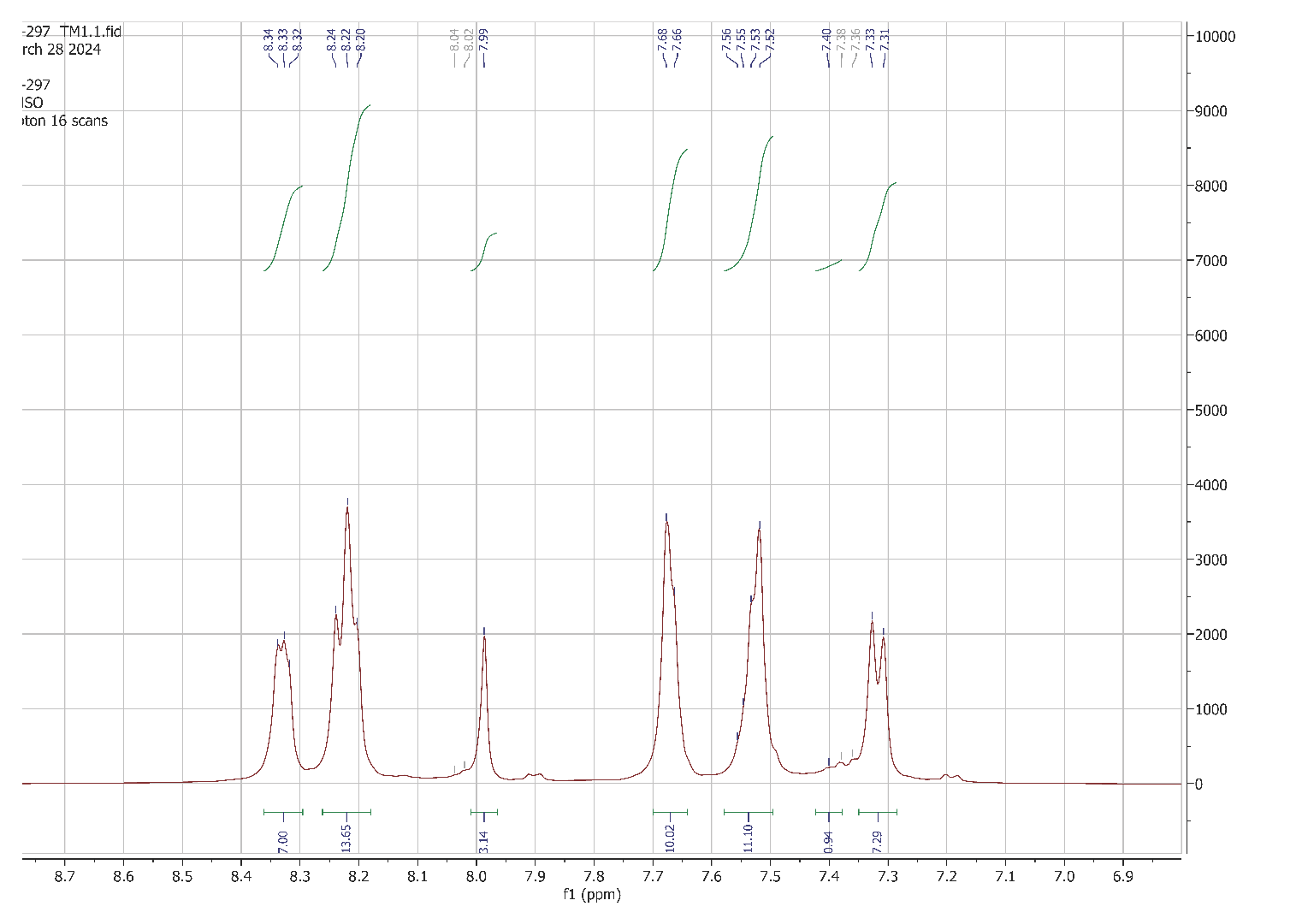
**

**
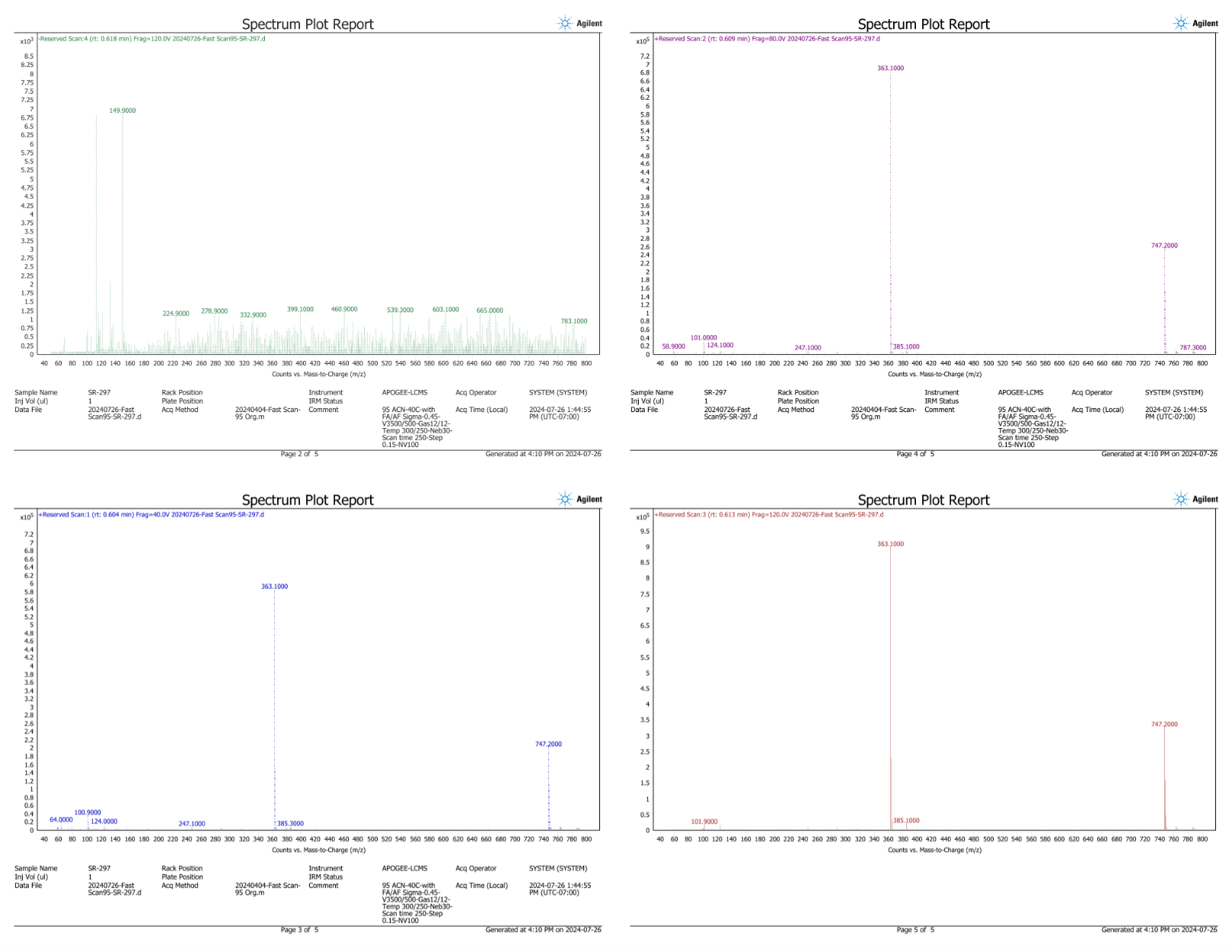
Figure S28**: LC-MS collated spectrum of compound **9i**

**Figure S29**: ^1^H NMR spectrum (400 MHz, DMSO-*d*_6_) of compound **9j**

**
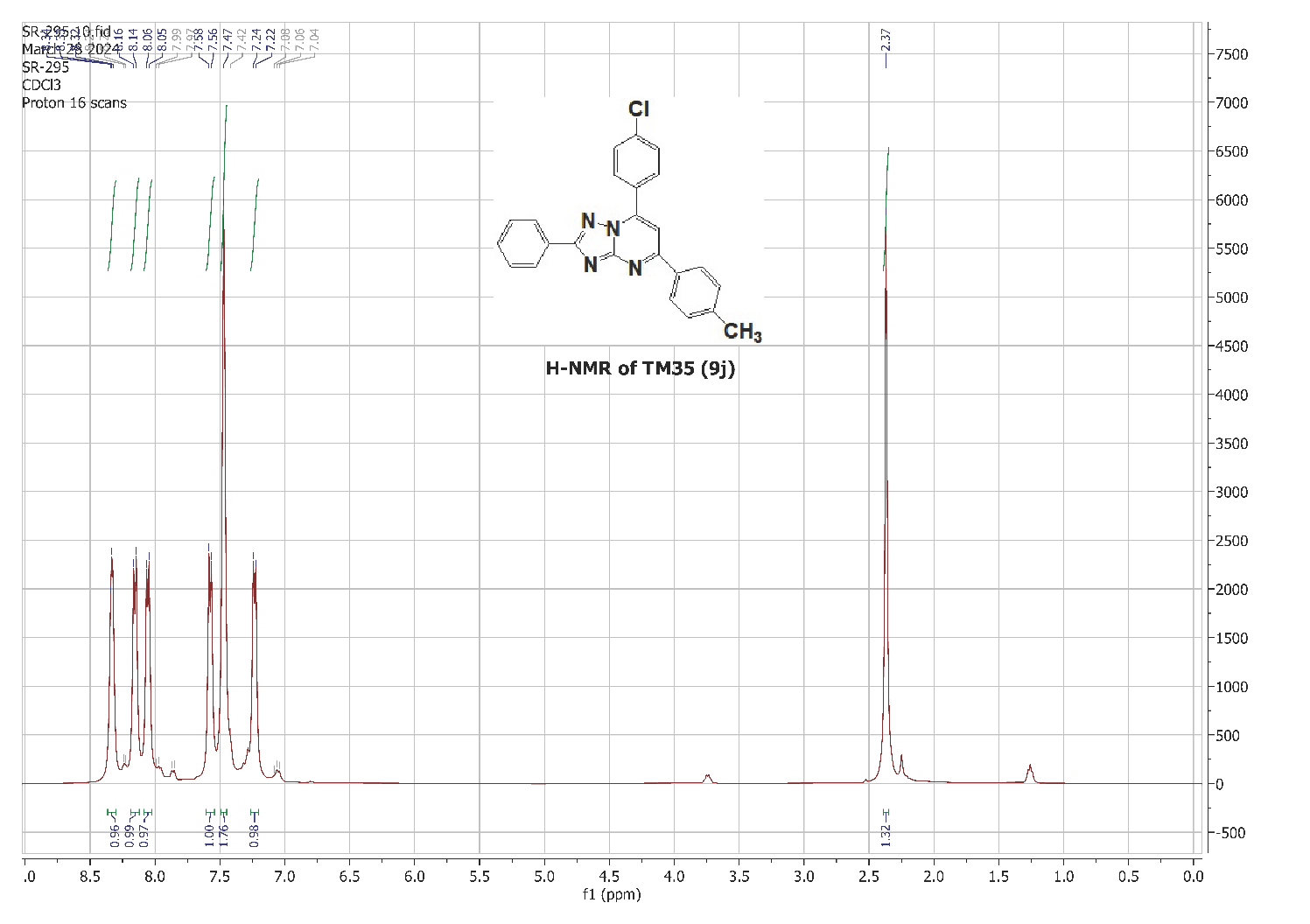
**

**
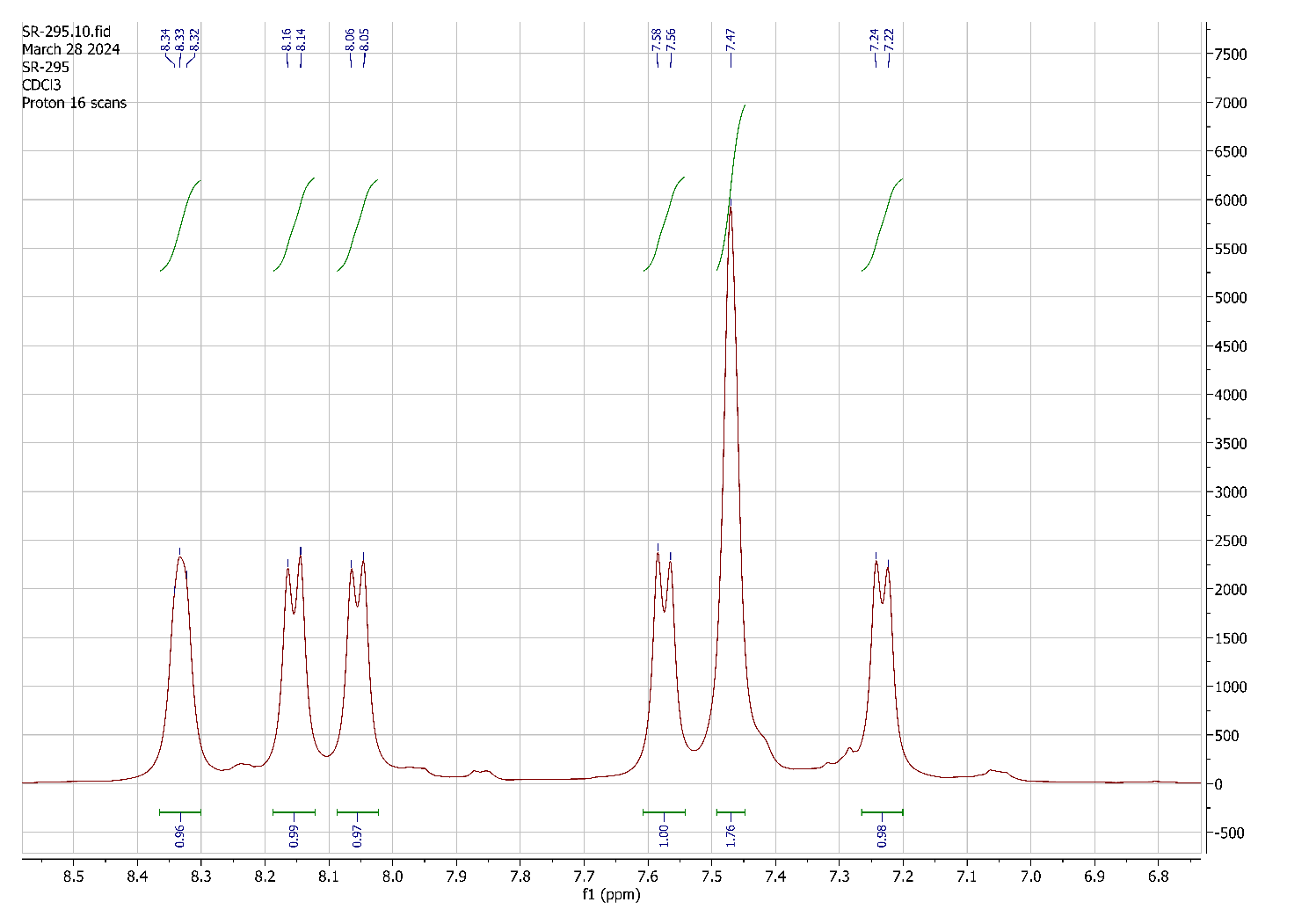
Figure S30**: Expanded ^1^H NMR spectrum (400 MHz, DMSO-*d*_6_) of compound **9j**

**
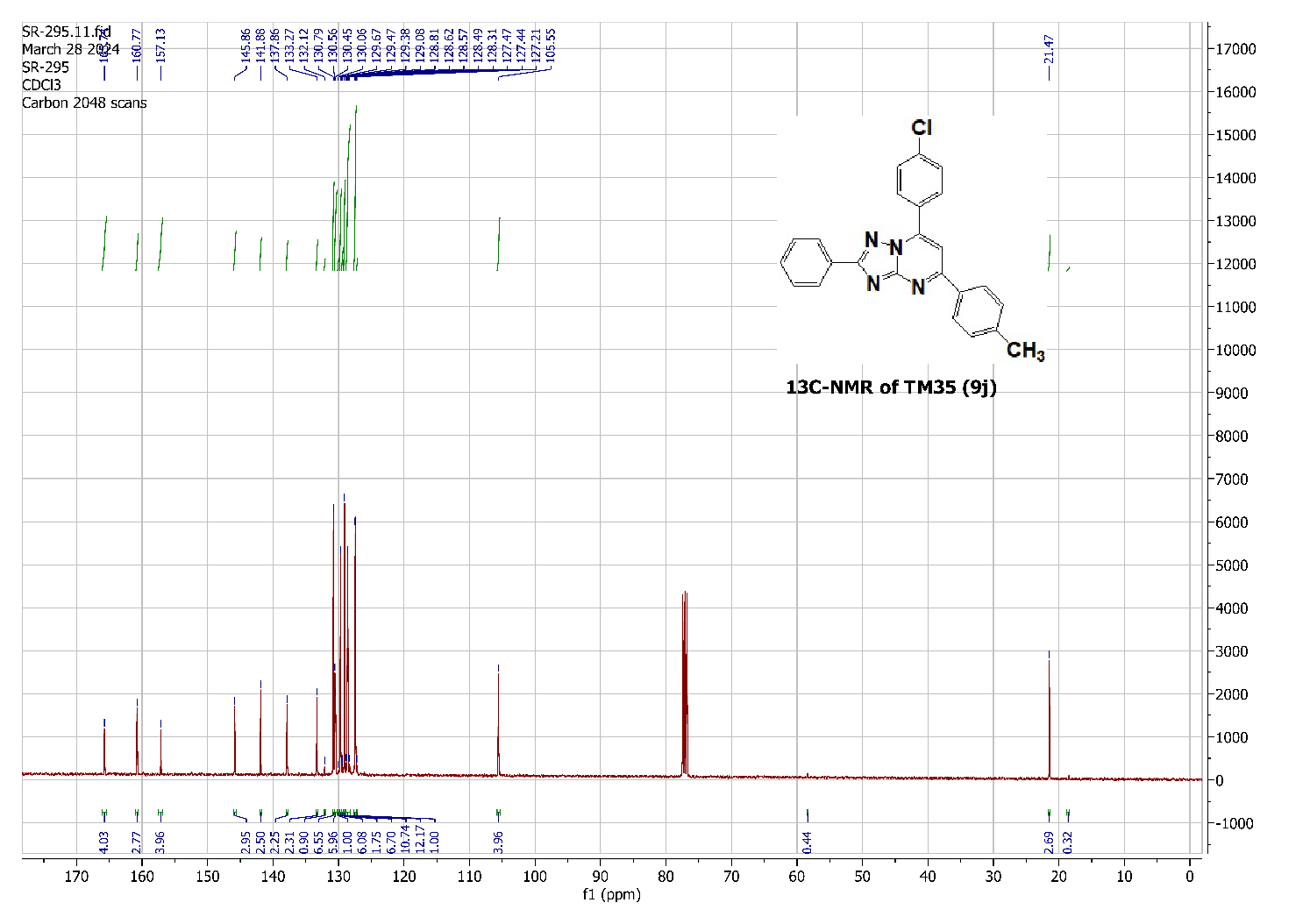
Figure S31**: ^13^C NMR spectrum (100 MHz, DMSO-*d*_6_) of compound **9j**

**
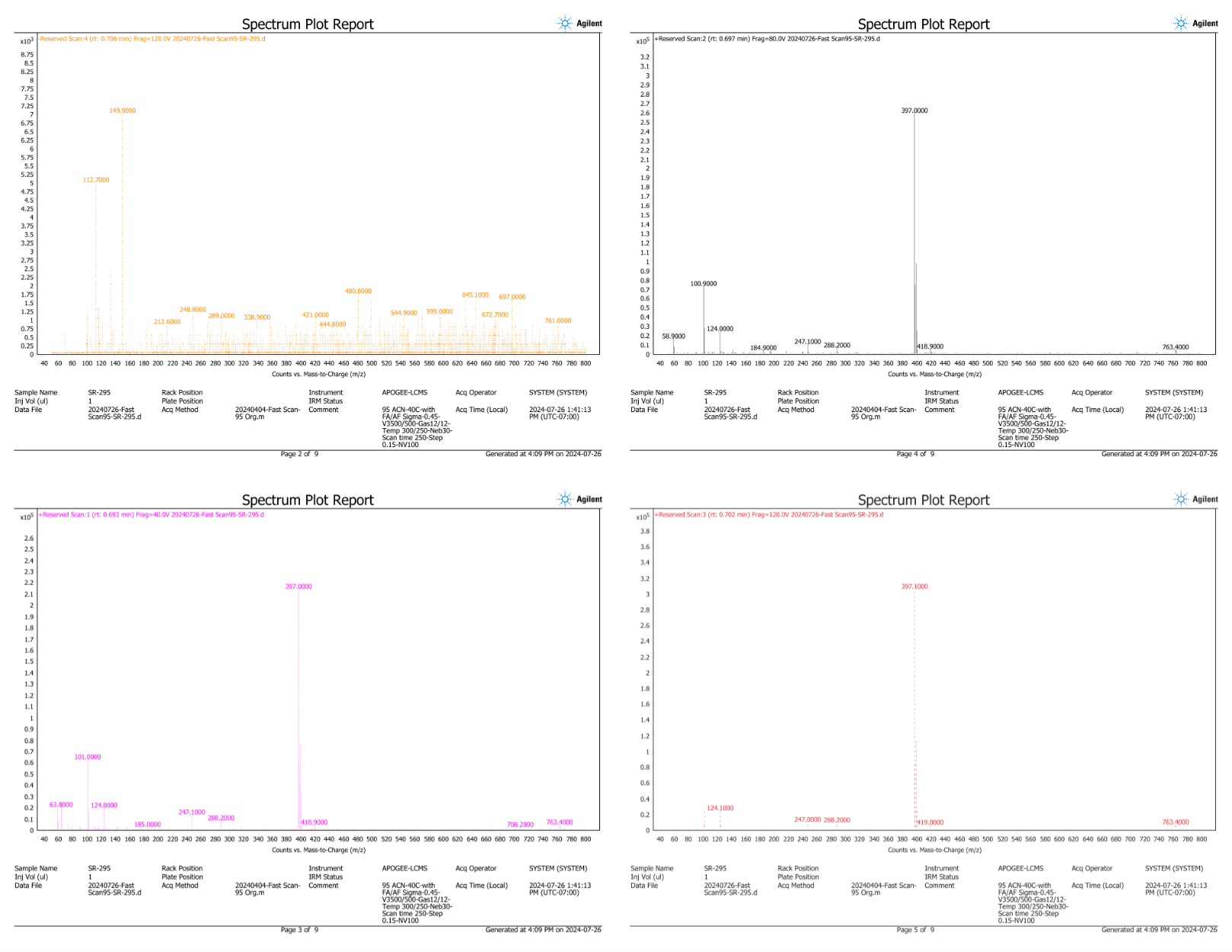
Figure S32**: LC-MS collated spectrum of compound **9j**

**
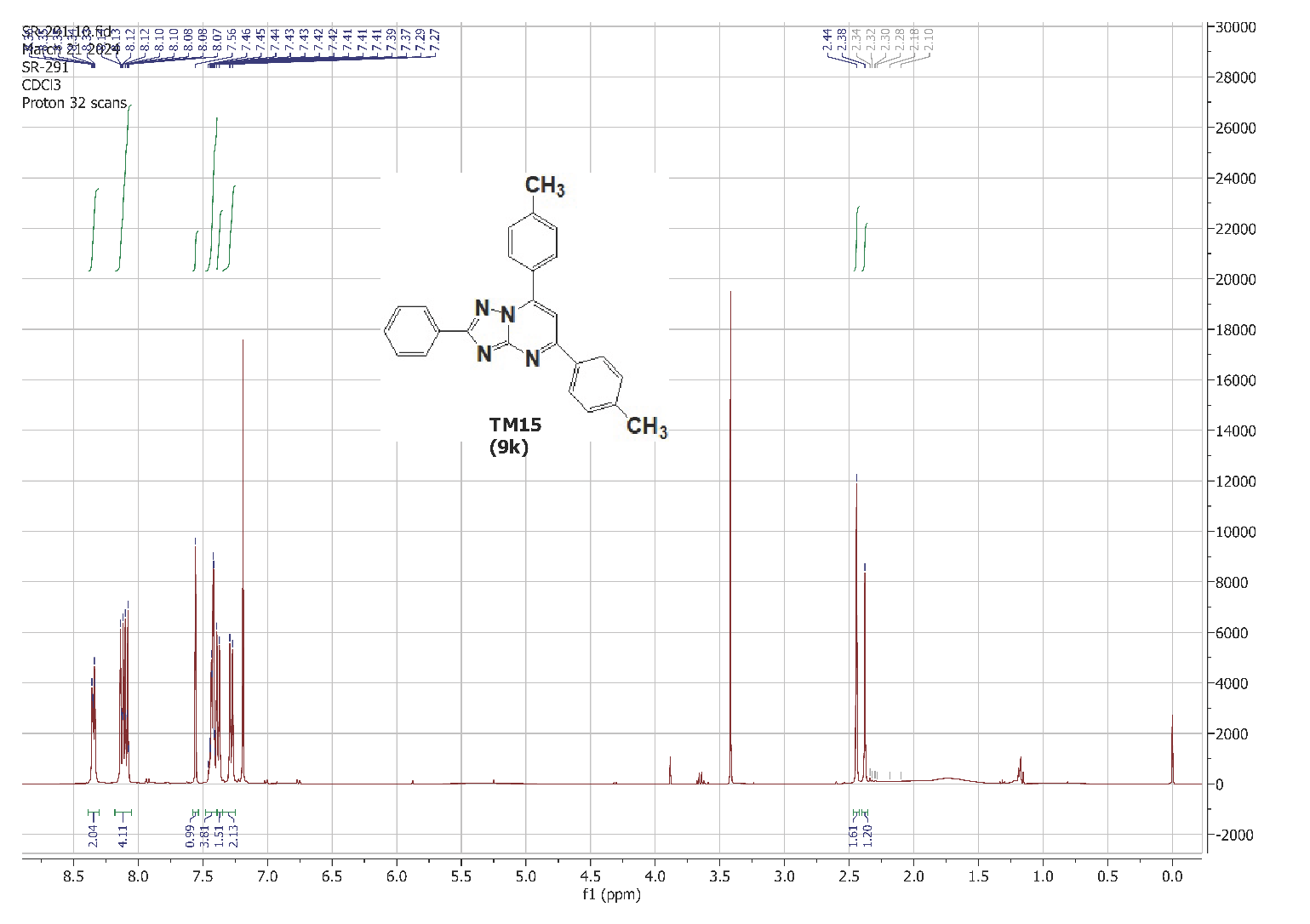
Figure S33**: ^1^H NMR spectrum (400 MHz, DMSO-*d*_6_) of compound **9k**

**Figure S34**: Expanded ^1^H NMR spectrum (400 MHz, DMSO-*d*_6_) of compound **9k**

**
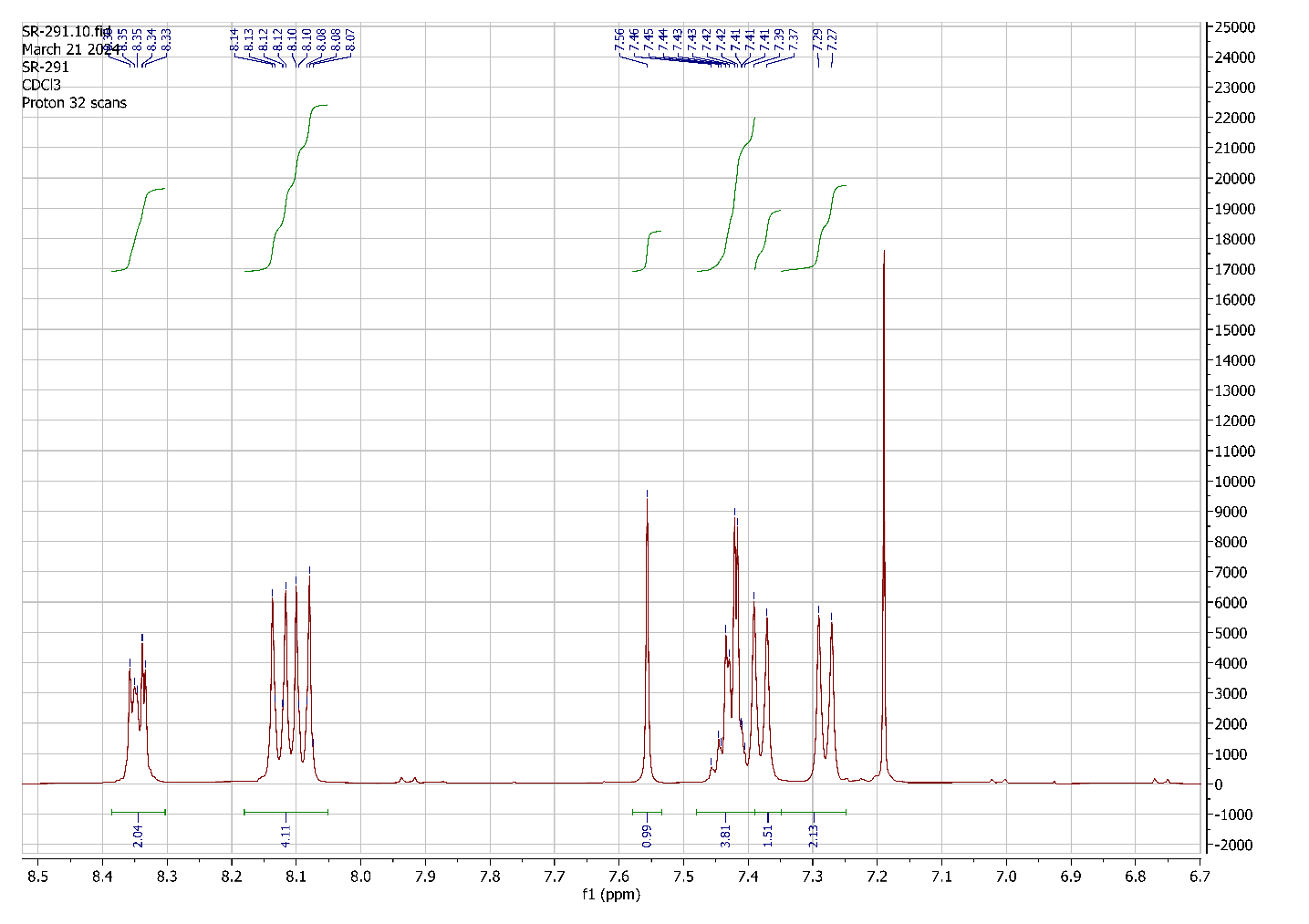
**

**Figure S35**: ^13^C NMR spectrum (100 MHz, DMSO-*d*_6_) of compound **9k**

**
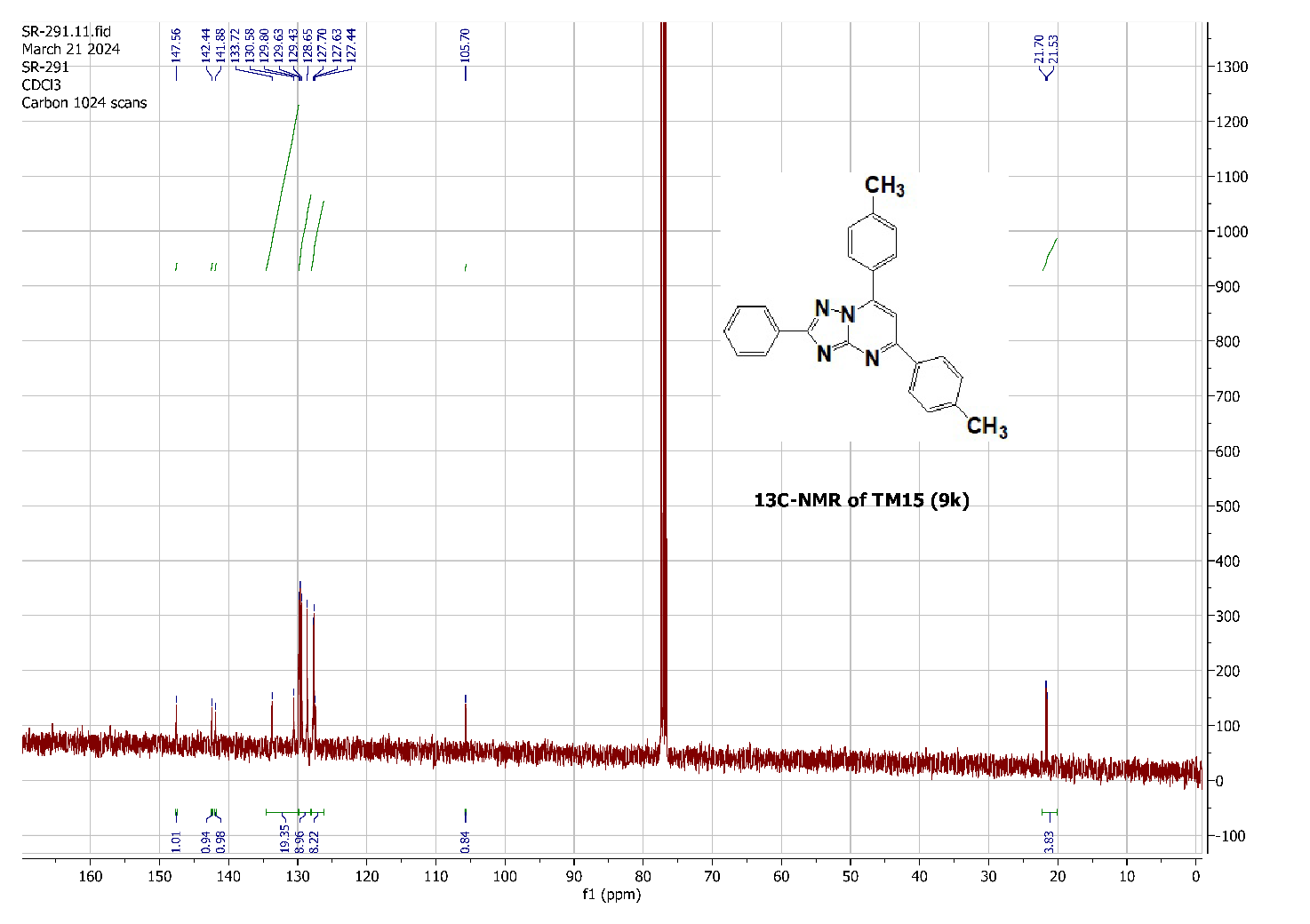
**

**
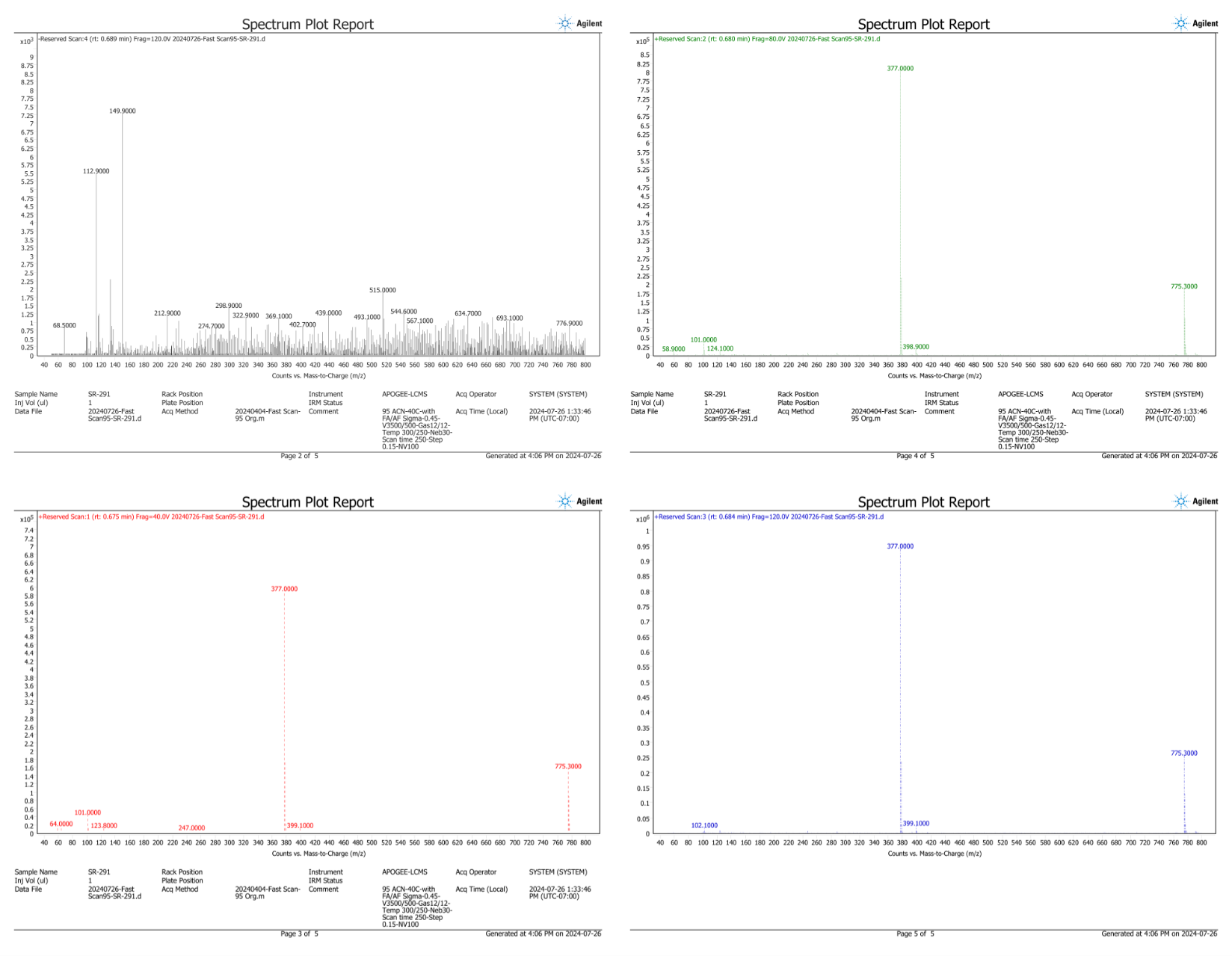
Figure S36**: LC-MS collated spectrum of compound **9k**

**
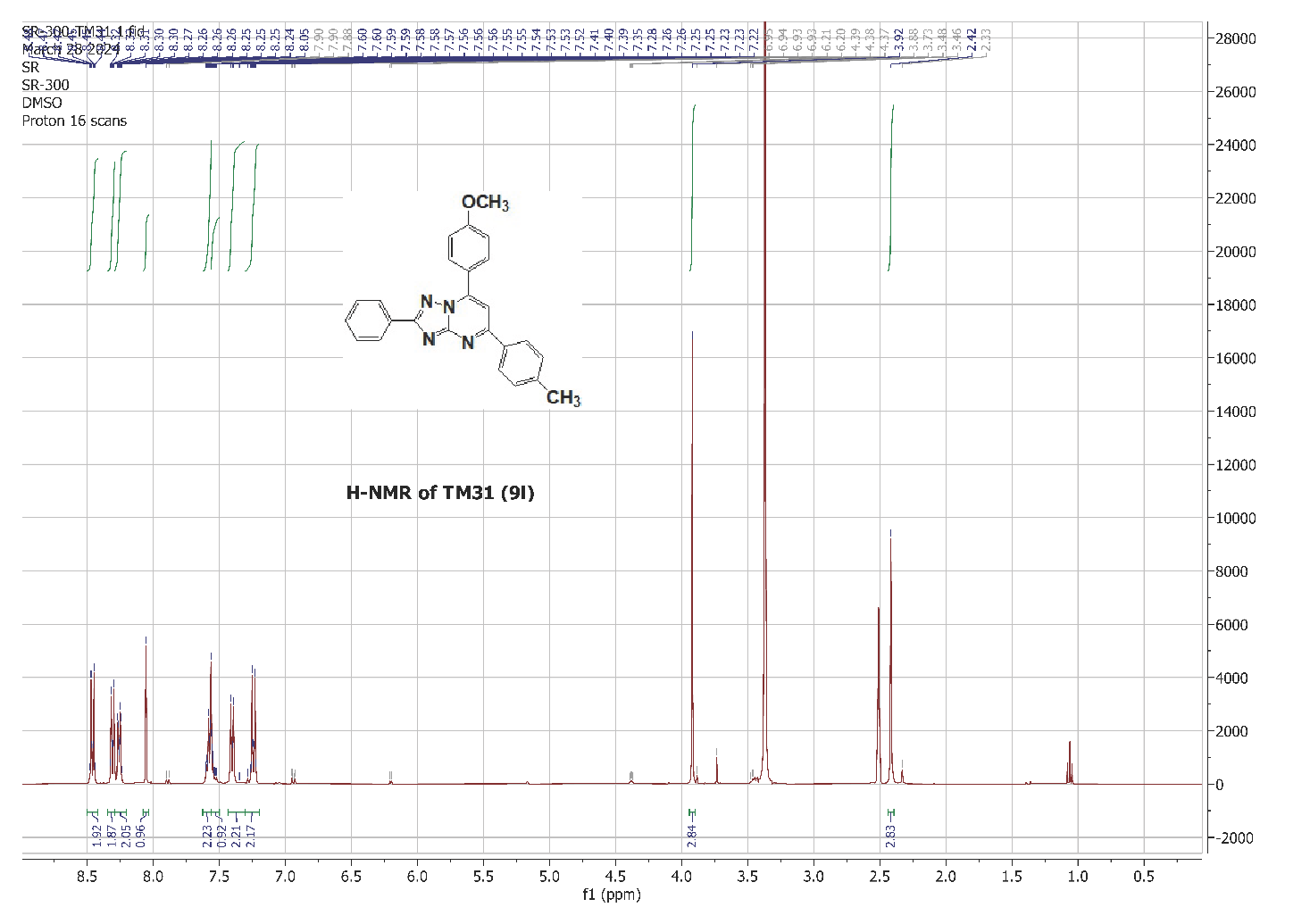
Figure S37**: ^1^H NMR spectrum (400 MHz, DMSO-*d*_6_) of compound **9l**

**
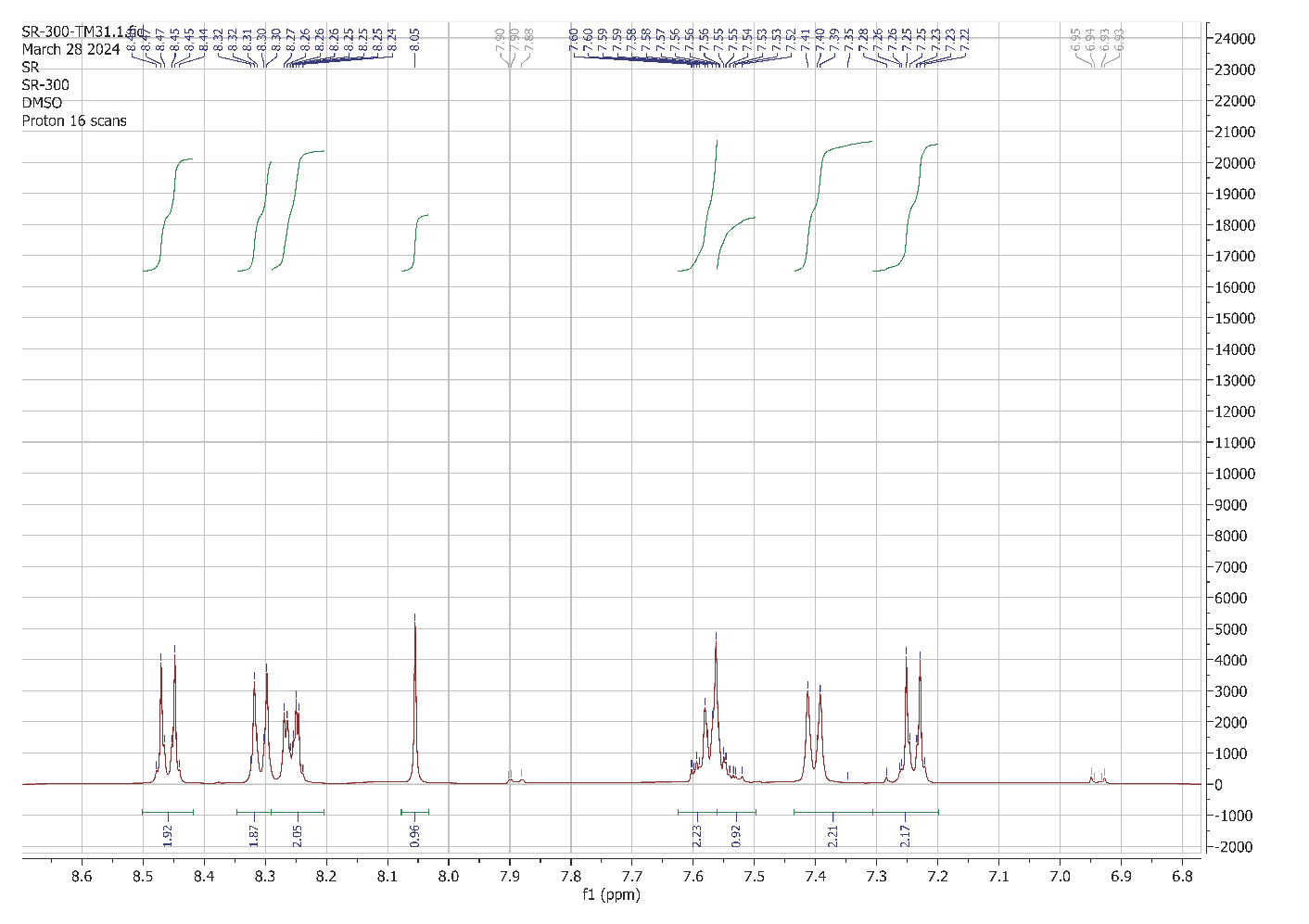
Figure S38**: Expanded ^1^H NMR spectrum (400 MHz, DMSO-*d*_6_) of compound **9l**

**
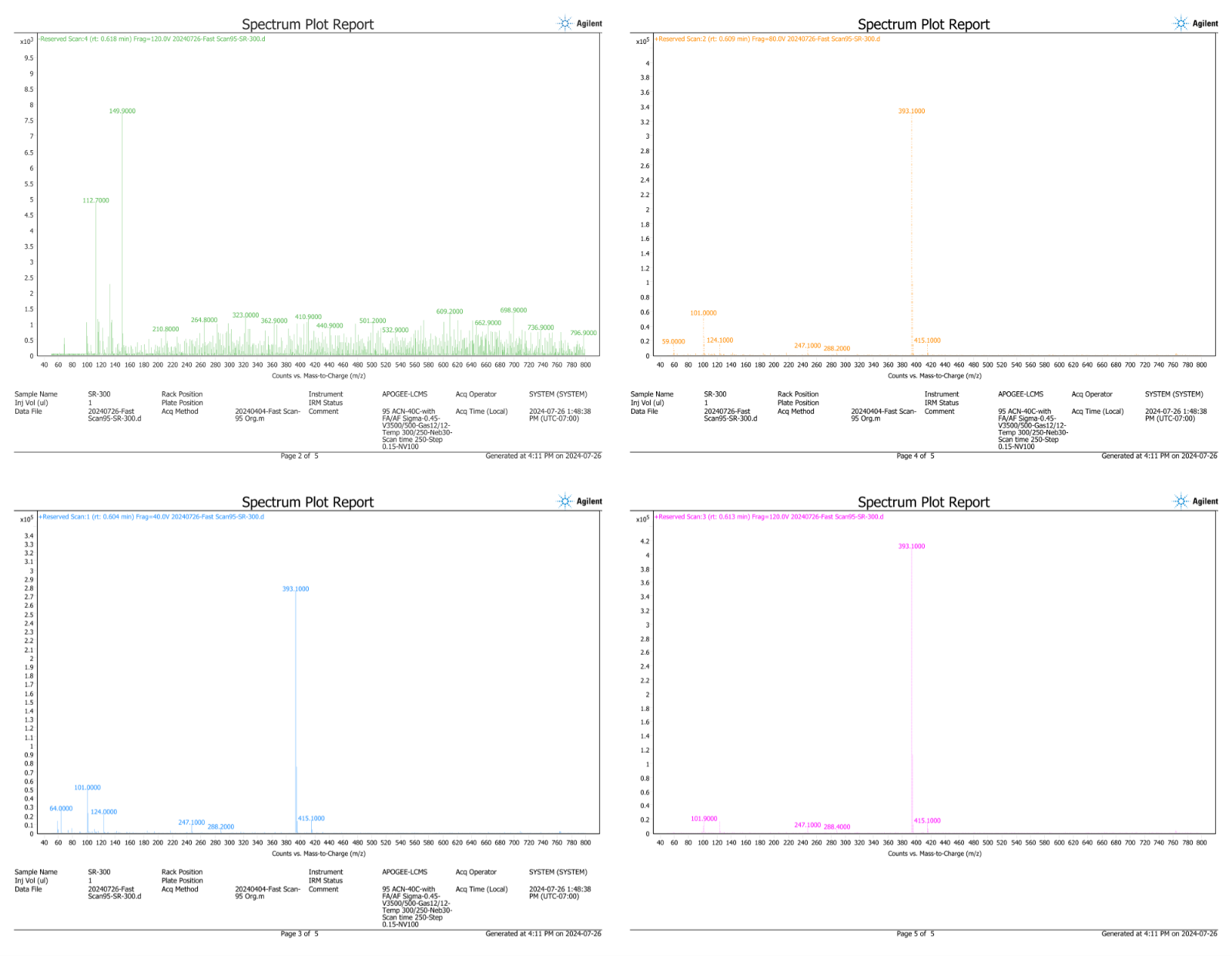
Figure S39**: LC-MS collated spectrum of compound **9l**

**
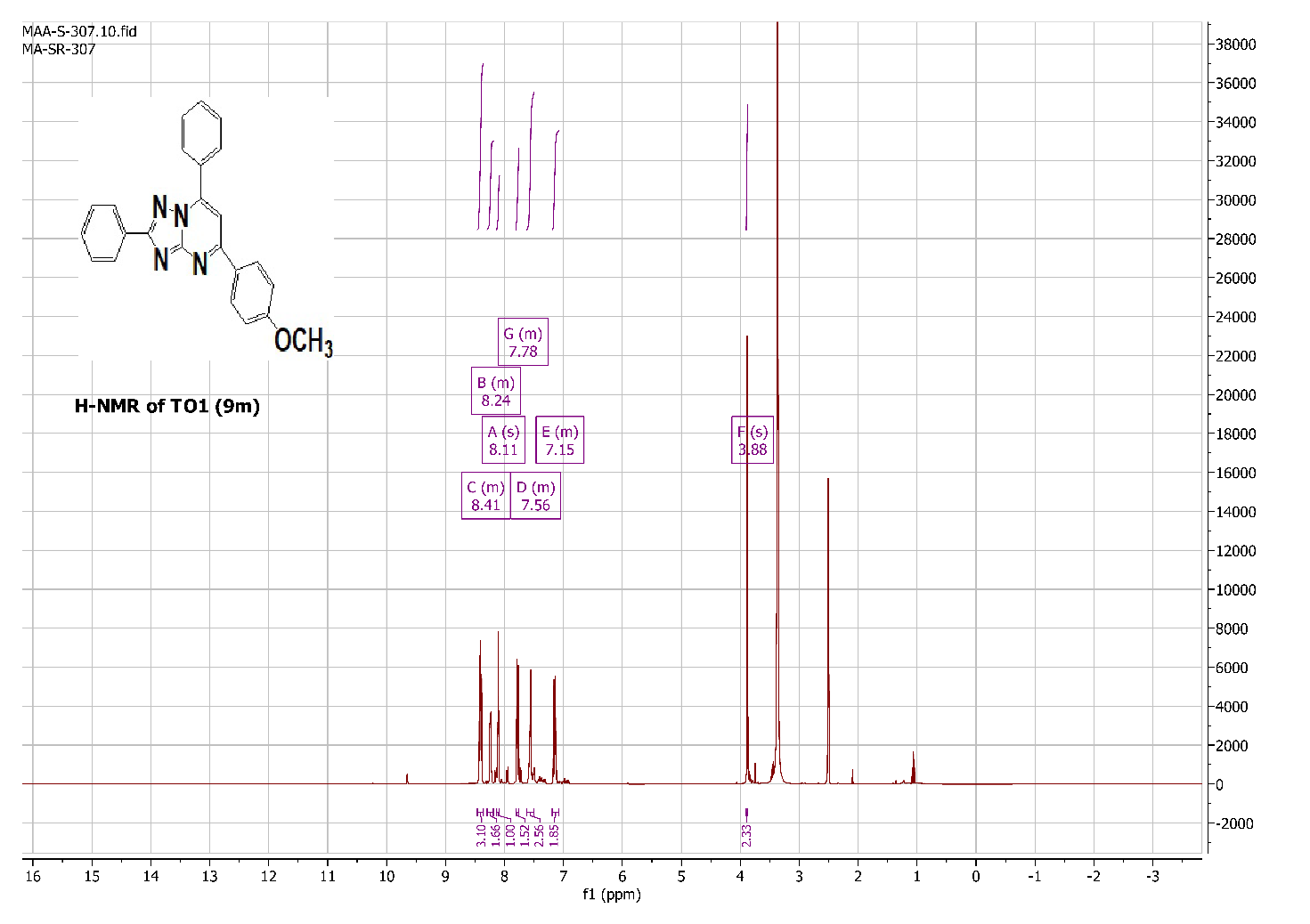
Figure S40**: ^1^H NMR spectrum (400 MHz, DMSO-*d*_6_) of compound **9m**

**
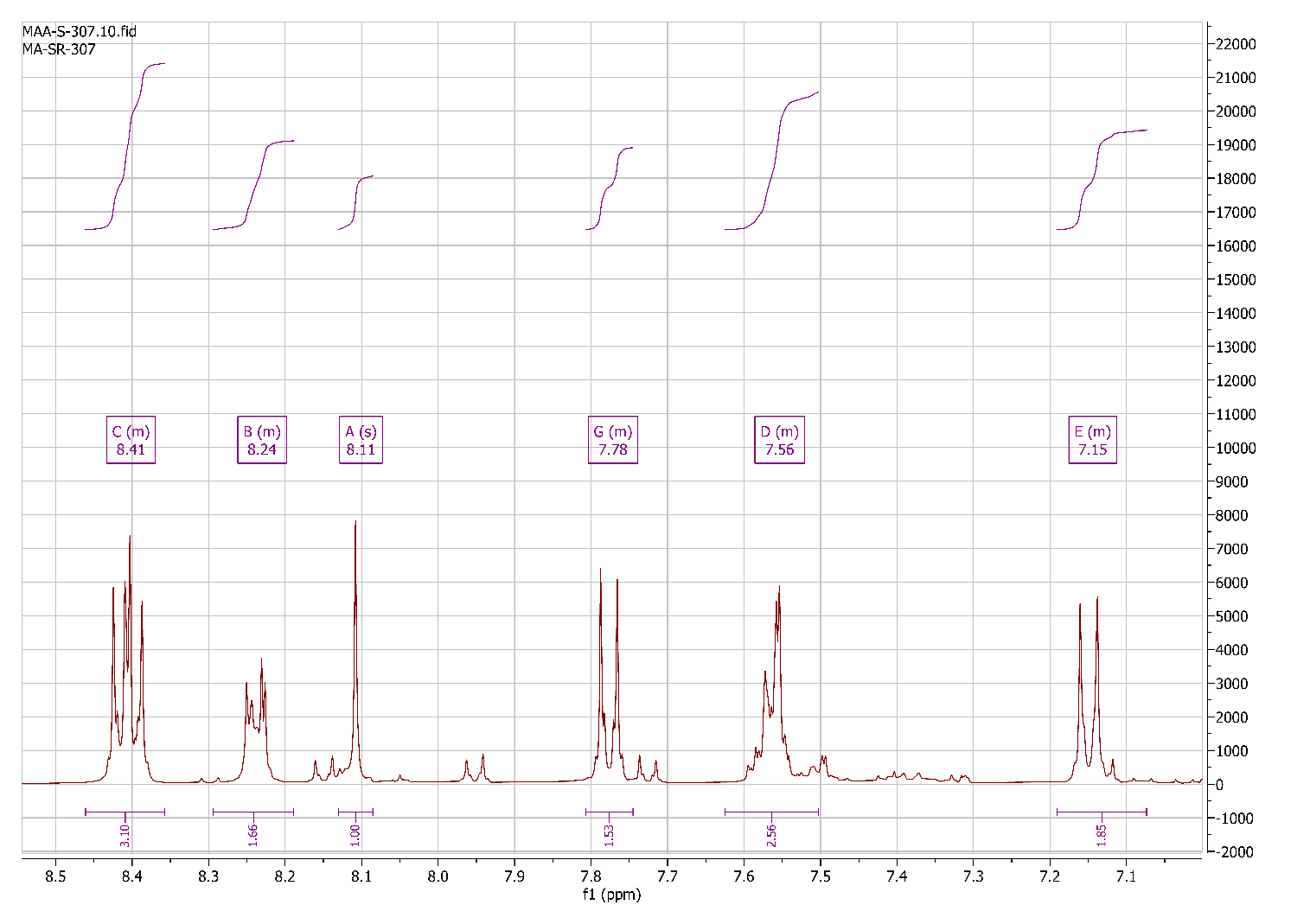
Figure S41**:Expanded ^1^H NMR spectrum (400 MHz, DMSO-*d*_6_) of compound **9m**

**Figure S42**: LC-MS collated spectrum of compound **9m**

**
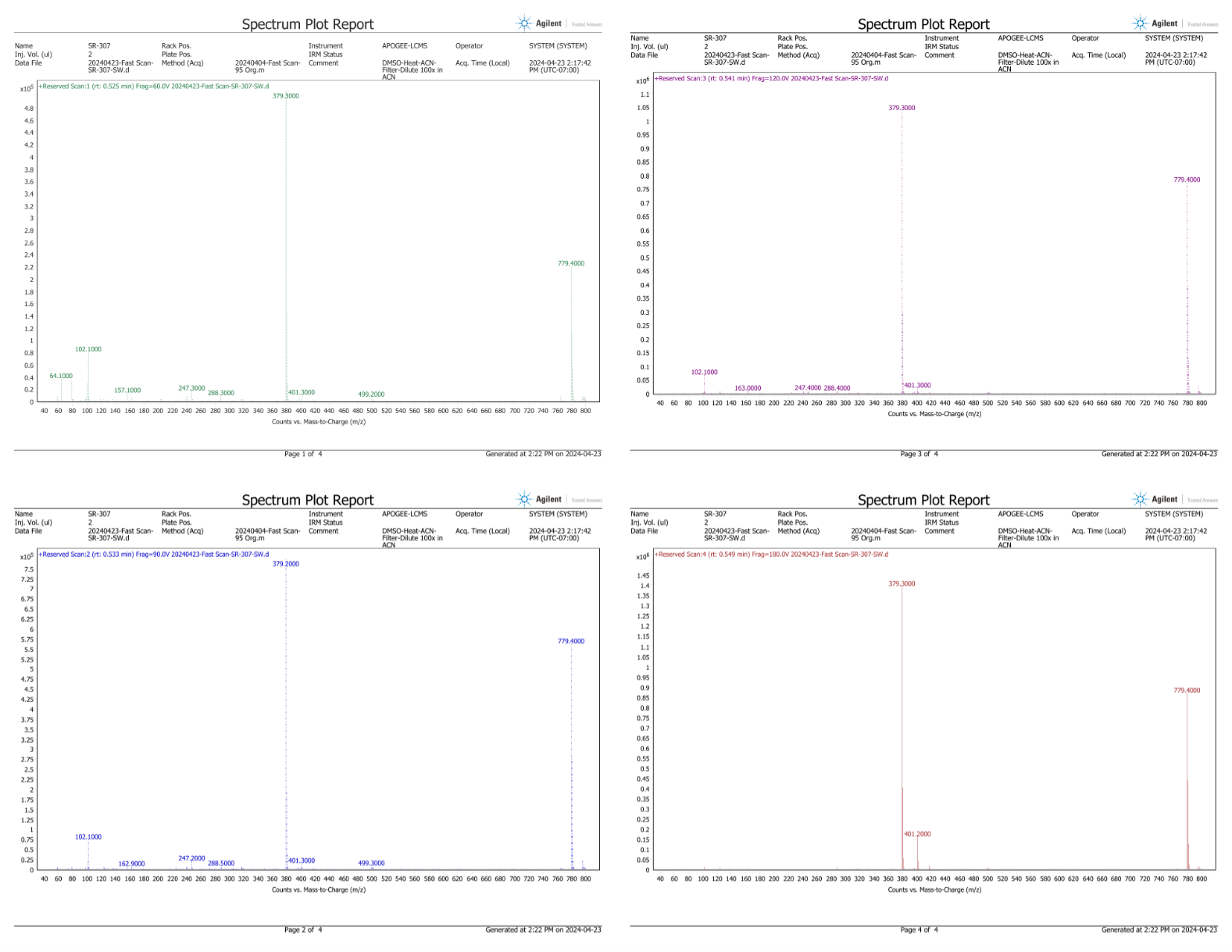
**

**Figure S43**: ^1^H NMR spectrum (400 MHz, DMSO-*d*_6_) of compound **9n**

**
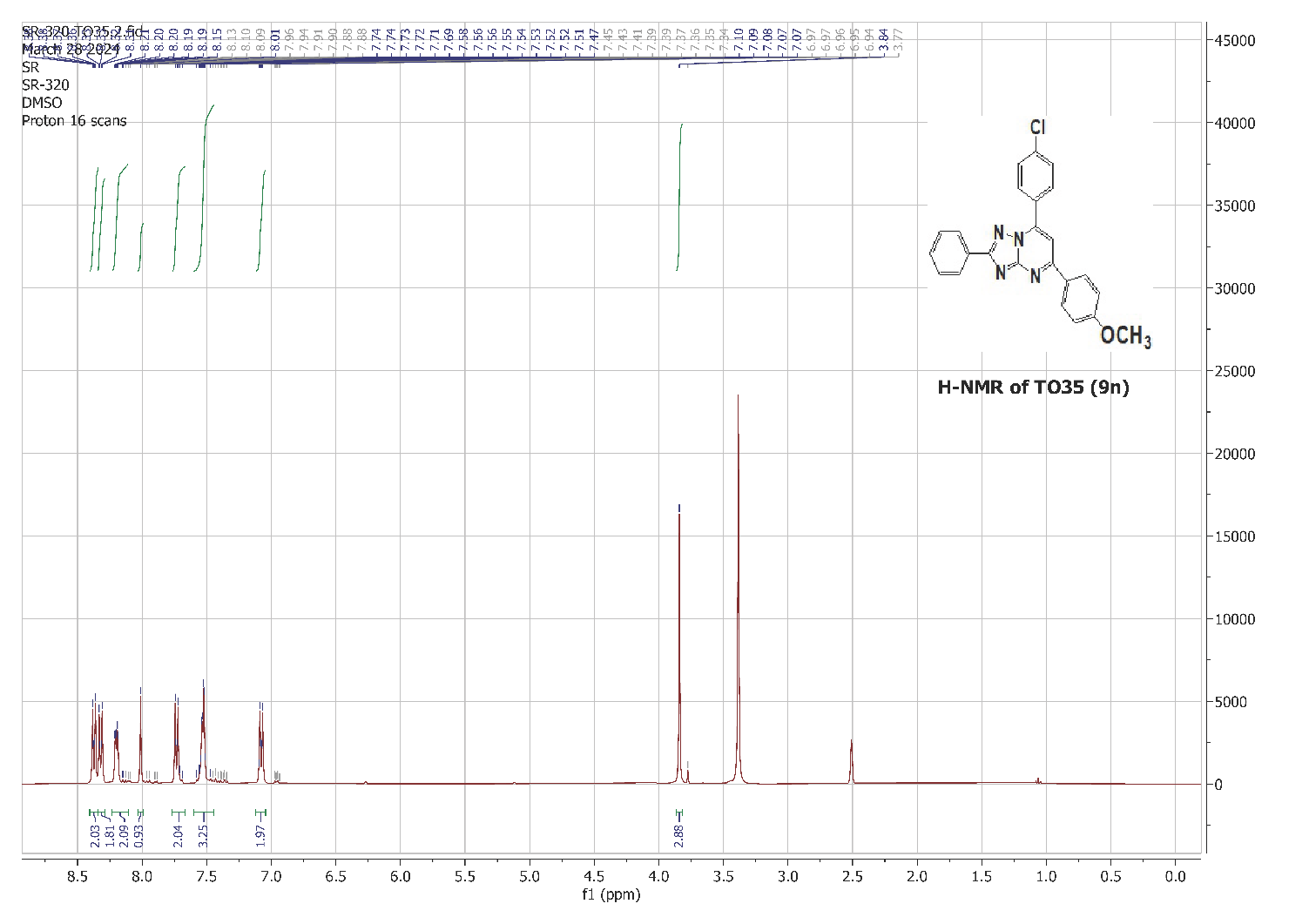
**

**
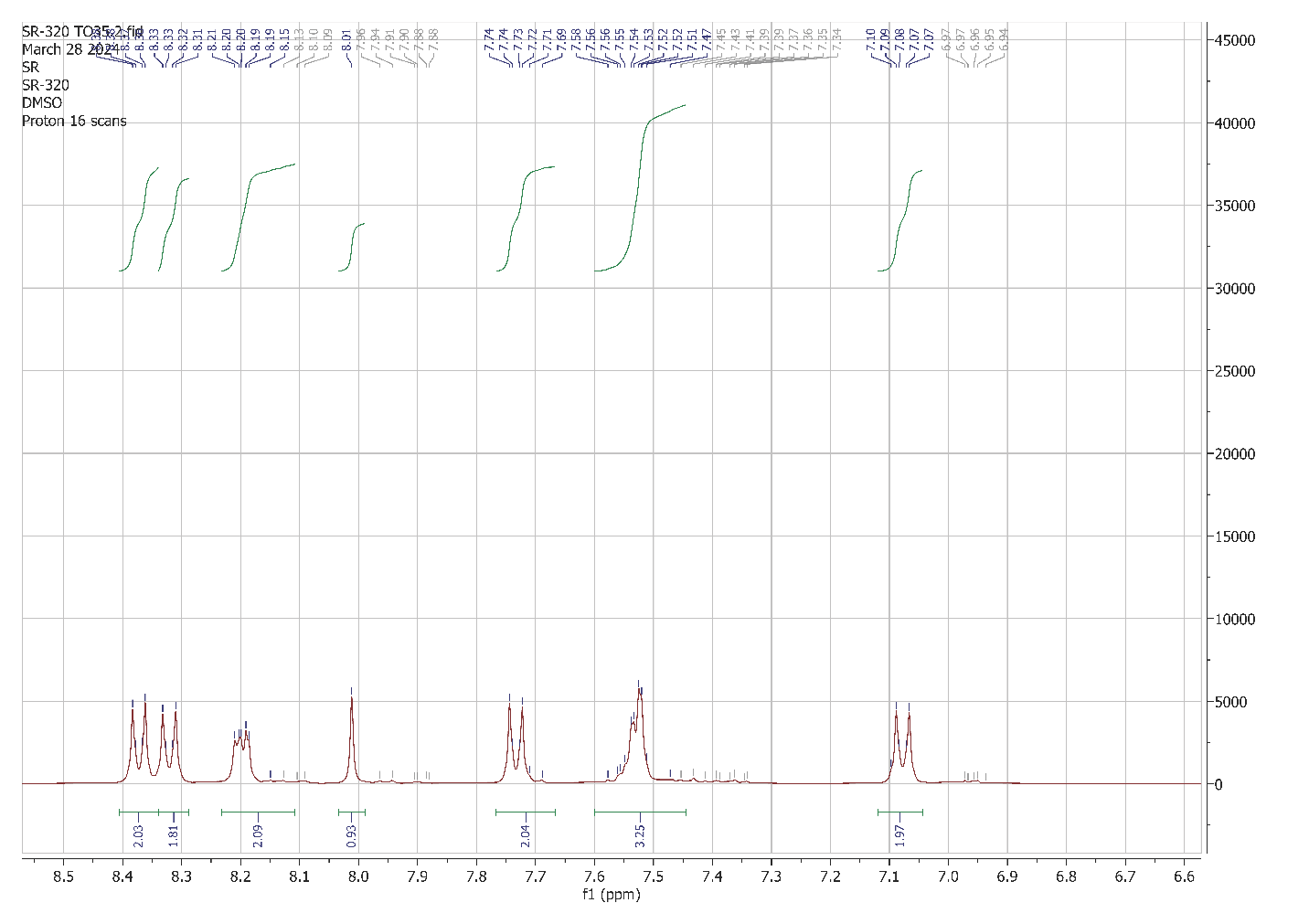
Figure S44**: Expanded ^1^H NMR spectrum (400 MHz, DMSO-*d*_6_) of compound **9n**

**
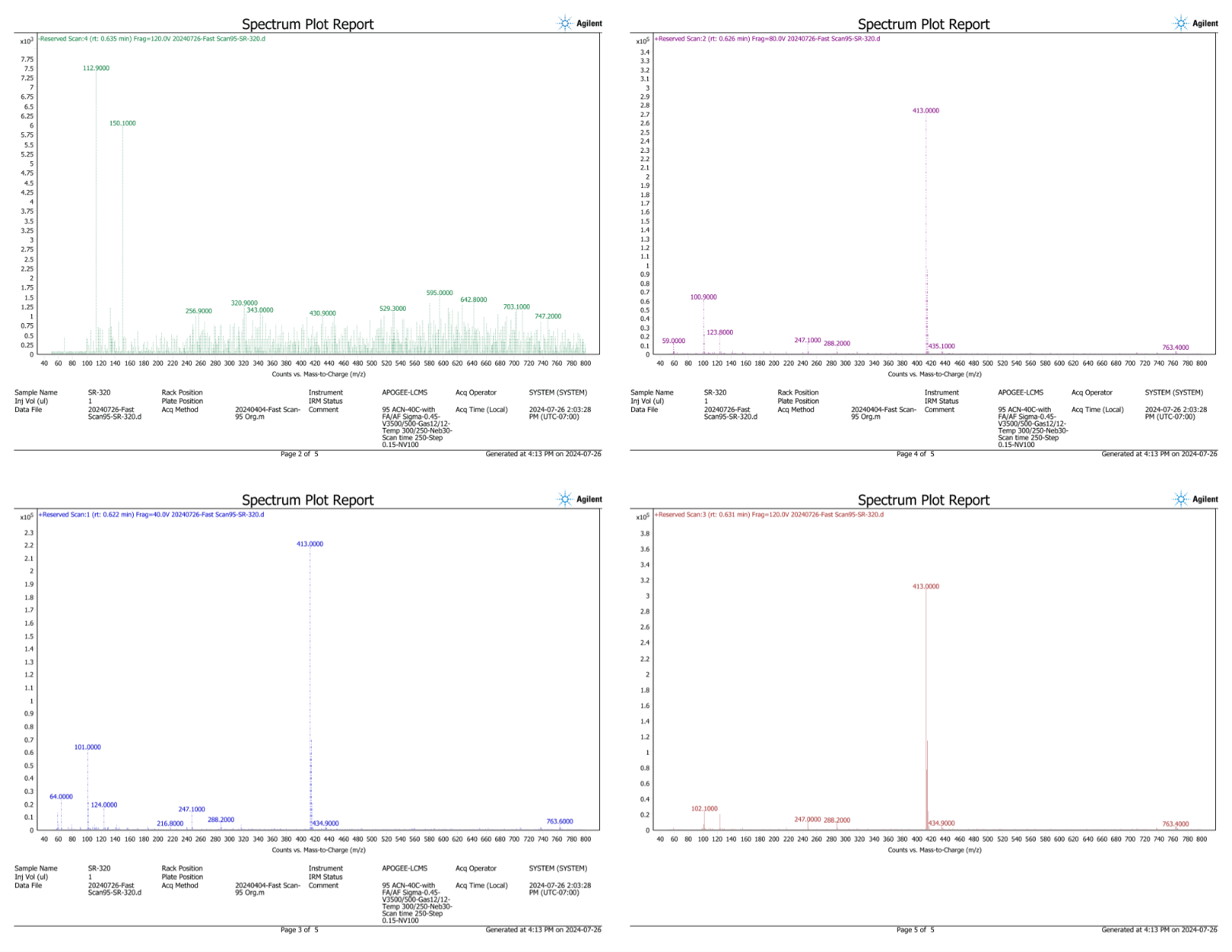
Figure S45**: LC-MS collated spectrum of compound **9n**

**
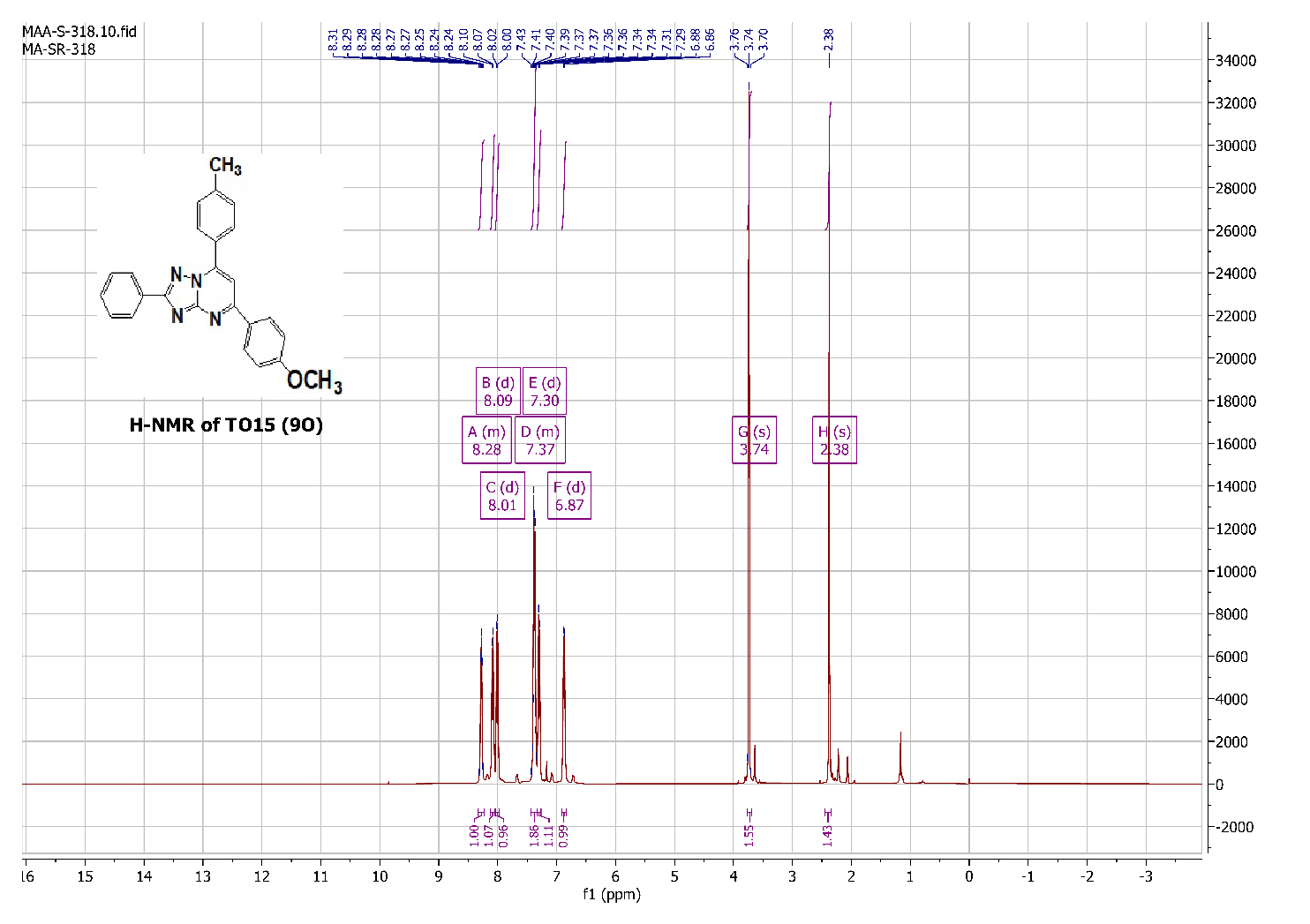
Figure S46**: ^1^H NMR spectrum (400 MHz, DMSO-*d*_6_) of compound **9o**

**Figure S47**: Expanded ^1^H NMR spectrum (400 MHz, DMSO-*d*_6_) of compound **9o**

**
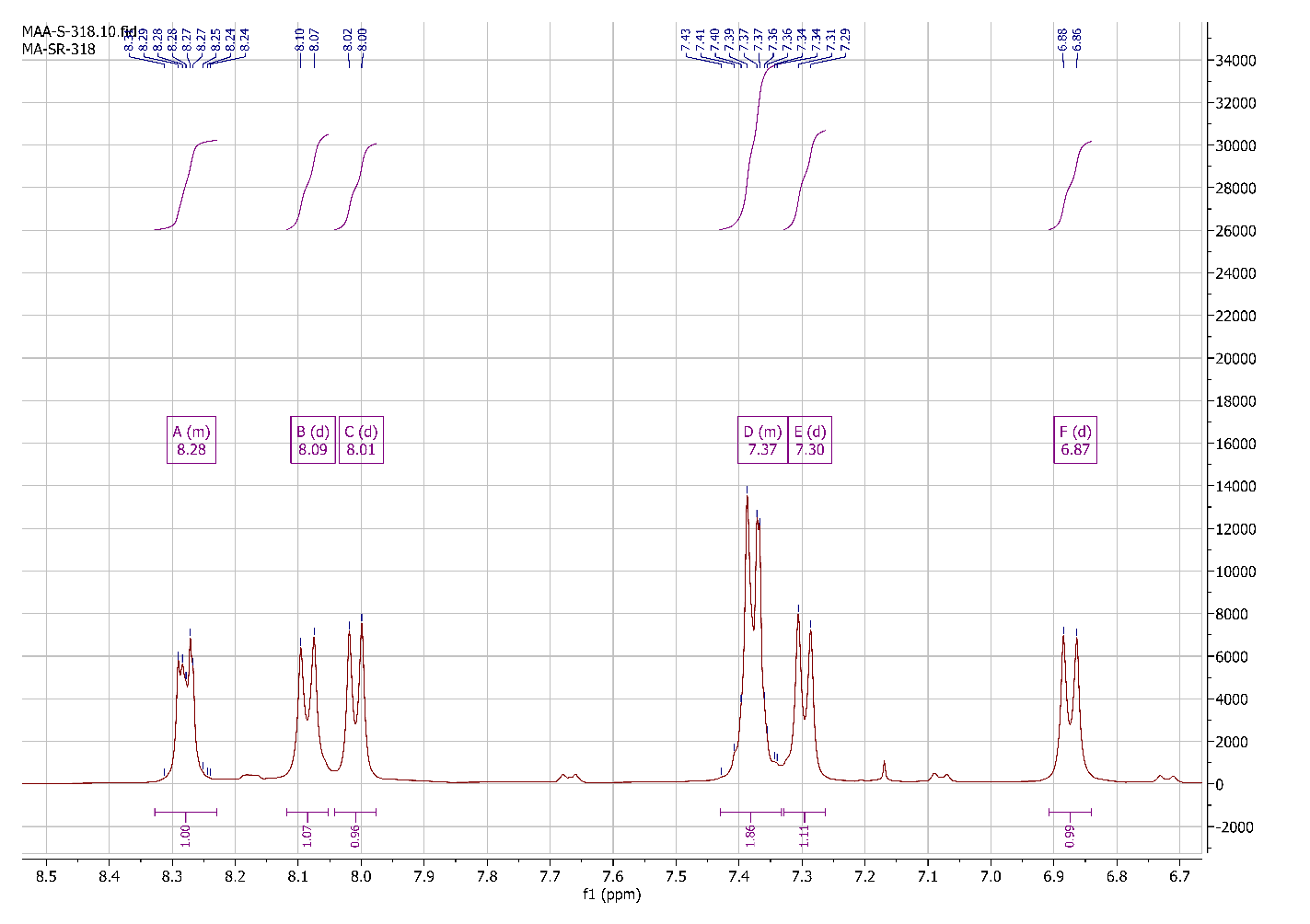
**

**Figure S48**: LC-MS collated spectrum of compound **9o**

**
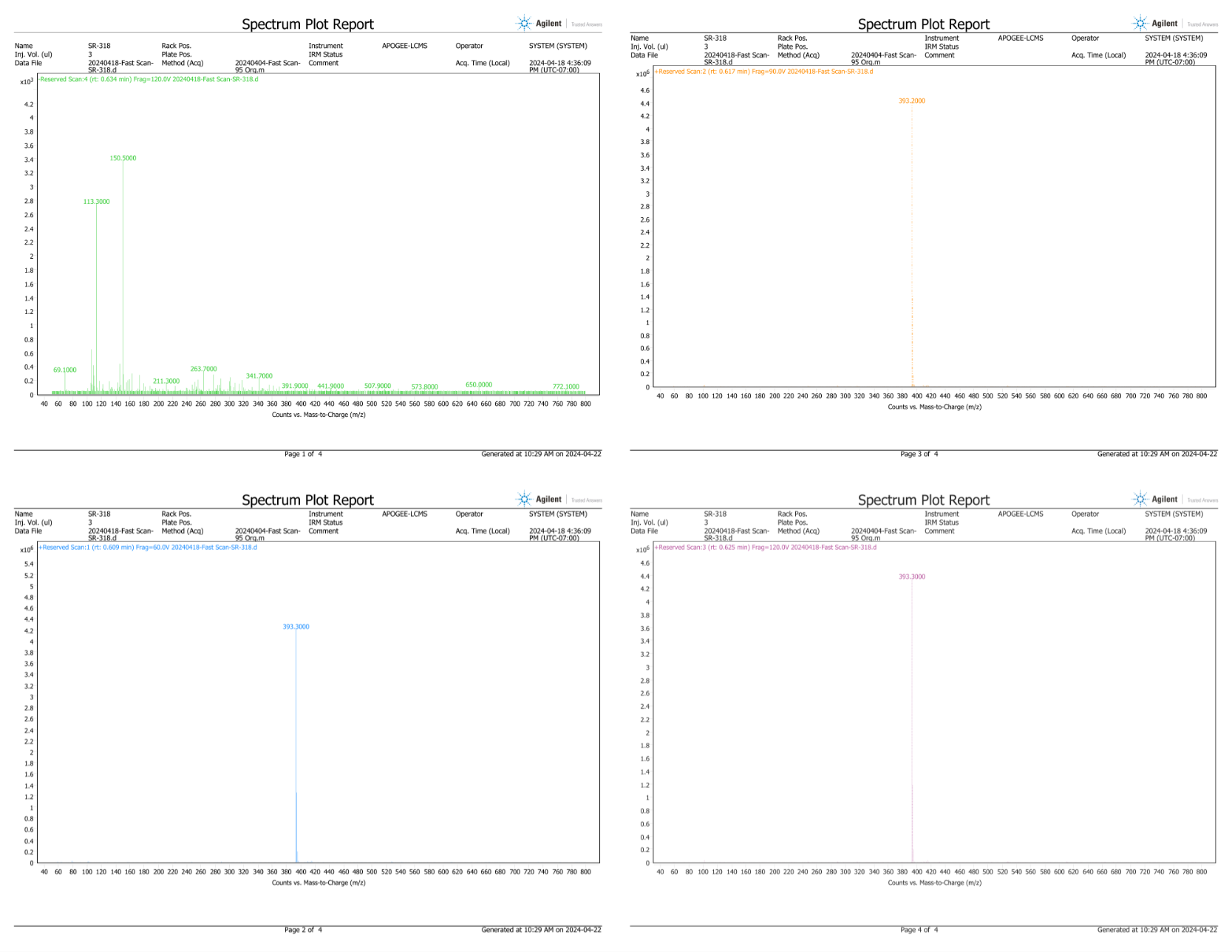
**

**
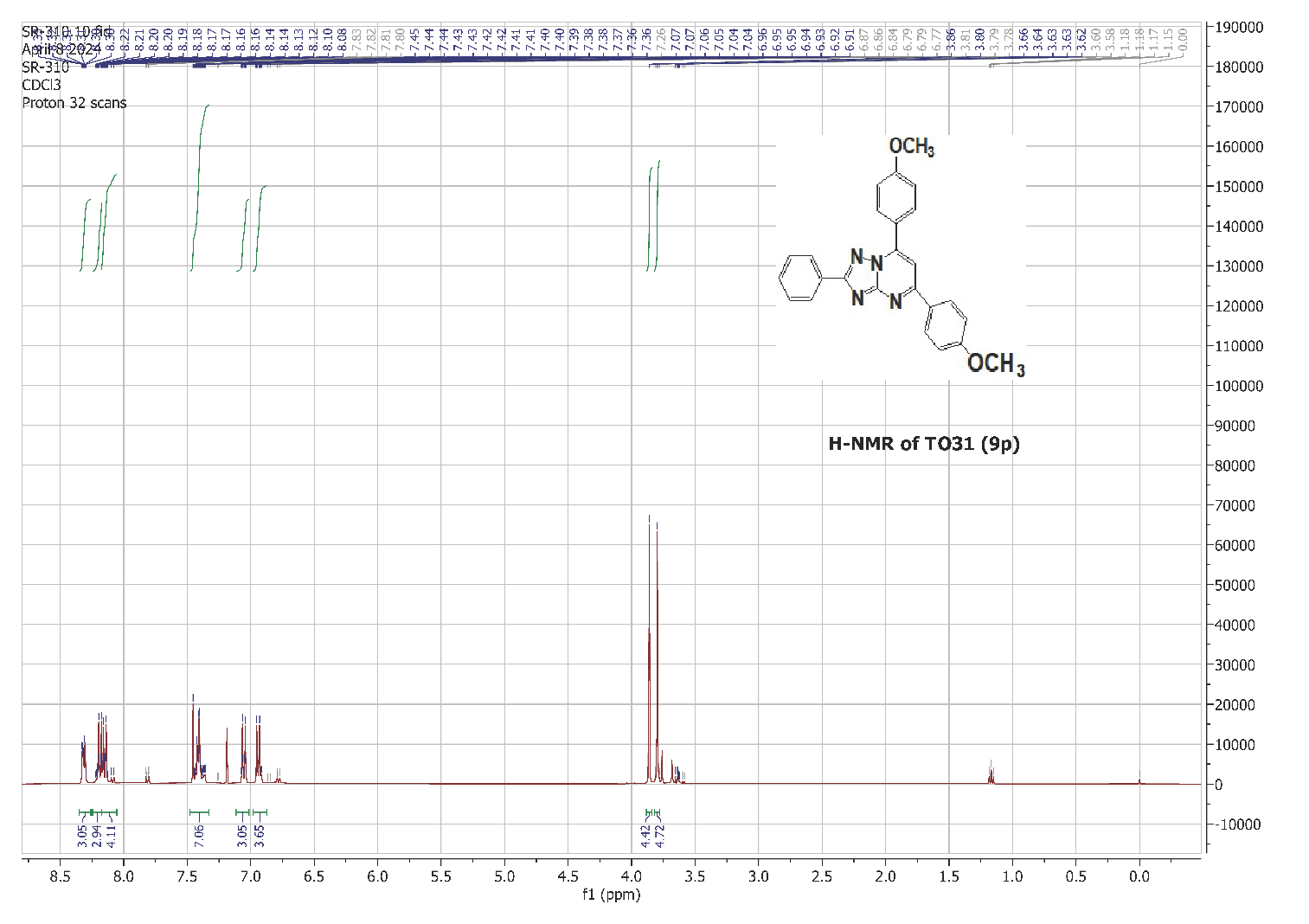
Figure S49**: ^1^H NMR spectrum (400 MHz, DMSO-*d*_6_) of compound **9p**

**Figure S50**: Expanded ^1^H NMR spectrum (400 MHz, DMSO-*d*_6_) of compound **9p**

**
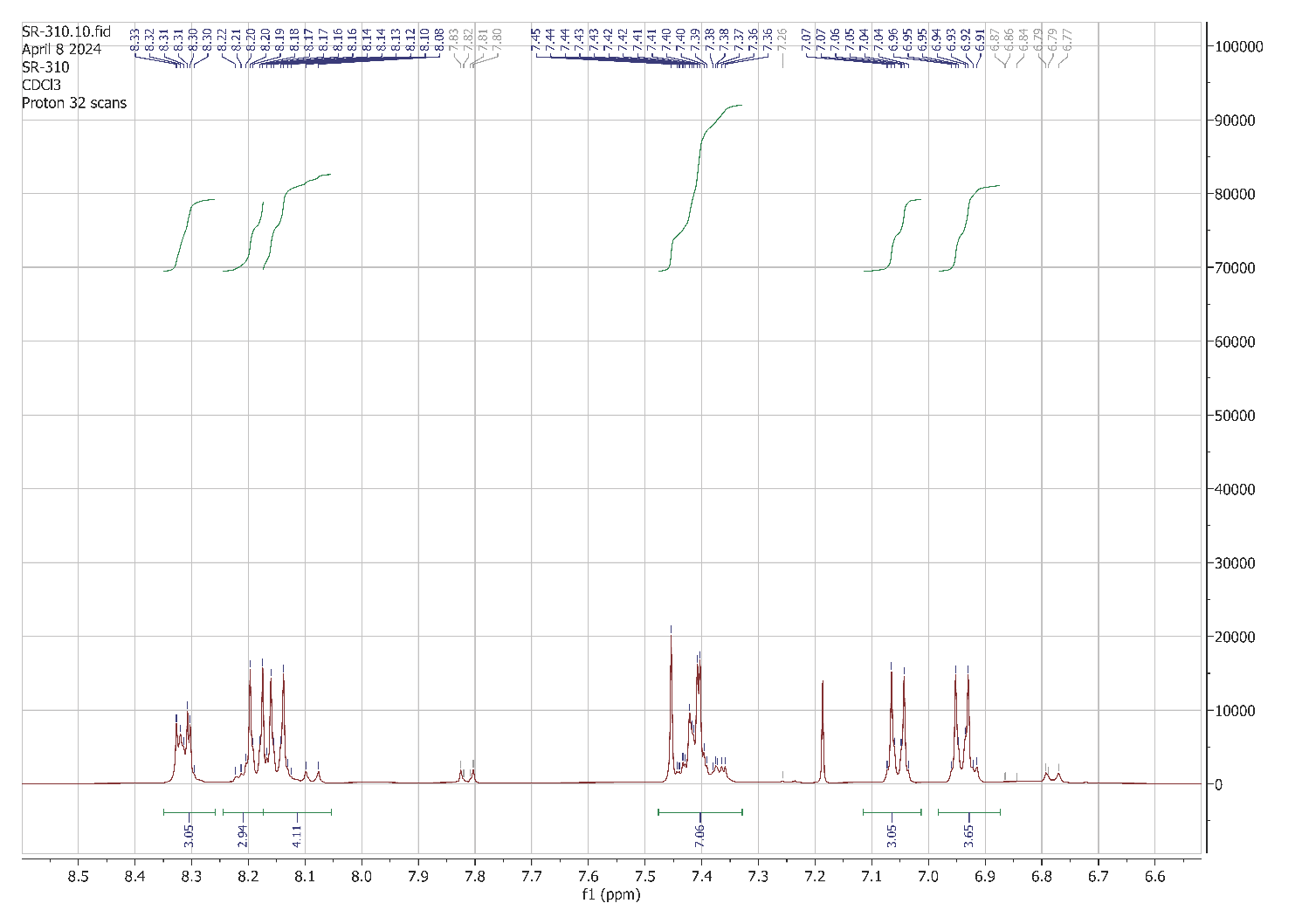
**


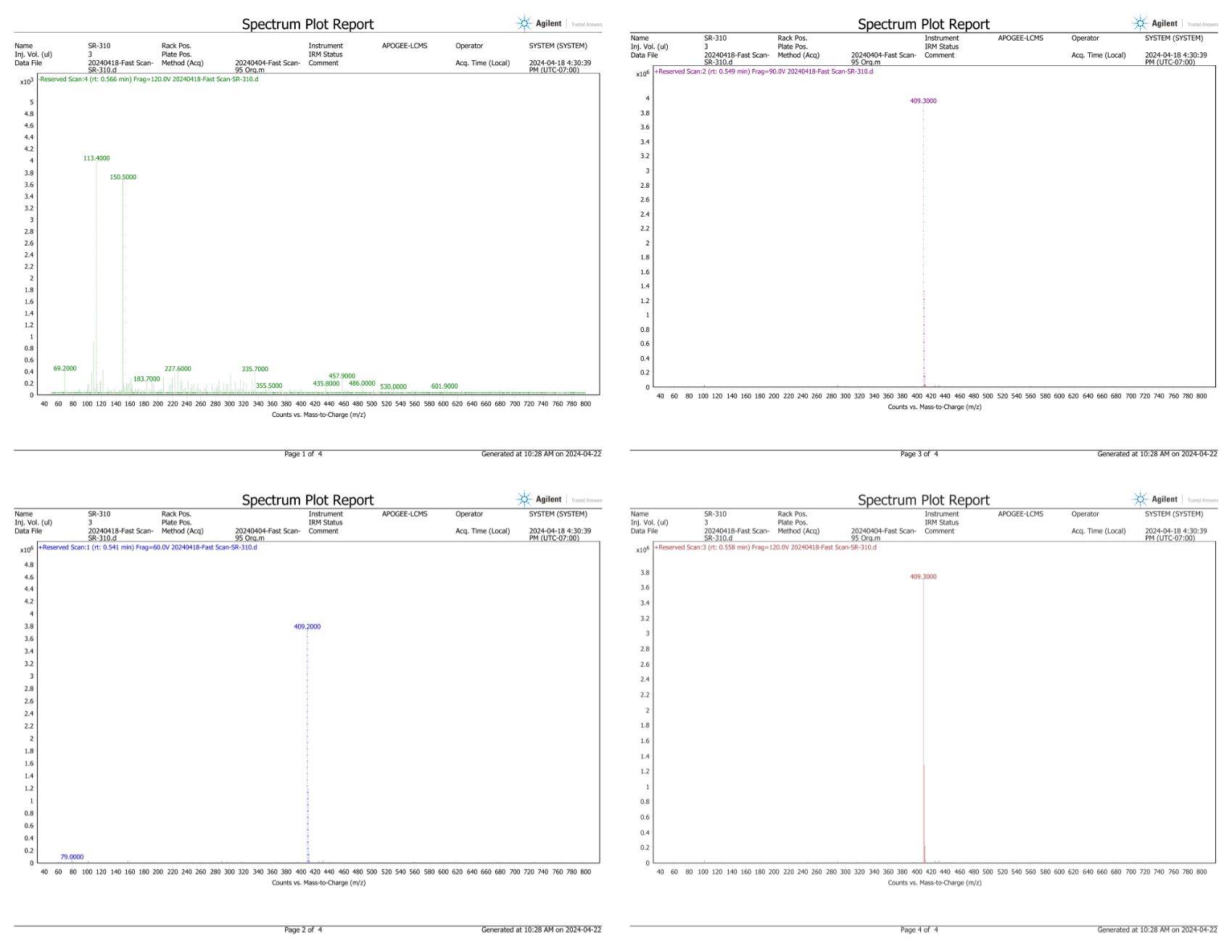
**Figure S51**: LC-MS collated spectrum of compound **9p**

**Appendix A**

**4. EXPERIMENTAL**

**4.1. Chemistry**

**General details:**

All chemicals were purchased from Sigma Aldrich, Combi-Blocks, Fisher Scientific and they were used without purification unless mentioned. ^1^H NMR spectra were recorded in DMSOd-6 at 400 MHz on a Bruker AC 400 Ultrashield 10 spectrophotometer. Chemical shifts are expressed in ppm, (δ scale). When peak multiplicities are reported, the following abbreviations are used: s (singlet), d (doublet), m (multiplet), dd (doublet of doublet). Coupling constants are reported in Hertz (Hz). Low- and high-resolution mass spectra were recorded on a AB Sciex UHPLC/MS/MS System and a Thermo Scientific Q Exactive Orbitrap High Resolution Mass.

**4.2. Antimicrobial activity**

**4.2.1. Organisms and culture conditions**

The cultures used were collected from the Cairo University's Microanalytical Centre, Faculty of Science. An updated Kirby-Bauer disc diffusion method was applied for antimicrobial activities of the tested compounds [28]. Shortly, the 10 ml of fresh medium was grown to 100 μl bacteria / food until a count of 108 cell / ml or 105 cell / ml was achieved [29]. 100 μl microbial suspension has been spread over agar plates that suit the broth in which it was held. Selected colonies of each organism that may play a pathogenic function should be from the primary agar plates and tested by the disc diffusion method for susceptibility [30, 31]. Plates inoculated with filamentous fungi *as Aspergillus flavus* at 25^o^C for 48 hours; Gram positive bacteria as *Staphylococcus aureus* (ATCC 12600)*, Bacillus subtilis* (ATCC 6051); Gram negative bacteria as *Escherichia coli* (ATCC 11775), *Pseudomonas aeuroginosa* (ATCC 10145) they were incubated at 35-37^o^C for 24-48 hours and yeast as *Candida albicans* (ATCC 7102) incubated at 30^o^C for 24-48 hours and, then the diameters of the inhibition zones were measured in millimeters. Standard discs of ciprofloxacin (Antibacterial agent), Fluconazole (Antifungal agent) served as positive controls for antimicrobial activity but filter discs impregnated with 10 µl of solvent (distilled water, chloroform, DMSO) were used as a negative control. Blank paper disks (Schleicher & Schuell, Spain) with a diameter of 8.0 mm were impregnated 10µ of tested concentration of the stock solutions. When a filter paper disc impregnated with a tested chemical is placed on agar the chemical will diffuse from the disc into the agar. This diffusion will place the chemical in the agar only around the disc. The solubility of the chemical and its molecular size will determine the size of the area of chemical infiltration around the disc. If an organism is placed on the agar it will not grow in the area around the disc if it is susceptible to the chemical. This area of no growth around the disc is known as a “Zone of inhibition” or" Clear zone". For the disc diffusion, the zone diameters were measured with slipping calipers of the National Committee for Clinical Laboratory Standards [30], and the results are given in **Table 1**. Agar-based methods such as E-test and disk diffusion can be good alternatives because they are simpler and faster than broth-based methods [30, 31].

**4.2.2. Minimum inhibitory concentration assay**

In 96-well microtiter plates and 50 mL of fresh bacterial culture of a single McFarland unit overnight, a double serial dilution of each compound (100 mL) in sterile standard saline were prepared to every single source well. Ciprofloxacin antibiotic (5 mg / mL-1) and normal saline were included as standard reference in each assay. The plates were incubated at 37 ^0^C overnight. As an indicator of bacterial growth, 40 mL of p-iodonitrotetrazolium violet (INT) was added to each well and incubated at 37 ^0^C for 30 min. MIC values are recorded as the lowest concentration of the extract that completely inhibited bacterial growth that is clear well. The colorless tetrazolidium salt acts as an electron accepter and is reduced to a red colored formazan product by biological activity organisms. Where bacterial growth was inhibited, the solution in the well remained clear after incubation with INT. The observed MIC values are presented in **Table 2**.

**4.2.3. Determination of Inhibitory Activities on *E. coli* DNA Gyrase DHFR**.

All the final compounds were tested for *E. coli* DNA gyrase inhibitory activity in a supercoiling assay. Activities were determined on streptavidin-coated 96-well microtiter plates from Thermo scientific Pierce. First, the plates were rehydrated with buffer (20 mM Tris-HCl with pH 7.6, 0.01% w/v BSA, 0.05% v/v Tween 20, 137 mM NaCl) and the biotinylated oligonucleotide was then immobilized. After washing off the unbound oligonucleotide, the enzyme test was performed. The reaction volume of 30 μL in buffer (35 mM Tris-HCl with pH 7.5, 4 mM MgCl_2_, 24 mM KCl, 2 mM DTT, 1.8 mM spermidine, 1 mM ATP, 6.5 % w/v glycerol, 0.1 mg/mL albumin) contained 1.5 U of DNA gyrase from *E. coli* or *S. aureus*, 0.75 μg of relaxed pNO1 plasmid, and 3 μL solution of the inhibitor in 10% DMSO and 0.008% Tween 20. Reaction solutions were incubated at 37 °C for 30 min. After that, the TF buffer (50 mM NaOAc with pH 5.0, 50 mM NaCl and 50 mM MgCl_2_) was added to terminate the enzymatic reaction. After additional incubation for 30 min at rt, during which biotin-oligonucleotide-plasmid triplex was formed, the unbound plasmid was washed off using TF buffer and SybrGOLD in T10 buffer (10 mM Tris HCl with pH 8.0 and 1 mM EDTA) was added. The fluorescence was measured with a microplate reader (BioTek Synergy H4, excitation: 485 nm, emission: 535 nm). Initial screening was done at 100 or 10 μM concentration of inhibitors. For the most active inhibitors IC_50_ was determined using seven concentrations of tested compounds. GraphPad Prism software was used to calculate the IC_50_ values. The result is given as the average value of three independent measurements. As the internal standard novobiocin (IC_50_ = 0.168 µM for *E. coli* gyrase and IC_50_ = 0.041 µM for *S. aureus* gyrase) was used. Determination of inhibitory activities on *E. coli* and *S. aureus* Topoisomerase IV. IC_50_ values were determined in an assay from In spiralis on streptavidin-coated 96-well microtiter plates from Thermo scientific Pierce. First, the plates were rehydrated with buffer (20 µM Tris-HCl with pH 7.6, 0.01% w/v BSA, 0.05% v/v Tween 20, 137 mM NaCl) and biotinylated oligonucleotide was then immobilized. After washing off the unbound oligonucleotide, the enzyme test was performed. The reaction volume of 30 μL in buffer (40 mM HEPES KOH with pH 7.6, 100 mM potassium glutamate, 10 mM magnesium acetate, 10 mM DTT, 1 mM ATP, 0.05 mg/mL albumin) contained 1.5 U of topoisomerase IV from *E. coli* or *S. aureus*, 0.75 μg of pNO1 supercoiled plasmid, and 3 μL solution of the inhibitor in DMSO (10%) and Tween 20 (0.008%). Reaction mixtures were incubated at 37 °C for 30 min and after that, the TF buffer (50 mM NaOAc with pH 5.0, 50 mM NaCl and 50 mM MgCl_2_) was added to terminate the enzymatic reaction. After additional incubation for 30 min at rt, during which triplex (biotin-oligonucleotide-plasmid) was formed, the unbound plasmid was washed off using TF buffer and Sybr GOLD in T10 buffer (10 mM Tris HCl with pH 8.0 and 1 mM EDTA) was added. The fluorescence was measured with a microplate reader (BioTek Synergy H4, excitation: 485 nm, emission: 535 nm). Initial screening was done at 100 or 10 μM concentration of inhibitors. For the most active inhibitors IC_50_ was determined using seven concentrations of tested compounds. GraphPad Prism software was used to calculate the IC_50_ values. The result is given as the average value of three independent measurements. As the internal standard novobiocin (IC_50_ = 11.1 µM) for *E. coli* topoisomerase IV and IC_50_ = 26.7 µM for *S. aureus* topoisomerase IV) was used.
